# Design and synthesis of chiral DOTA-based MRI contrast agents with remarkable relaxivities

**DOI:** 10.1038/s42004-023-01050-w

**Published:** 2023-11-16

**Authors:** Junhui Zhang, Lixiong Dai, Li He, Abhisek Bhattarai, Chun-Ming Chan, William Chi-Shing Tai, Varut Vardhanabhuti, Ga-Lai Law

**Affiliations:** 1 State Key Laboratory of Chemical Biology and Drug Discovery, Department of Applied Biology and Chemical Technology, The Hong Kong Polytechnic University, Hung Hom, Hong Kong SAR, China; 2 The Hong Kong Polytechnic University Shenzhen Research Institute, Shenzhen, 518000 China; 3 Department of Diagnostic Radiology, Li Ka Shing Faculty of Medicine, The University of Hong Kong, Hong Kong SAR, China

**Keywords:** Chemical tools, Medical and clinical diagnostics, Imaging studies, Biomedical materials, Ligands

## Abstract

Due to the adverse effects of de-metallation in past concerning FDA-approved gadolinium-based contrast agents (GBCAs), researchers have been focusing on developing safer and more efficient alternatives that could avoid toxicity caused by free gadolinium ions. Herein, two chiral GBCAs, Gd-LS with sulfonate groups and Gd-T with hydroxyl groups, are reported as potential candidates for magnetic reasonance imaging (MRI). The *r*_1_ relaxivities of TSAP, SAP isomers of Gd-LS and SAP isomer of Gd-T at 1.4 T, 37 °C in water are 7.4 mM^−1^s^−1^, 14.5 mM^−1^s^−1^ and 5.2 mM^−1^s^−1^, respectively. Results show that the hydrophilic functional groups introduced to the chiral macrocyclic scaffold of Gd-T and Gd-LS both give constructive influences on the second-sphere relaxivity and enhance the overall *r*_1_ value. Both cases indicate that the design of GBCAs should also focus on the optimal window in Solomon-Bloembergen-Morgan (SBM) theory and the effects caused by the second-sphere and outer-sphere relaxivity.

## Abstract (web summary)

Gadolinium-based contrast agents are the gold standard for magnetic resonance imaging (MRI), however, their poor stability causes safety problems in clinical applications. Here, the authors develop two chiral Gd(III) DOTA-based complexes with a macrocyclic backbone that show higher relaxivities and stabilities than benchmark complexes.

## Introduction

Magnetic resonance imaging (MRI) is one of the most widely used diagnostic techniques clinically, as it is beneficial for the scanning and detection of abnormalities in soft tissue and organs. Due to its low sensitivity, about one-third of MRI studies performed are contrast-enhanced with gadolinium-based contrast agents (GBCAs), which are the mainstream MRI contrast agents used due to their high efficiency and minimal risk. GBCAs are considered relatively safe to most patients in terms of invasiveness and toxicity^1,2^. However, since the identification of an adverse effect of nephrogenic systemic fibrosis (NSF) in 2006 and more recent findings about Gd deposition in patients’ brains^3,4^, research interest in the developing and searching of better alternatives for a more stable and efficient class of contrast agents has resurged^5,6^.

**Fig. 1:**
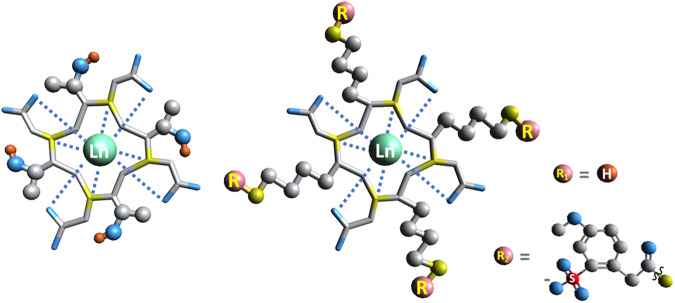
The chemical structures of [Ln-T]^-^ and [Gd-LS]^-^ studied in this work. Ln represents Eu(III) and Gd(III), counter-ions, NH_4_^+^ or H^+^ are neglected for clarity.

Compared to the parent Gd-DOTA (Gadolinium (III) 1,4,7,10-Tetraazacyclododecane-1,4,7,10-tetraacetate, currently one of the benchmarks of clinically used GBCAs), we have recently reported a series of chiral Gd-DOTAs that shows better stability. The water-exchange rates of these compounds are also in the optimal range for enhancing the longitudinal (*T*_1_) relaxation of water under high magnetic field^7^. This initial proof of concept design serves as a platform for relaxivity improvement, as well as the specificity of our Gd(III) complexes to target organs of interest. By improving the efficiency of these compounds, the dosage required can then be lowered, and the quantity of Gd(III) metal required for a successful scan can be reduced^8^. The longitudinal relaxivity (*r*_1_) of a contrast agent is quantified by the degree of enhancement of the water proton relaxation rate (*T*_1_^−1^) normalized to the concentration of the paramagnetic solute^9^. The relaxivity of a Gd(III) complex can be the sum of inner-sphere relaxivity, second-sphere relaxivity, and outer-sphere relaxivity^1^. Practically, the most successful strategy to increase the relaxivity of a contrast agent is through prolonging the rotational correlation time (*τ*_R_) to achieve the enhancement of the inner-sphere relaxivity^10^. The second-sphere relaxivity contribution is generally considered to be low, however, when Gd(III) ions are close to some hydrophilic functional groups such as phosphate and hydroxyl groups, a non-negligible effect might occur^11^. Herein, two chiral DOTA Gd(III) complexes (Gd-LS and Gd-T as shown in Fig. 1), which have higher relaxivity than Gd-DOTA, are presented. Through utilizing the design strategies on the macrocyclic backbone, the performances of these Gd(III) complexes are improved as a result of relaxivity enhancement in the first-sphere or/and second-sphere.

## Results and discussion

### Design and synthesis

For Ln-LS, this is based on our earlier designed compound, Ln-L, which is reported in our previous publication^7^. Here, Ligand L is used as an intermediate for ligand LS. Ligand L, is synthesized from the starting material of *L*-Lysine to form four aminobutyl groups around the macrocyclic chelator. This compound is unlike other chiral DOTA complexes with alkyl or aromatic substituents, since the four aminobutyl groups cause the major coordination isomer of Ln-L to reverse from twist square antiprism (TSAP) to square antiprism (SAP). The SAP isomer of Gd-L gave faster water-exchange rate than Gd-DOTA. The biodistribution study shows that Gd-L has a specific kidney localisation as well as good retention and clearance profile, hence it is selected for optimization to give Gd-LS.

In Gd-LS, four aromatic groups with sulfonic acid substitution are conjugated onto the aminobutyl groups, resulting in a negatively charged complex with a much higher molecular weight than Gd-L. We propose this structural change might result in better water solubility, making it feasible in biological application. The increase in molecular weight should also result in higher relaxivity values due to the decrease of molecular tumbling rate (*τ*_R_)^12^. It is also predicted that the aromatic rings may bind to the HSA protein in blood plasma via weak interactions, such as π-π interactions. Hence, the relaxivity in the blood may increase. In another design, Gd-T, is synthesized from the starting material of *L*-Threonine to give four hydroxy groups on the peripheral around the macrocycle. These structural designs focus on increasing both the inner-sphere relaxivity and the second-sphere relaxivity while retaining the water exchange rate in the optimal window.

**Fig. 2:**
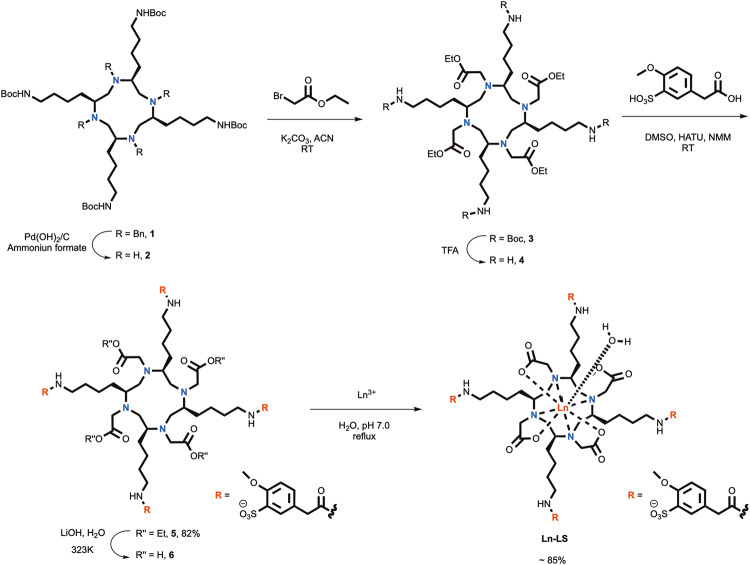
Synthesis of Ln-LS. Ln represents Eu(III) and Gd(III); counter ions are not shown for clarity.

As shown in Fig. 2, the chiral cyclen, compound **1**, is synthesized according to our previously reported method^7^. The aromatic molecule with SO_3_^−^ group is conjugated onto the amino groups after the substitution with four carboxylates (compound **4**, Fig. 2). In this way, the purification is simplified. Only two steps of purification are required by HPLC.

**Fig. 3:**
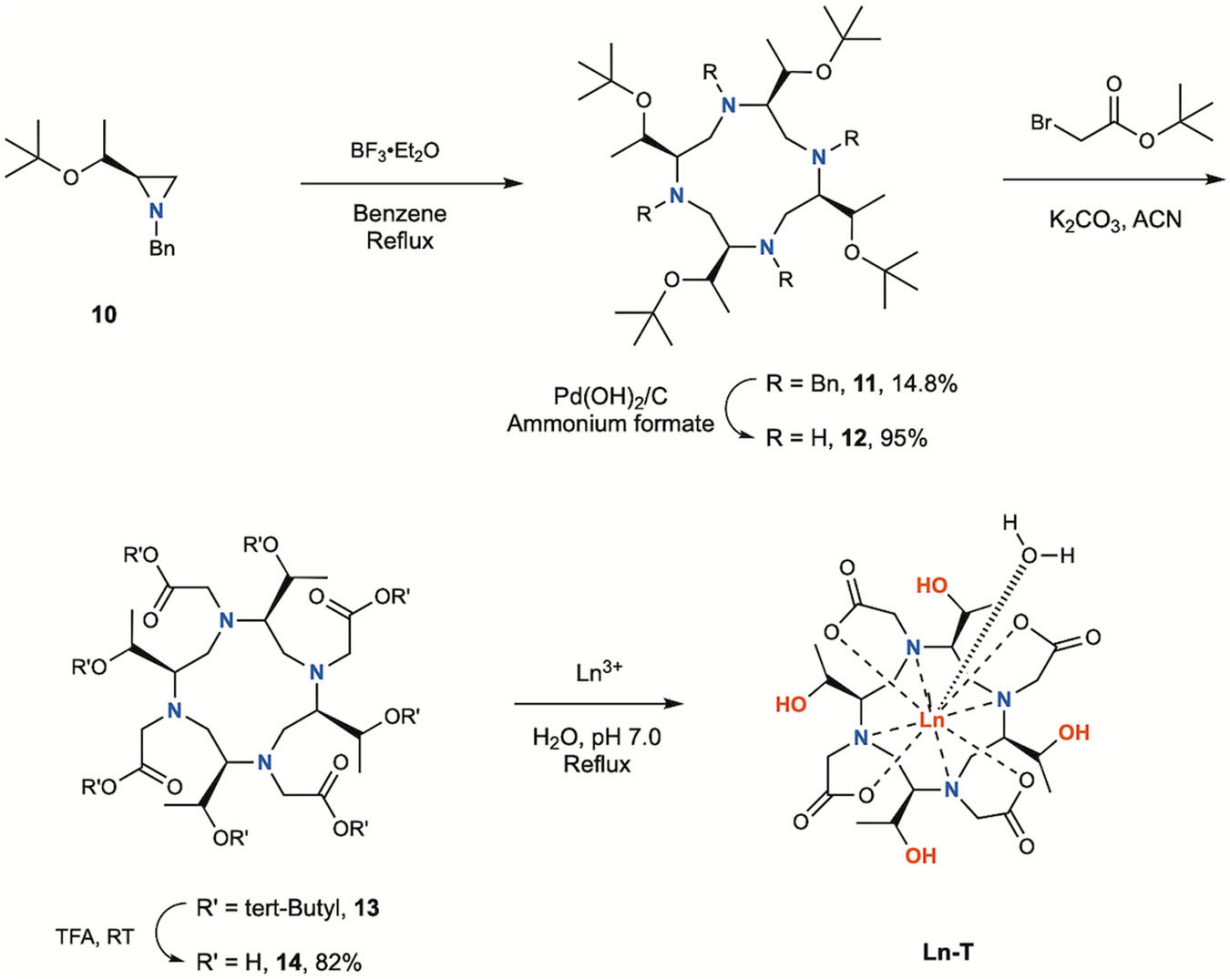
Synthesis of Ln-T. Ln represents Eu(III), Yb(III) and Gd(III); counter ions are not shown for clarity.

Similar to our standard chiral cyclen synthesis^7^, the complex Ln-T was first synthesized from the chiral aziridine compound **10** through the synthetic route in Supplementary Fig. S1, followed by a cyclization reaction to obtain the macrocyclic compound **11** (Fig. 3). After deprotection by using Pd(OH)_2_/C and ammonium formate, compound **12** was reacted with tert-butyl 2-bromoacetate in the presence of potassium carbonate to obtain compound **13**, which was then fully deprotected to obtain the final ligand **14**. Complexation was performed under neutral pH with a slight excess of lanthanide salts. The pH of resulting complex solution was adjusted to basic condition, excess lanthanide ions were then removed in the form of insoluble metal hydroxide by filtration, followed by the purification of reverse-phase HPLC^13^. The solubility of Gd-LS and Gd-T in water was also examine, Gd-LS showed moderate water solubility (31 mM) due to the relatively longer and bulkier chiral substituents, while Gd-T gave better water solubility (195 mM) due to the shorter and more hydrophilic functional groups.

### Identification of geometric isomers

**Fig. 4:**
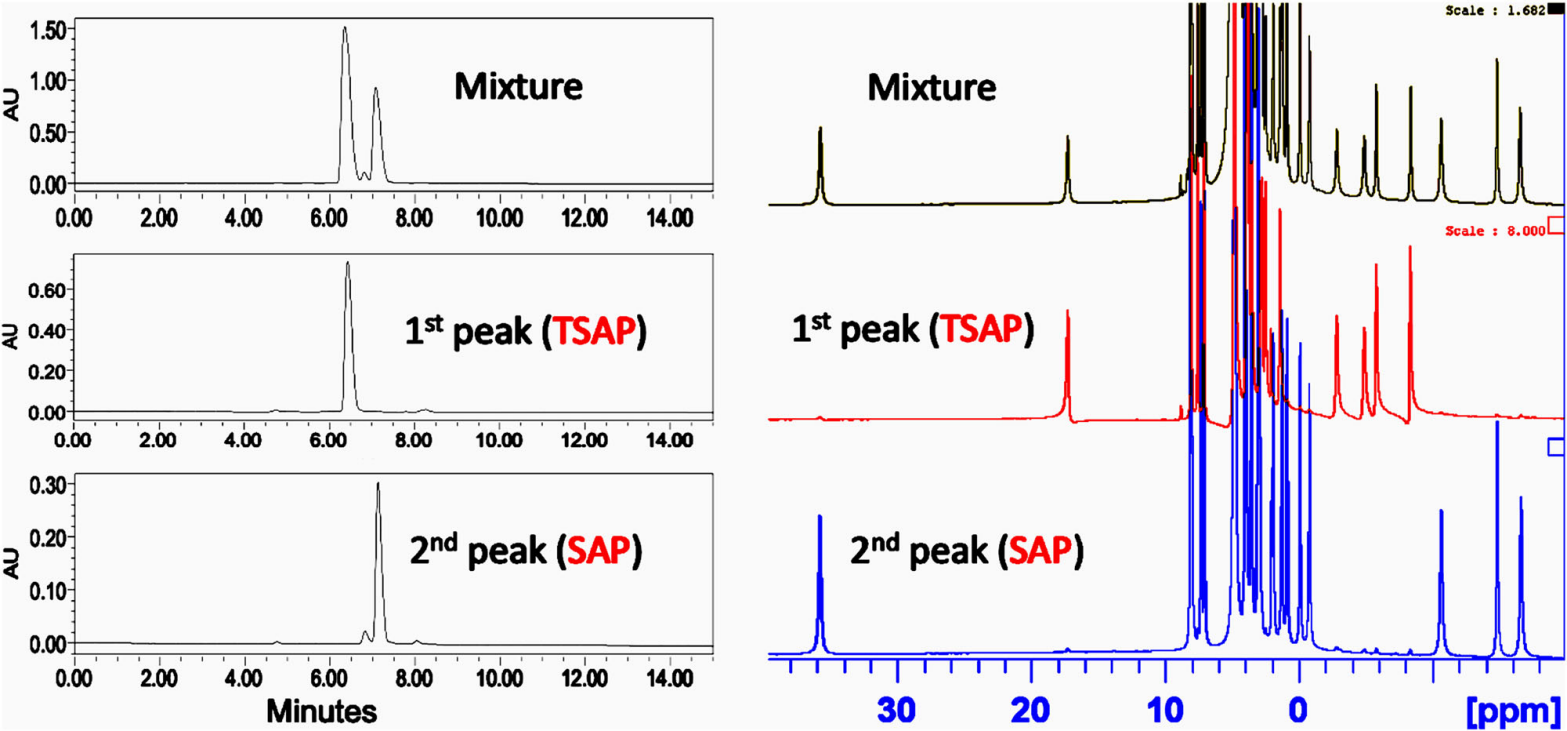
HPLC traces of Gd-LS (left) and ^1^H NMR spectrum of Eu-LS in D_2_O(right). Mixture before isolation (top); 1st peak after isolation (middle); 2nd peak after isolation (bottom).

**Fig. 5:**
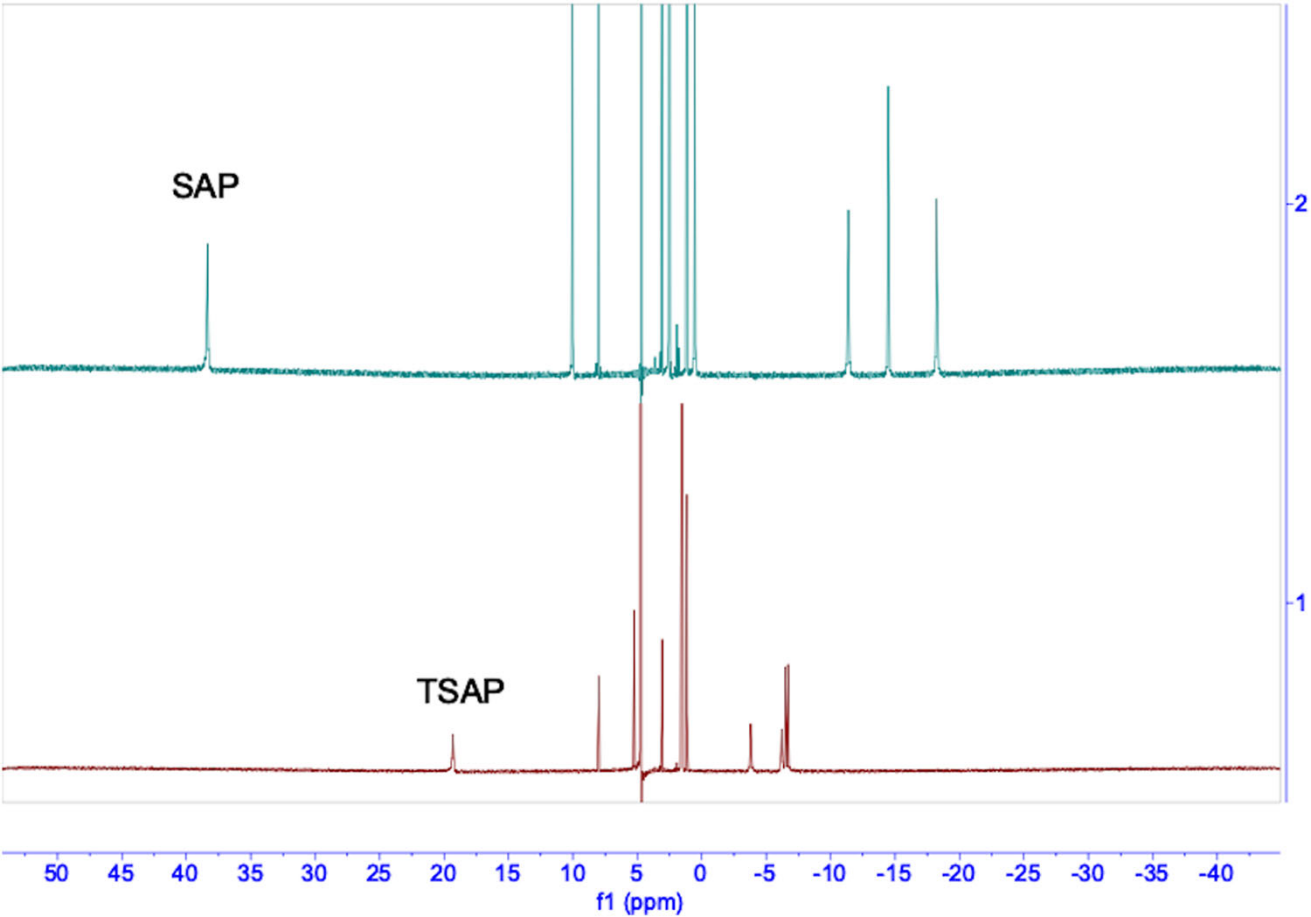
^1^H NMR spectra of Eu-T in D_2_O, 298 K. SAP isomer of Eu-T (top); TSAP isomer of Eu-T (bottom).

The coordination geometry of DOTA-based MRI contrast agents is well documented in literature^14^. For Gd-DOTA, there are four interconvertible geometric isomers existing in solution^14^. While for the chiral DOTAs, there are two nonconvertible isomers in solution. Depending on the chiral group, the formation of a single isomer is feasible^7^. To analyze the isomeric species of synthesized compounds, we first used HPLC to analyze the complexes of Gd-LS and Eu-LS, followed by NMR studies. It was not surprising that there were mainly two peaks in the HPLC traces (Fig. 4 & Supplementary Fig. S2). ^1^H NMR of the two isolated Eu-LS peaks represented two distinct coordination geometries. However, to our surprise, the retention sequence was different from that of our previously reported chiral DOTA complexes^7,15^. For Ln-LS, the retention order was TSAP isomer first, followed by SAP isomer in reverse-phase HPLC. We hypothesize that the difference in retention time might be affected by the four negatively charged SO_3_^−^ groups in Ln-LS. The concentration effect also influenced the retention time: the TSAP isomer of Gd-LS was observed as a shoulder peak at higher concentrations, while only one single peak was found at lower concentrations (as shown in Supplementary Fig. S3). ^1^H NMR studies were also conducted on Ln-T (Fig. 5). The HPLC trace showed that the SAP isomer was the major isomer (~86.7%) found in the solution states of Gd-T (Supplementary Fig. S7). We assume that the formation of geometric isomers might be affected by the changes in solvent interactions caused by the polar hydroxyl groups compared to the non-polar alkyl groups.

**Fig. 6:**
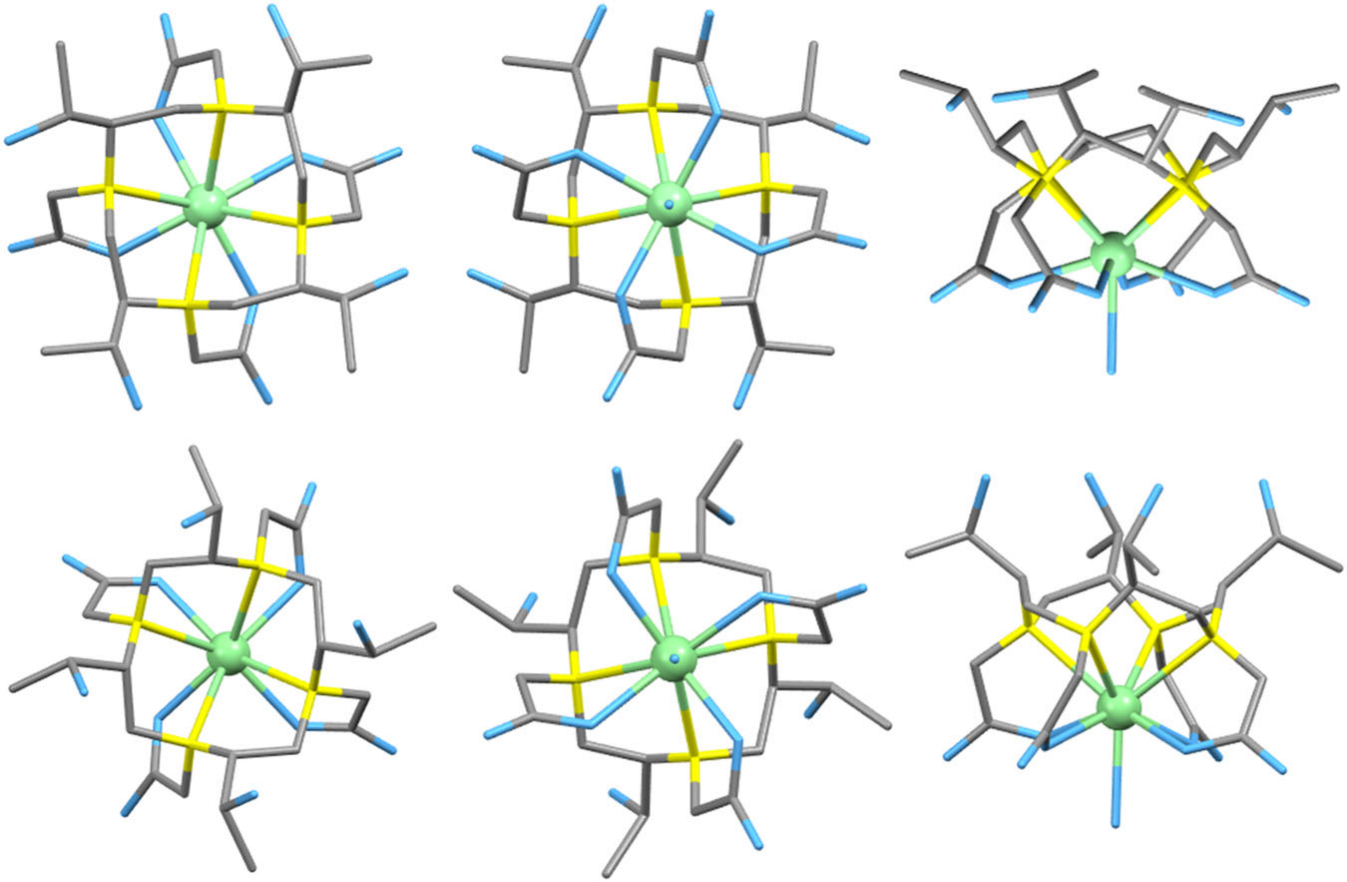
Crystal structures of Eu-T. SAP/corner isomer, from the top view, bottom view and side view (top row, from left to right); TSAP/side isomer, from the top view, bottom view and side view (bottom row, from left to right). Eu (green), N (yellow), O (blue), C (gray) and H (omitted).

To further evaluate the coordination geometry of our complexes Ln-LS and Ln-T, we attempted to obtain crystal structures for both, but only the latter was successful. The single crystal of Eu-T for single X-ray diffraction was grown by slow evaporation of aqueous solution (Fig. 6). The existence of both coordination isomers was observed, these isomers are SAP/corner and TSAP/side isomers, and they are typical for such chiral systems as shown in our previous work^7^. By comparing the average Eu-N bond length between the SAP (2.673 Å) and TSAP (2.728 Å) isomers of Eu-T, it is observed that in general, the Eu-N distance in TSAP is ~0.05 Å longer than that of the SAP. Uzal-Varela et al. recent study has also showed consistent funding in the Mn-DOTA system^16^. Although the difference is slight, we believe that this plays an important role in the thermodynamic stability. We expect that the SAP isomer may have higher thermodynamic stability than the TSAP isomer due to the shorter and hence stronger Eu-N bonds. Thus, accounting for the SAP isomer being the major product in the complexation. This is further verified by results obtained from both the NMR and HPLC studies showing the SAP as the major isomer. Single crystals from X-ray diffraction studies also confirmed the existence of the two geometric isomers.

It is well known that there are huge differences in the water-exchange rates between TSAP and SAP isomers in DOTA-based lanthanide complexes. The TSAP isomer usually has a much faster water-exchange rate. Hence, it is beneficial to use TSAP isomers as *T*_1_-enhanced MRI contrast agents; while SAP isomers, which in comparison, are slower in water-exchange, are more ideal as chemical exchange saturation transfer (CEST) MRI contrast agents^12^. The advantage of our system is that our isomers can be easily isolated by HPLC with normal achiral C18 column without interconversion^17,18^. Hence, the major SAP product in **Ln-T** can be further investigated as a pseudocontact shift tag for proteins as well as for circularly polarized luminescence (CPL) applications.

### Relaxivity measurements

Relaxivity measurements were also conducted to examine the properties of these Gd(III) complexes as MRI contrast agents. Owing to a trace amount of TSAP isomer formed in Gd-T, only the SAP isomer was tested. As shown in Table 1, the relaxivities of Gd-LS (TSAP and SAP isomers) and Gd-T were measured at 1.4 T, 37 °C. Both showed higher relaxivity than Gd-DOTA. In water, the *r*_1_ of (TSAP)Gd-LS and (SAP)Gd-LS was determined to be 7.4 mM^−1^s^−1^ and 14.5 mM^−1^s^−1^, respectively; whereas in the presence of 4.5% of HSA, the values increased to 12.4 mM^−1^s^−1^ and 17.5 mM^−1^s^−1^, respectively. This is somehow unexpected as the TSAP isomers of DOTA derivatives usually have higher relaxivity values than the SAP ones^7^. We hypothesized that the long chiral substituents on the macrocyclic backbone of Gd-LS might influence the relaxivity properties of its SAP and TSAP isomers causing it to differ from the parent achiral DOTA-like complexes. According to the Solomon-Bloembergen-Morgan (SBM) theory, the predicted optimal residence time of water co-ligand at 310 K (*τ*_M_^310K^) is in the range of 10 ns–30 ns, the SAP isomer may fit better in the optimal window. Our earlier studies on the influences of chiral groups have also shown that with the chiral DOTA design, the exchange rate of the SAP isomer is generally in the optimal range^7^. For Gd-T, the *r*_1_ in water and in the presence of 4.5% HSA was 5.2 mM^−1^s^−1^ and 5.7 mM^−1^s^−1^, respectively. These *r*_1_ values are much higher than Gd-DOTA and other Gd(III) chiral DOTA complexes with similar molecular weights under the same conditions^7^. This implies that a slight structural change, where by adding hydroxyl groups to the macrocyclic scaffold has improved the relaxivity by around 60% (+ 2 mM^−1^s^−1^) compared to Gd-DOTA. From the ^17^O NMR studies, the characteristic “slow exchange” profile corresponding to the 1/*τ*_m_ (negative slope at low temperature) of the plots ln(1/T_2r_) versus 1000/T could not be observed. This indicates that both geometric isomers of Gd-LS and Gd-T are “very fast exchange” systems, which may contribute to the enhancement on their overall relaxivity (Fig. S58 & S59)^1,19–21^.

**Table 1:** Relaxivities of Gd(III) complexes^a^.

Complex	r_1_ in H_2_O	r_2_ in H_2_O	r_1_ in 4.5% HSA	r_2_ in 4.5% HSA
(TSAP)Gd-LS	7.4 (0.1)	8.4 (0.1)	12.4 (0.8)	16.3 (1.5)
(SAP)Gd-LS	14.5 (0.3)	20.0 (0.2)	17.5 (1.0)	51.9 (2.8)
Gd-T	5.2 (0.2)	6.1 (0.2)	5.7 (0.4)	7.5 (0.6)
Gd-DOTA^b^	3.2	3.2	4.1	4.8

^a^Units of *r*_1_ and *r*_2_ are mM^−1^s^−1^, measured under 1.4 T, 37 °C, pH 7.

^b^Data recorded in ref. ^7^. SD is shown in ().

We postulate that the improvement in relaxivity is caused by the enhanced contribution from the second-sphere and outer-sphere relaxivity of both Gd-LS and Gd-T. Similar phenomenon was observed from Morrow’s group work on a Fe(III) macrocyclic MRI contrast agents, where by appending sulfonate or hydroxyl groups to the macrocyclic ring, the contribution from the second-sphere on the overall complex relaxivity could be enlarged and lead to a significant enhancement in relaxivity^22–24^. It is assumed that the water exchanges with the surrounding bulk water molecules could be promoted as the distances between the Gd(III) metal center and the water molecules are shortened due to the formation of hydrogen bonds with either sulfonate or hydroxyl group. The water-accessible surface area are also enhanced because of these hydrophilic functional groups and optimized the second-sphere hydration, subsequently increasing the water-exchange rates by lowering the activation energy of the exchange process^25^. Due to the existence of pH-sensitive hydroxyl and sulfonate groups in Gd-T and Gd-LS, the contribution in high relaxivity might also come from the proton exchange processes rather than the second-sphere or outer-sphere water exchange. Hence, to further investigate the origin of enhanced relaxivity for Gd-LS and Gd-T, the pH dependence of *r*_1_ relaxivity measurements were performed as shown in Supplementary Fig. S25. For both complexes, their pH profiles are similar, no significant enhancements were observed in either acidic or basic conditions, indicating no catalyzed proton exchange occurred. Their profiles for *r*_1_ relaxivity are fairly flat over pH 2 to 11, suggesting the crucial roles of the second-sphere and outer-sphere water exchange in contributing to the high relaxivity^26^. In addition, the complex stability in the above mentioned pH range is evidence of no demetallation, as this would lead to obvious changes in the relaxivity.

### Kinetic and potentiometric studies

**Fig. 7:**
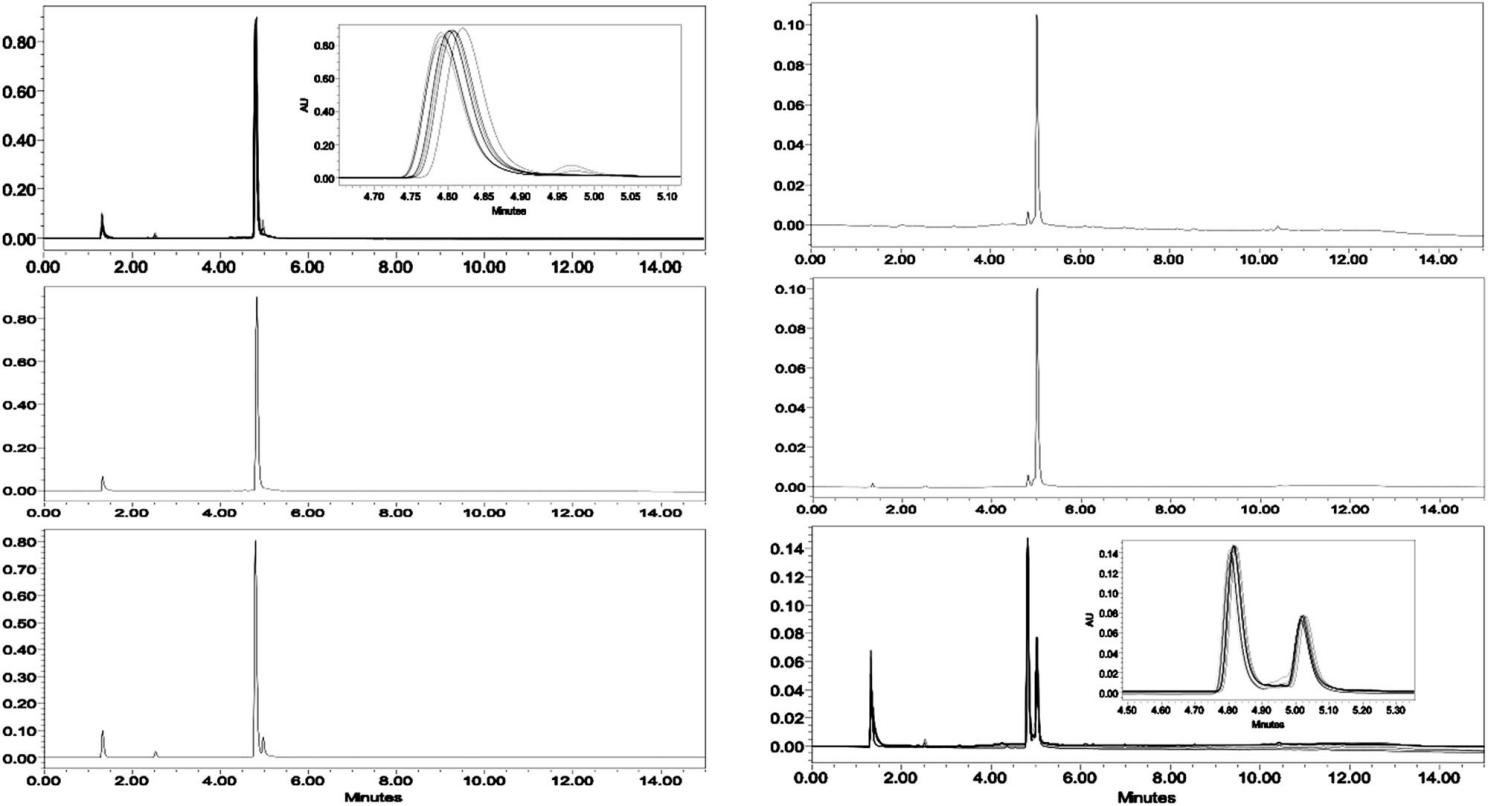
Assessment of kinetic inertness of Gd-LS showing different HPLC traces in 1 N HCl condition. A merged graph of (TSAP)Gd-LS at RT over 158 h, zoom-in graph showing the peak heights inserted (top, left); (TSAP)Gd-LS, freshly prepared solution (middle, left); (TSAP)Gd-LS, stayed at RT for 158 h (bottom, left); (SAP)Gd-LS, freshly prepared solution (top, right); (SAP)Gd-LS, stayed at RT for 158 h (middle, right); A merged graph of Gd-LS (mixture) at RT over 158 h, zoom-in graph showing the peak heights inserted (bottom, right).

In order to examine the kinetic inertness of Gd-LS and Gd-T, the compounds were further examined under extreme conditions to check if any demetallation occurred. Demetallation of Gd(III) complexes is a serious concern as free Gd(III) metal ions are toxic. Thus, inertness is regarded as one of the most important parameters for the screening of GBCAs. Gd-DOTA is one of the most stable complexes among the clinically used GBCAs, and it is used as a benchmark in our analysis. We found that its half-lifetime in 1 N of HCl at room temperature was around 13 h when tested by HPLC^7^. In this study, we tested the kinetic inertness of Gd-LS and Gd-T in both 1 N HCl and 10 eq. of Zn^2+^ by HPLC and *T*_1_ relaxivity measurements as shown in Supplementary Fig. S8 where no obvious decomplexation was observed after monitoring for at least 100 h under these conditions. Figure 7 shows the HPLC traces of Gd-LS at various time points, again no obvious decomplexation was observed, and only an insignificant shoulder peak formed after 158 h, which might be due to the presence of (SAP)Gd-LS. Under the same conditions, (SAP)Gd-LS also showed a 5% shoulder peak of (TSAP)Gd-LS. We hypothesis that the observation of these trace isomeric residues are more prominent at extreme acidic conditions due to the protonation of the SO_3_^−^ groups. Upon investigating Gd-LS (as a mixture of both isomers), it was observed that both isomers were also stable even after 158 h. The stabilities of Gd-DOTA, Gd-LS, and Gd-T in 1 N HCl were further studied by measuring the changes in their relaxivity at 37 °C. Supplementary Fig. S8 showed the half-lifetime of Gd-DOTA in 1 N HCl at 37 °C was around 13 h according to the integrated area of the corresponding HPLC peak, while Gd-LS and Gd-T had no obvious decomplexation under the same conditions for at least 4 days.

In addition, to further evaluate the stabilities of Gd(III) complexes, the protonation constants of ligands T and L (L the intermediate of LS, was used as the study of LS was not possible due to the multiple SO_3_^−^ groups) were determined by pH-potentiometric titration under 1.0 M ionic strength at 25 °C^27,28^. NMe_4_Cl was used as the organic electrolyte to avoid the interactions between the macrocyclic ligands and the inorganic cations, such as K^+^ in KCl^29^. The potentiometric data were refined by *HYPERQUAD 2013*^30^. According to the results in Table 2, both ligands **T** and **L** gave larger summation of protonation constants (∑logK_i_^H^ ≈ 38) compared to that of DOTA. In addition, the stability constant of Gd-T (log K_GdL_ = 30.01) is also larger than that of Gd-DOTA (log K_GdL_ = 25.58)^31^. This elucidates that the Gd complexes of ligand T should have better stabilities than Gd-DOTA^32^.

**Table 2:** Protonation constants and stability constants of Ligand-T, Ligand-L and DOTA^18^.

log K_i_^H^	Ligand-T^a^	Ligand-L^a^	DOTA^b^
log K_1_^H^	11.30 (0.07)	13.53 (0.25)	11.14
log K_2_^H^	11.10 (0.09)	10.25 (0.12)	9.69
log K_3_^H^	10.30 (0.28)	9.90 (0.20)	4.85
log K_4_^H^	6.08 (0.30)	4.63 (0.19)	3.95
∑log K_i_^H^	38.78	38.31	29.62
log K_GdL_	30.01^b^	/	25.58^(32)^

^a^I = 1.0 M NMe_4_Cl, at 25 °C.

^b^I = 1.0 M KCl, at 25 °C, where K_i_^H^ = [H_i_L]/[H_i-1_L][H^+^], SD were shown in brackets.

^b^I = 0.1 M NMe_4_Cl, at 25 °C^31^.

### Biodistribution studies

**Fig. 8:**
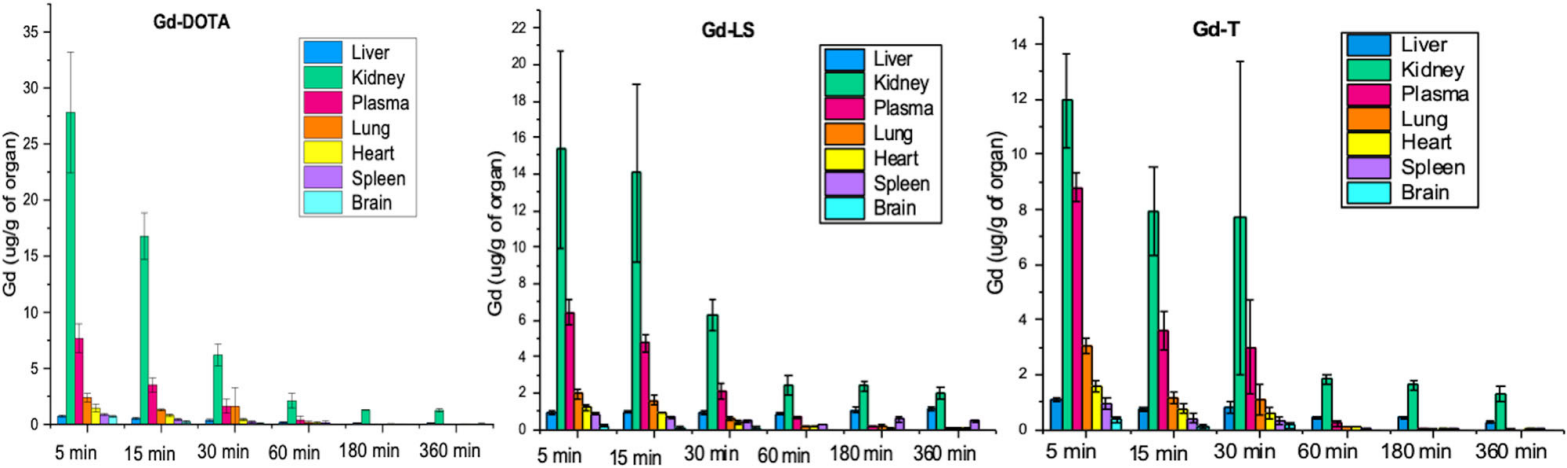
Biodistribution results of Gd-DOTA (left), Gd-LS (middle) and Gd-T (right) in mice. The error bars represent the standard deviation.

Due to their higher stability and superior relaxivity over Gd-DOTA, Gd-LS (mixture of both isomer) and (SAP)Gd-T were further selected for biodistribution studies. Biodistribution studies were performed in standard BALB/c mice, and the Gd concentrations in the organs of mice were analysed by ICP-MS at 5, 15, 30, 60, 180, and 360 min after injection. Gd-LS and Gd-T showed similar distribution profiles to Gd-DOTA (Fig. 8). However, a higher population of Gd metal content was observed in the kidney.

Compared to Gd-DOTA, Gd-LS and Gd-T showed longer retention time in the first 30 min. This is due to the longer retention time in plasma. For Gd-LS, the result is consistent with the result of the relaxivity study, which showed enhancement in the presence of HSA protein. The π-π interactions between the aromatic arms in Gd-LS and HSA proteins can enhance relaxivity and thus prolonged the retention time. Gd-T also showed comparable retention time as Gd-LS, however, it did not exhibit significant HSA protein interactions in the relaxivity study shown in Table 1. One possible explanation could be the difference in molecular weight of these two Gd complexes. The retention time in the plasma may also be affected by the size of molecule. Although Gd-LS showed interactions with HSA while Gd-T did not, the size of Gd-LS is almost triple to Gd-T, which has a much smaller molecular size. In addition, the actual physiological conditions in biodistribution studies are not reflected by the benchtop relaxivity studies. Factors other than the HSA protein interactions might also influence the retention time in the plasma.

### MRI Phantom scans

**Fig. 9:**
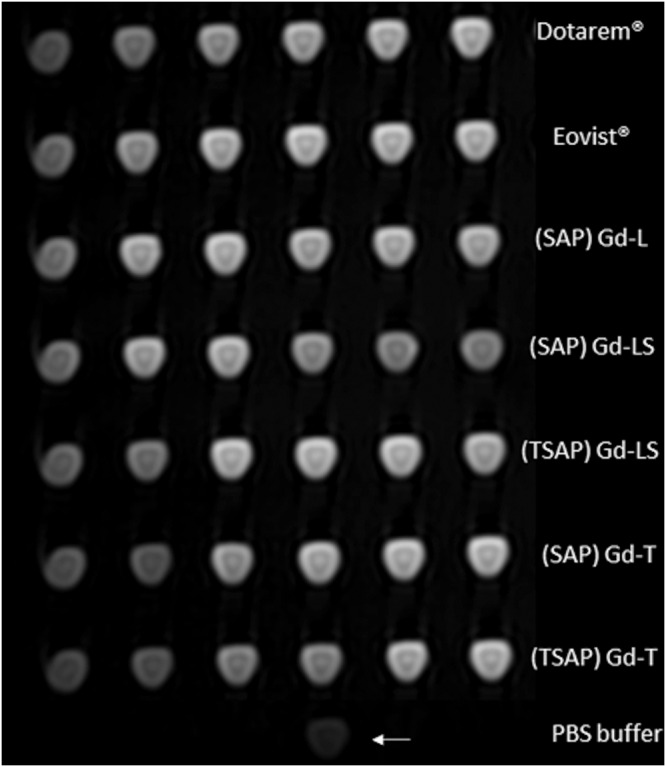
MRI phantom images of Gd complexes. Captured image with increasing concentrations from left to right (0.1–1.3 mM, in 1X Gibco^TM^ PBS buffer, pH 7.4). Dotarem®, Eovist®, (SAP)Gd-L, (SAP)Gd-LS, (TSAP)Gd-LS, (SAP)Gd-LS, (SAP)Gd-T, (TSAP)Gd-T and PBS buffer were placed from top to bottom accordingly.

**Fig. 10:**
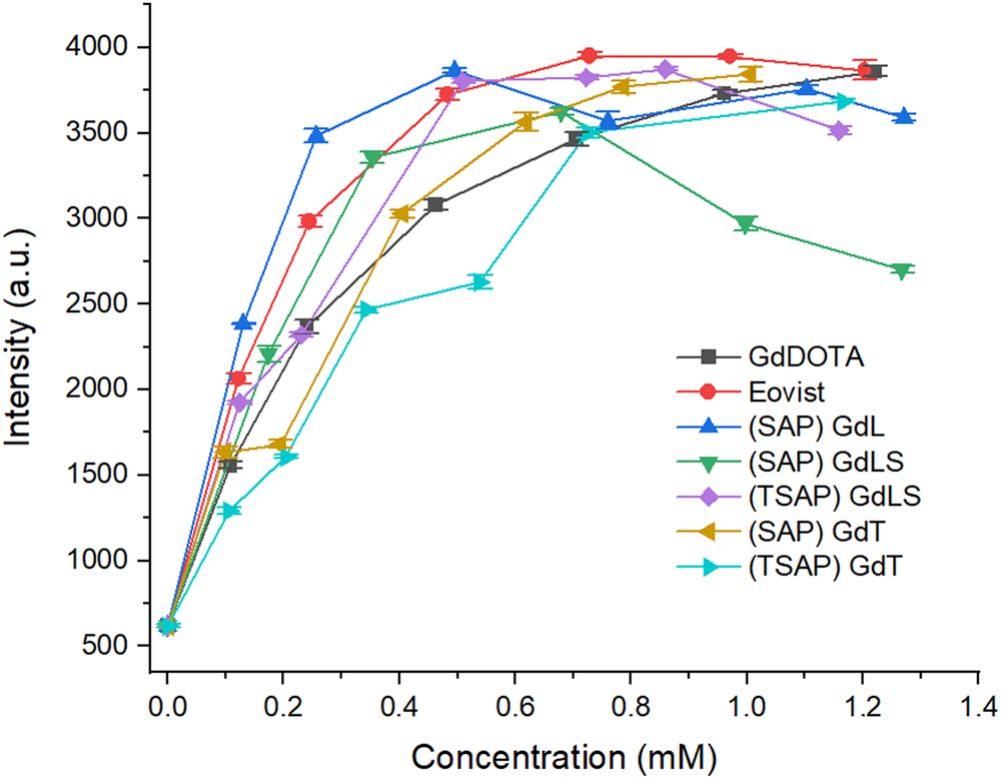
Signal intensity of MRI phantom images of various Gd complexes with different concentrations. Concentrations were obtained from ICP-MS studies, error bars represent the SD.

To further mimic the practical uses of GBCAs, MRI phantom scans were also conducted with Dotarem® (Gd-DOTA), Eovist® (Gd-EOB-DTPA), Gd-L, (TSAP)Gd-LS, (SAP)Gd-LS, (TSAP)Gd-T and (SAP)Gd-T with different concentrations ranging from 0 to 1.3 mM in a clinical MR scanner (GE Signa Premier 3.0 T, GE healthcare, Milwaukee, WI, USA). The phantoms were scanned using SPGR (Spoiled Gradient Recalled) pulse sequence with parameters of flip angle = 55, repetition time = 20 ms, echo time = 2.1 ms, EC = 1/1 31.2 KHz^33^. In clinical applications, SPGR shows promising results in volumetric, breath hold, and dynamic imaging. The phantom images are shown in Fig. 9. The signal intensities of the above-mentioned gadolinium complexes are compared in Fig. 10.

The MR signal intensities of Gd-L, Gd-LS and Gd-T complexes enhanced with increasing concentration up to around 0.7 mM, then plateaued. The results are consistent with reported values that the concentration of a contrast agent has no linear correlation with the signal intensity of its MR image among the whole concentration range, but only partially correlated at lower concentrations^34,35^. In this phantom scan, Gd-L, Gd-LS, and Gd-T all gave competitive results compared to the commercially available contrast agents, Dotarem® and Eovist®. In general, all chiral compounds, except (TSAP)Gd-T, displayed higher signal intensities than Dotarem® at most of the concentrations tested in Fig. 10. These results are in agreement with the relaxivity measurements (Table 1). As shown in Fig. 10, the macrocyclic complexes, (SAP)Gd-L, (TSAP)Gd-LS and (SAP)Gd-T, also provided similar contrast as the linear compound Eovist®, offering a potential competitive macrocyclic CA with comparable intensity at the same dosage and higher stability as macrocyclic complexes are known to have higher stability than the linear ones. This can further lower the chances in decomplexation which causes metal toxicity^26^. Notably, significant signal enhancement from (TSAP)Gd-LS was observed at lower concentrations (≤1 mM) when compared to Dotarem®, which makes it the most promising candidate for further development, as the contrast agent can be used at lower dosage, reducing potential side effects.

## Conclusion

In summary, two new chiral DOTAs with their lanthanide complexes were synthesized and characterized where possible by NMR, HPLC and X-ray single crystallography. Gd-LS and Gd-T was shown to display higher stability and relaxivity than Gd-DOTA, rendering them as potential CAs in terms of safety and efficiency. Both coordination geometric isomers of (TSAP)Gd-LS, (SAP)Gd-LS and (SAP)Gd-T possess prominent relaxivity compared to Gd-DOTA. In contrast to the general TSAP/SAP Gd macrocyclic complexes, the SAP geometric isomer of Gd-LS gave a higher *r*_1_ relaxivity than the TSAP one. This indicates the (SAP)Gd-LS may lay in a more optimal window of water-exchange rate described by the SBM theory. Hence, this finding reflects the importance of the control in coordination isomer formation and also the use of specific isomers in both chiral and achiral DOTA complexes for particular applications. Gd-T and Gd-LS also display the importance of the secondary-sphere contributions caused by the hydroxyl and sulfonate groups. This further proves that even minor modification to structural designs can have an important influence to the overall relaxivity. As a result, the second-sphere and outer-sphere relaxation should be considered as a significant parameter in the developing and designing of new contrast agents. The substituent of the chiral functional group reported in this work show an alternative way to introduce such effects without modifying the pendant arms of the macrocyclic compounds whilst maintaining or even enhancing the stability. The biodistribution profiles of Gd-LS and Gd-T as well as the MRI phantom studies also further demonstrate the potential practicality and use of these compounds as contrast agents. Further studies of this series of gadolinium contrast agents will be conducted in our future work to understand their pharmacokinetic properties.

## Methods

### General

Unless otherwise noted, all chemicals were of reagent-grade and were purchased from Sigma-Aldrich or Acros Organics and used without further purification. Concentrated Nitric Acid (Trace-metal Grade 70% Anaqua Cat#AN-308A-2500) was purchased from Anaqua. Gadolinium standard (1.005 mg L^−1^ Cat#2017136-100 ML) and Indium standard (1000 mg L^−1^ ± 2 mg L^−1^ Cat#00734-100 ML) were obtained from Sigma-Aldrich. ^1^H and ^13^C NMR spectra were recorded on a Bruker Ultrashield 400 Plus NMR spectrometer (at 400 MHz and 100 MHz, respectively). High-resolution mass spectrometry was performed on an Agilent 1260 Infinity Series apparatus with Agilent 6540 UHD Accurate-Mass Q-TOF LC/MS detection in the range of 100–3200, while the low-resolution mass spectrometry was obtained on a Micromass Q-TOF 2 mass spectrometer detection in the range of 100–2000.

### HPLC

Reverse-phase semi-preparative purification was performed on the Waters UPLC system with UV detection from 220 to 330 nm using a Waters T3 Column (250 × 19 mm). Two methods were used: Method A: mobile phase A was water with 0.05% TFA, mobile phase B was acetonitrile. Method B: the mobile phase A was water with 10 mM ammonium formate added; mobile phase B was 90% acetonitrile/10% water with 10 mM ammonium formate added, the flow rate was 8 mL per min.

The analytical HPLC was performed on (Method A) Waters HPLC system with UV detection from 220 to 350 nm. Column: XBridge Shield RP 18, 2.5 *μ*m, 2.1 × 50 mm. Mobile phase A: H_2_O with 0.05% TFA; mobile phase B: acetonitrile. Gradient: starting from 95% A/5% B, the fraction of B increased to 60% over 8 mins, then re-equilibrated at 5% B for 4 min, 0.2 mL per min. (Method B) SHIMADZU HPLC system with UV detection at 230 nm, and fluorescence detection at 312 nm with excitation at 254 nm. Column: Shim-pack GIST C18 5 *μ*m, 4.6 I.D. X 150 nm. Mobile phase A: H_2_O with 10 mM ammonium formate; mobile phase B: acetonitrile. Isocratic: 98% A/2% B, 1 mL per min.

### Potentiometric measurement

The protonation constants of ligands were determined by potentiometric titrations using Metrohm 905 Titrando. The titration data was processed by the *Hyperquad2013*^30^. All experiments were performed at 25 °C and using NMe_4_Cl as the electrolyte. 1 N HCl, 0.1 M KOH, double distillated water and argon protection were used during the titration. Equilibration times were 90 s for ligand only titrations^36^.

### Determination of thermodynamic stability constant

“Out-of-cell” experiment (batch method) was performed to measure the stability constant of Gd-T^37–39^. The free ligand-T was dissolved in 0.1 M NMe_4_Cl solution with double distillated water in the concentration of 2.08 mM. GdCl_3_·6H_2_O was added to the solution of ligand T in 1.94 mM. 26 mL of solution mixture of Gd^3+^ and ligand **T** was prepared and divided into 16 individua solutions (1.5 mL each). The pH of each vials was adjusted with HCl and KOH in the range of 2–11. The sealed vials were incubated at 60 °C for 1 week. The pH of each vials was then recorded at ambient temperature by Mettler Toledo FiveGo F2 pH meter. The titration data was fitted by *Hyperquad2013* (Supplementary Fig. S26)^30^.

### Relaxivity measurement

Relaxivity measurements were performed on a Bruker mq60 Minispec (Germany) at 1.5 T and 37 °C. Longitudinal (*T*_1_) relaxation times were measured using an inversion recovery experiment with 10 inversion times of duration ranging between 0.05 × *T*_1_ and 5 × *T*_1_. Transverse (*T*_2_) relaxation times were measured using a CPMG pulse sequence. Relaxivity (*r*_1_, *r*_2_) was determined from the slope of a plot of 1/*T*_1,2_ vs [Gd] for 5 concentrations of Gd(III) samples with and without 4.5% HSA. Concentration of Gd complexes were determined by ICPMS on an Agilent 7900 Series system (USA), with 7 standards (0.5–50 ppb) of Gd in 2% of HNO_3_. The pH-dependence *r*_1_ relaxivity studies were measured in PBS buffer and pH was adjusted by 1 N HCl and 1 N NaOH solution.

### Photophysical measurement

All the photophysical measurements are conducted in triplicates (Supplementary Table S1 & Fig. S49–S56). All the solution-state measurements were conducted using Type 23 quartz curettes with 10 mm path length from Starna Scientific (London, UK). Time-decay measurements of Eu-LS and Eu-T were performed in H_2_O and D_2_O, respectively^40,41^. Mini-tau lifetime spectrometer (Edinburgh Instruments, UK) with laser excited at 405 nm was used. Bandpass filter (575–625 nm) was applied to collect the europium emission from samples.

### Variable-Temperature ^17^O NMR Measurements

^17^O NMR measurements of Gd samples were conducted at 14.1 T on 0.5 mL 5% H_2_^17^O enriched sample solutions contained in 5 mm o.d. tubes on Bruker Advance-III (600 MHz FT-NMR system). ^17^O transverse relaxation times of distilled water (pH 6.5–7) and Gd samples were measured using a CPMG sequence. The concentration of the samples was ~10 mM. The data (Supplementary Fig. S57–59) are calculated as the reduced transverse relaxation rate according to the equation: 1/*T*_2r_ = 55.55/([Gd complex]*qT*_2p_), where the concentration of Gd complexes was confirmed by ICP-MS; the *q* value is the number of coordinated water molecules and is obtained from the lifetime measurements of the corresponding Eu complexes in H_2_O and D_2_O, respectively; calculated according to Parker’s and Horrocks’ equations (shown in SI); T_2p_ is the paramagnetic transverse relaxation rate^40–43^.

### MRI phantom scan

Clinical MRI phantom scan was conducted on the SIGNA^TM^ Premier 3.0 T MR scanner (GE, Boston, MA, USA) in SPGR sequence for T_1_ relaxivity with flip angle = 55, TR = 120 ms, TE = 2.1 ms, and EC = 1/1 31.2 kHz. Gadolinium complexes were serially diluted with phosphate-buffered saline (PBS) to concentrations of 0 to 1.3 mM and stored in 0.6 mL centrifuge tubes for scanning. The complex concentrations were double-confirmed before and after the phantom scan by ICPMS study.

### Biodistribution study

Eight-week-old male BALB/c mice each weighing approximately 25 g were purchased from BioLASCO (Taiwan). BALB/c mice were administered with a single dose of 0.025 mmol kg^−1^ compound (Gd-LS, Gd-T, or Gd-DOTA; 3 mice per compound per time point) in a 100 μl volume via lateral tail vein injection. Mice were euthanized and their blood collected by cardiac puncture at each time point (5, 15, 30, 60, 180, or 360 min post-injection). A control group of 4 mice was treated with saline and killed at time point 0. The liver, lung, heart, kidneys, spleen, and brain were removed from the animals and weighed before analysis. All animal experiments were approved by the Hong Kong Polytechnic University Animal Subjects Ethics Subcommittee and conducted in accordance with the Institutional Guidelines and Animal Ordinance of the Department of Health.

Gd content in tissue and blood samples was quantified via inductively coupled plasma–mass spectrometry (ICP-MS) analysis using the Agilent 7500ce (Agilent Technologies, CA, USA). 10 ml Gd standard solution (Blank, 0.5, 1, 2, 5, 10, 20, 50 ppb) was prepared by diluting 1000 ppm Gd standard solution with 1% nitric acid. 100 µl of plasma or approximately 0.1–0.2 g organ tissue was digested with 200 µl concentrated nitric acid in a 70 °C water bath for 3 h. Samples were then further diluted to a volume of 10 ml with deionized water. 500 µl of diluted sample solution was then taken and added to 9450 µl 1% nitric acid. 50 µL internal standard (1 ppm indium [In]) was spiked into each sample. Gd concentration in diluted samples was calculated using the internal standard calibration curve (Gd/In intensity to Gd concentration). The Gd concentration in organs was calculated using the following formula: (Gd concentration (μg)×Dilution factor)/(tissue weight (g)). Graphical analyses of biodistribution in the animal studies are expressed as the mean ± SD.

### Syntheses

Syntheses of compounds are shown in Supplementary Information and all the new compounds are fully characterized.

### Reporting summary

Further information on research design is available in the Nature Portfolio Reporting Summary linked to this article.

### Supplementary information

Supporting Information

Description of Additional Supplementary Files

Supplementary Data 1

Reporting Summary

## Data Availability

For more synthetic details see Supplementary Methods. For HPLC traces see Supplementary Figs. S2–S7. For Stability measurements see Supplementary Figs. S8–S18. For relaxivity measurements see Supplementary Figs. S19–S25. For batch titration see Supplementary Fig. S26. For NMR spectra see Supplementary Figs. S27–S44. For HRMS spectra see Supplementary Figs. S45–S48. For lifetime plots see Supplementary Figs. S49–S56. For ^17^O NMR studies see Supplementary Figs. S57–S59. The X-ray crystallographic coordinates for structures reported in this study have been deposited at the Cambridge Crystallographic Data Centre (CCDC), under deposition numbers CCDC No.: 1882241 for Eu-T, see Supplementary Data 1. These data can be obtained free of charge from The Cambridge Crystallographic Data Centre via www.ccdc.cam.ac.uk/data_request/cif.

## References

[1] Caravan P, Ellison JJ, McMurry TJ, Lauffer RB (1999). Gadolinium(III) Chelates as MRI Contrast Agents: Structure, Dynamics, and Applications. Chem. Rev..

[2] Wahsner J, Gale EM, Rodriguez-Rodriguez A, Caravan P (2019). Chemistry of MRI Contrast Agents: Current Challenges and New Frontiers. Chem. Rev..

[3] Perez-Rodriguez J, Lai S, Ehst BD, Fine DM, Bluemke DA (2009). Nephrogenic systemic fibrosis: incidence, associations, and effect of risk factor assessment-report of 33 cases. Radiology.

[4] McDonald RJ (2015). Intracranial Gadolinium Deposition after Contrast-enhanced MR Imaging. Radiology.

[5] Clough TJ, Jiang L, Wong KL, Long NJ (2019). Ligand design strategies to increase stability of gadolinium-based magnetic resonance imaging contrast agents. Nat. Commun..

[6] Chan M, Lux J, Nishimura T, Akiyoshi K, Almutairi A (2015). Long-Lasting and Efficient Tumor Imaging Using a High Relaxivity Polysaccharide Nanogel Magnetic Resonance Imaging Contrast Agent. Biomacromolecules.

[7] Dai L (2018). Chiral DOTA chelators as an improved platform for biomedical imaging and therapy applications. Nat. Commun..

[8] Waghorn PA (2017). Molecular magnetic resonance imaging of lung fibrogenesis with an oxyamine‐based probe. Angew. Chem. Int. Ed. Engl..

[9] Terreno E, Castelli DD, Viale A, Aime S (2010). Challenges for molecular magnetic resonance imaging. Chem. Rev..

[10] Zhang Z, Kolodziej AF, Greenfield MT, Caravan P (2011). Heteroditopic binding of magnetic resonance contrast agents for increased relaxivity. Angew. Chem. Int. Ed. Engl..

[11] Tei L (2015). Polyhydroxylated GdDTPA-derivatives as high relaxivity magnetic resonance imaging contrast agents. RSC Adv..

[12] Caravan P, Esteban-Gomez D, Rodriguez-Rodriguez A, Platas-Iglesias C (2019). Water exchange in lanthanide complexes for MRI applications. Lessons learned over the last 25 years. Dalton Trans..

[13] Z. Kovács, et al. *Contrast agents for MRI: Experimental methods*. Ch. 1, 33 (Royal Society of Chemistry, 2017).

[14] Aime S (1997). Conformational and Coordination Equilibria on DOTA Complexes of Lanthanide Metal Ions in Aqueous Solution Studied by1H-NMR Spectroscopy. Inorg. Chem..

[15] Dai L (2019). Synthesis of Water-Soluble Chiral DOTA Lanthanide Complexes with Predominantly Twisted Square Antiprism Isomers and Circularly Polarized Luminescence. Inorg. Chem..

[16] Uzal-Varela R (2022). Thermodynamic Stability of Mn(II) Complexes with Aminocarboxylate Ligands Analyzed Using Structural Descriptors. Inorg. Chem..

[17] Gobl C (2016). Increasing the Chemical‐Shift Dispersion of Unstructured Proteins with a Covalent Lanthanide Shift Reagent. Angew. Chem. Int. Ed. Engl..

[18] Dai L, Lo WS, Coates ID, Pal R, Law GL (2016). New Class of Bright and Highly Stable Chiral Cyclen Europium Complexes for Circularly Polarized Luminescence Applications. Inorg. Chem..

[19] Pubanz D, González G, Powell DH, Merbach AE (1995). Unexpectedly Large Change of Water Exchange Rate and Mechanism on [Ln(DTPA-BMA)(H2O)] Complexes along the Lanthanide(III) Series. Inorg. Chem..

[20] Caravan P (2007). Albumin Binding, Relaxivity, and Water Exchange Kinetics of the Diastereoisomers of MS-325, a Gadolinium(III)-Based Magnetic Resonance Angiography Contrast Agent. Inorg. Chem..

[21] Maigut J, Meier R, Zahl A, Eldik RV (2007). Elucidation of the solution structure and water-exchange mechanism of paramagnetic [Fe(II)(edta)(H(2)O)](2-). Inorg. Chem..

[22] Asik D (2020). Modulating the Properties of Fe(III) Macrocyclic MRI Contrast Agents by Appending Sulfonate or Hydroxyl Groups. Molecules.

[23] Xue SS (2022). Bioimaging agents based on redox-active transition metal complexes. Chem. Sci..

[24] Botta M (2000). Second Coordination Sphere Water Molecules and Relaxivity of Gadolinium(III) Complexes: Implications for MRI Contrast Agents. Eur. J. Inorg. Chem..

[25] Siriwardena-Mahanama BN, Allen MJ (2013). Strategies for optimizing water-exchange rates of lanthanide-based contrast agents for magnetic resonance imaging. Molecules.

[26] Kras EA, Abozeid SM, Eduardo W, Spernyak JA, Morrow JR (2021). Comparison of phosphonate, hydroxypropyl and carboxylate pendants in Fe(III) macrocyclic complexes as MRI contrast agents. J. Inorg. Biochem..

[27] Clarke ET, Martell AE (1991). Stabilities of trivalent metal ion complexes of the tetraacetate derivatives of 12-, 13- and 14-membered tetraazamacrocycles. Inorg. Chim. Acta.

[28] L. Burai, I. Fábián, R. Király, E. Szilágyi and E. Brücher, *J. Chem. Soc. Dalton Trans*. 243–248. 10.1039/A705158A (1998).

[29] Tircso G, Webber BC, Kucera BE, Young VG, Woods M (2011). Analysis of the conformational behavior and stability of the SAP and TSAP isomers of lanthanide(III) NB-DOTA-type chelates. Inorg. Chem..

[30] Gans P, Sabatini A, Vacca A (1996). Investigation of equilibria in solution. Determination of equilibrium constants with the HYPERQUAD suite of programs. Talanta.

[31] Moreau J (2003). Thermodynamic and Structural Properties of Eu3+, Gd3+and Tb3+Complexes with 1,4,7,10‐Tetra(2‐glutaryl)‐1,4,7,10‐tetraazacyclododecane in Solution: EXAFS, Luminescence, Potentiometric Studies, and Quantum Calculations. Eur. J. Inorg. Chem..

[32] Dai L (2021). Design of functional chiral cyclen-based radiometal chelators for theranostics. Inorg. Chem..

[33] Elster AD (1993). Gradient-echo MR imaging: techniques and acronyms. Radiology.

[34] Shahbazi-Gahrouei D, Williams M, Allen BJ (2001). In vitrostudy of relationship between signal intensity and gadolinium‐DTPA concentration at high magnetic field strength. Australas. Radiol..

[35] Nazarpoor M, Poureisa M, Daghighi MH (2012). Comparison of maximum signal intensity of contrast agent on t1-weighted images using spin echo, fast spin echo and inversion recovery sequences. Iran J. Radiol..

[36] Doble DMJ (2003). Toward optimized high-relaxivity MRI agents: the effect of ligand basicity on the thermodynamic stability of hexadentate hydroxypyridonate/catecholate gadolinium(III) complexes. Inorg. Chem..

[37] Woods M (2004). Solution dynamics and stability of lanthanide(III) (S)-2-(p-nitrobenzyl)DOTA complexes. Inorg. Chem..

[38] Pasha A, Tircso G, Benyo ET, Brucher E, Sherry AD (2007). Synthesis and Characterization of DOTA‐(amide)4Derivatives: Equilibrium and Kinetic Behavior of Their Lanthanide(III) Complexes. Eur. J. Inorg. Chem..

[39] Xu W (2023). Rational Design of Gd-DOTA-Type Contrast Agents for Hepatobiliary Magnetic Resonance Imaging. J. Med. Chem..

[40] Beeby, A. et al. Non-radiative deactivation of the excited states of europium, terbium and ytterbium complexes by proximate energy-matched OH, NH and CH oscillators: an improved luminescence method for establishing solution hydration states, *J. Chem. Soc. Perkin Trans. 2*, **3**, 493–503 (1999).

[41] Supkowski RM, Horrocks WD (2002). On the determination of the number of water molecules, q, coordinated to europium(III) ions in solution from luminescence decay lifetimes. Inorg. Chim. Acta..

[42] Laurent S, Elst LV, Muller RN (2006). Comparative study of the physicochemical properties of six clinical low molecular weight gadolinium contrast agents. Contrast Media Mol. Imaging.

[43] Gale EM, Zhu J, Caravan P (2013). Direct measurement of the Mn(II) hydration state in metal complexes and metalloproteins through 17O NMR line widths. J. Am. Chem. Soc..

