# Supplementary material for: Design and synthesis of chiral DOTA-based MRI contrast agents with remarkable relaxivities
Source: Commun Chem. 2023 Nov 16;6:251. doi: 10.1038/s42004-023-01050-w (PMC10654417; doi:10.1038/s42004-023-01050-w)
Supplement: Supplementary file 1 — Supporting Information [file 42004_2023_1050_MOESM1_ESM.pdf]

Supporting Information

Design and Synthesis of Chiral DOTA-based MRI Contrast Agents with remarkable Relaxivities

Junhui Zhang[1#], Lixiong Dai[1,2], Li He[1], Bhattarai Abhisek[3], Chun-Ming Chan[1], William Chi-Shing Tai[1], Varut Vardhanabhuti[3], and Ga-Lai Law[1,2]*

[1] State Key Laboratory of Chemical Biology and Drug Discovery, Department of Applied Biology and Chemical Technology, The Hong Kong Polytechnic University, Hung Hom, Hong Kong SAR, China.

[2] The Hong Kong Polytechnic University Shenzhen Research Institute, Shenzhen 518000, PR China.

[3] Department of Diagnostic Radiology, The University of Hong Kong, Hong Kong SAR, China.

[#] These authors contributed equally

E-mail: ga-lai.law@polyu.edu.hk.

Table of Content

1. Supplementary Methods 3

2. HPLC Traces 8

3. Assessments of kinetic inertness in extreme conditions 10

4. Relaxivity Measurement 21

5. Determination of thermodynamic stability constant 24

6. NMR Spectra 25

7. HRMS Spectra of Complexes 43

8. Lifetime Measurements 45

9. Variable temperature ^{17}O NMR Measurements 50

10. Supplementary References 52

1. Supplementary Methods

Synthetic Procedures

Synthesis of Ln-LS

Synthesis of compounds **1** and **2:**

Compounds **1** and **2** were synthesized according to the literature method.[12]

Synthesis of compound **4**:

Into a solution of compound **2** (400 mg, 0.47 mmol) in acetonitrile (5 mL) was added K_2CO_3 (643 mg, 4.67 mmol) and ethyl 2-bromoacetate (390 mg, 0.24 mmol), after stirring at 50°C for 16 h (the reaction was monitored by mass spectrometry), the temperature was cooled down to room temperature, filtered and the filtrate was concentrated in vacuum, then added dichloromethane (10 mL), trifluoroacetic acid (3 mL), stirred at room temperature for 2 h (the reaction was monitored by mass spectrometry), concentrated in vacuum to dryness, it was used for the next step reaction directly without further purification.

Synthesis of 2-(4-methoxy-3-sulfophenyl)acetic acid:

A solution of 2-(4-methoxyphenyl)acetic acid (5 g, 30 mmol) in dichloromethane (50 mL) was cooled to 0 – 10°C first, then added chlorosulfonic acid (4 mL, 60 mmol) slowly, the reaction mixture was stirred at room temperature for 16 h then poured into ice/water mixture (100 g) slowly (cautious: rigorous decomposition of $ClSO_3H$). After stirring for 30 min, the solvents were poured out and the product attached on the wall of flask was washed with water, then added methanol (20 mL) and water (20 mL), refluxed for 2 h, then cooled and the solvents were removed in vacuum, dried in oven to get the product as a white solid (5.6 g, 76%). ^{1}H NMR (400 MHz, D_2O) δ 7.67 (d, *J* = 2.4 Hz, 1H), 7.46 (dd, *J* = 8.6, 2.4 Hz, 1H), 7.15 (d, *J* = 8.5 Hz, 2H), 3.91 (s, 3H), 3.73 (s, 2H). ^{13}C NMR (100 MHz, D_2O) δ 176.23, 155.17, 134.15, 129.50, 128.75, 125.53, 112.58, 55.58, 39.03. *m/z* (ESI-MS$^+$) 269.12 ([M+Na]$^+$ calculated: 269.01).

Synthesis of compound **5**:

Into a solution of **4** (in the form of TFA salt) (800 mg) in DMSO (5 mL) was added NMM (1.4 g, 13.8 mmol) and 2-(4-methoxy-3-sulfophenyl)acetic acid (689 mg, 2.8 mmol), then added HATU (1.2 g, 3.2 mmol). The reaction mixture was stirred at room temperature for 12 h, the mixture was purified by reversed-semi-preparative HPLC (mobile phase A: 10 mM of ammonium formate; mobile phase B: 10 mM of ammonium formate/acetonitrile (1:9)). This resulted in the product as a light-yellow oil (660 mg, 82% for 5 steps), it was solidified after staying for several days. ^{1}H NMR (400 MHz, D_2O) δ 7.58 – 7.41 (m, 4H), 7.15 – 7.01 (m, 4H), 6.81 – 6.78 (m, 4H), 4.35 – 2.43 (m, 56H), 1.56 – 1.05 (m, 36H). ^{13}C NMR (100 MHz, D_2O) δ 173.17, 166.68, 155.06, 133.40, 130.45, 128.52, 126.97, 112.58, 62.35, 61.44, 55.74, 49.72, 48.76, 41.12, 38.73, 30.25, 28.13, 22.92, 13.56. *m/z* (ESI-MS$^+$) 450.22 ($[M\text{-}4H+4Na]^{4+}$ calculated: 450.50).

Synthesis of compound **6**.

Compound **5** (500 mg, 0.3 mmol) was dissolved in methanol (3 mL) and water (3 mL), then added $LiOH{\cdot}H_2O$ (243 mg, 5.8 mmol) in water (2 mL), after stirring at 50°C for 1 h, and then stirred at room temperature for 12 h. The pH value was adjusted to 7.0 by 1 N HCl. Concentrated in vacuum, it was used for complexation directly. ^{1}H NMR (400 MHz, D_2O) δ 7.61 – 7.59 (m, 4H), 7.40 – 7.10 (m, 4H), 7.09 – 6.82 (m, 4H), 4.20 – 2.69 (m, 36H), 1.486 – 1.38 (m, 24H). ^{13}C NMR (100 MHz, D_2O) δ 173.74, 170.05, 155.28, 133.68, 130.24, 128.09, 126.98, 112.71, 55.81, 48.89, 41.30, 41.25, 38.95, 38.91, 28.37, 24.57, 23.22. *m/z* (ESI-MS$^+$) 422.02 ($[M\text{-}4H+4Na]^{4+}$ calculated: 422.13).

General procedure of synthesis complexes of **Ln-LS**:

Ligand **6** (20 mg) was dissolved in water (2 mL), then added $LnCl_3{\cdot}6H_2O$ (1.05 eq.), the pH value was adjusted to 7.0 and then refluxed for one night, the temperature was cooled down to room temperature, the pH value was adjusted to 11, filtered through a 0.22 mm syringe filter to remove hydroxide from the excess of metal ions, then the pH value was adjusted back to 7.0, two isomers were isolated by semi-preparative HPLC and the fractions were lyophilized to give a white powder (yields ~85%). **Eu-LS**: *m/z* (ESI-MS$^+$) 1751.4251 ($[M+2H]^+$ calculated: 1751.4262). **Gd-LS**: *m/z* (ESI-MS$^+$) 1756.4326 ($[M+2H]^+$ calculated: 1756.4290).

Synthesis of Ln-T

Synthesis of compound **13**.

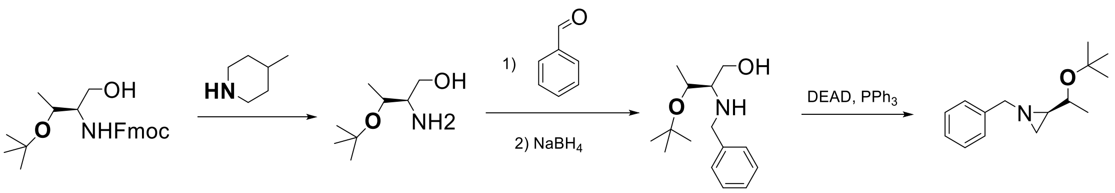

7 8 9 10

Figure S 1 Synthesis of aziridine compound **10**.

Synthesis of compound **8**:

Compound **7** (15 g, 39 mmol) in DCM (100 mL) was added 4-methyl piperidine (10 mL), reacted at room temperature for 3 h, concentrated and added methanol (50 mL), cooled to 0 – 10°C and filtered, the filtrate was concentrated and this resulted in the product **8** (6 g, crude) which was used to the next step reaction directly without further purification.

Synthesis of compound **9**:

Compound **8** (6 g, crude) was dissolved in methanol (18 mL) and DCM (78 mL), then added benzaldehyde (4.15 g, 39 mmol), after reacting at room temperature for 16 h, the concentrated and then dissolved in methanol (150 mL), added $NaBH_4$ (2.9 g, 76 mmol), after reacting at room temperature for 30 min, the mixture was concentrated in vacuum and purified by silica gel column chromatography with ethyl acetate and petroleum ether (1:5 to 2:1). This resulted in the product (6 g, yield 65%) as a colorless oil. ^1H NMR (400 MHz, $CDCl_3$) δ 7.33 – 7.31 (m, 5H), 3.97 – 3.60 (m, 4H), 3.60 – 3.35 (m, 1H), 2.70 – 2.47 (m, 1H), 1.18 (s, 12H). ^{13}C NMR (100 MHz, $CDCl_3$) δ 140.47, 128.47, 127.90, 127.00, 74.24, 67.96, 63.07, 60.14, 51.81, 28.65, 19.47. m/z (ESI-MS$^+$) 252.28 ($[M+H]^+$ calculated: 252.37), 274.29 ($[M+Na]^+$ calculated: 274.36).

Synthesis of compound **10**:

Into a soliton of **9** (5.5 g, 22 mmol) in THF (100 mL) was added with PPh_3 (8.1 g, 31 mmol), then cooled the reaction mixture to 0 – 10°C, then added DIAD (5.7 g, 31 mmol)

dropwise, the resulted mixture was reacted for 16 h at room temperature, then concentrated and added 200 mL of hexane to form precipitate, the solid was removed by filtration. The filtrate was concentrated and the residue was purified by silica gel column chromatography with ethyl acetate and petroleum ether (1:20 to 1:5). This resulted in the product (4 g, yield 78%) as a colorless oil. ^{1}H NMR (400 MHz, $CDCl_3$) δ 7.52 – 7.30 (m, 5H), 3.38 (d, *J* = 2.0 Hz, 2H), 3.30 (t, *J* = 6.4 Hz, 1H), 1.70 – 1.59 (m, 2H), 1.40 (d, *J* = 6.3 Hz, 1H), 1.11 (s, 9H), 1.08 (d, *J* = 6.4 Hz, 3H). ^{13}C NMR (100 MHz, $CDCl_3$) δ 139.30, 128.56, 128.25, 127.02, 73.35, 69.44, 64.95, 45.38, 31.37, 28.53, 20.43. m/z (ESI-MS$^+$) 234.37 ($[M+H]^+$ calculated: 234.36).

Synthesis of compound **11**:

To a solution of **10** (2.7 g, 11.6 mmol) in dried benzene (27 mL) was added $BF_3 \cdot Et_2O$ (0.093 mL), the mixture was stirred under reflux for 24 h. Then the temperature was cooled down to room temperature, 2 mL of saturated sodium bicarbonate was added, then concentrated and the residue was purified by recrystallization in benzene and ethyl acetate, inorganic salts were washed by water. The solid was dried in the oven and this resulted in the product as a white solid (400 mg, 14.8%). ^{1}H NMR (400 MHz, $CDCl_3$) δ 7.60 – 6.81 (m, 20H), 4.62 (d, *J* = 12.6 Hz, 4H), 3.89 (dd, *J* = 6.2, 1.8 Hz, 4H), 3.01 (d, *J* = 12.6 Hz, 4H), 2.85 – 2.47 (m, 12H), 1.18 (d, *J* = 6.2 Hz, 12H), 0.81 (s, 36H). ^{13}C NMR (100 MHz, $CDCl_3$) δ 141.80, 130.44, 127.72, 126.31, 72.57, 68.01, 58.17, 56.96, 45.34, 28.81, 22.07. m/z (ESI-MS$^+$) 933.7180 ($[M + H]^+$ calculated: 933.7197).

Synthesis of compound **12**:

To a solution of **11** (400 mg, 0.43 mmol) in trifluoroethanol (15 mL) was added $Pd(OH)_2/C$ (20% loading, wet with 50% of water) (200 mg) and ammonium formate (500 mg), after reacting at 50°C for 16 h, a filtration was performed and the filtrate was concentrated in vacuum to get the product a white solid (232 mg, yield 95%). ^{1}H NMR (400 MHz, $CDCl_3$) δ 4.00 – 3.87 (m, 4H), 2.94 – 2.91 (m, 4H), 2.66 – 2.63 (m, 4H), 2.42 – 2.37 (m, 4H), 1.18 (s, 36H), 1.03 (d, *J* = 6.4 Hz, 12H). ^{13}C NMR (100 MHz, $CDCl_3$) δ 73.63, 65.51, 56.12, 43.65, 28.44, 15.89. m/z (ESI-MS$^+$) 596.75 ($[M+Na]^+$ calculated: 595.92).

Synthesis of compound **14**:

Into a solution of compound **12** (600 mg, 1.0 mmol) in acetonitrile (5 mL) was added K_2CO_3 (1.4 g, 10 mmol) and tert-butyl 2-bromoacetate (1.2 g, 6.0 mmol), after stirring at 50°C for 16 h (the reaction was monitored by mass spectrometry), the temperature was cooled down to room temperature, filtered and the filtrate was concentrated in vacuum, then added trifluoroacetic acid (5 mL), stirred at room temperature for 12 h, concentrated in vacuum to dryness, added methanol (2 mL) and diethyl ether (20 mL), filtered and washed with diethyl ether, this resulted in the product in the form of TFA salt as a light yellow solid (800 mg, 82%). ^{1}H NMR (400 MHz, D_2O) δ 4.37 – 2.19 (m, 24H), 1.21 – 1.20 (m, 12H). ^{13}C NMR (100 MHz, D_2O) δ 180.32, 66.00, 58.30, 44.66, 29.67, 20.10. m/z (ESI-MS$^+$) 581.55 ([M+H]$^+$ calculated: 581.63).

General procedure of synthesis complexes of **Ln-T**:

Ligand **14** (20 mg) was dissolved in water (2 mL), then added $LnCl_3 \cdot 6H_2O$ (1.05 eq.), the pH value was adjusted to 7.0 and then refluxed for one night, the temperature was cooled down to room temperature, the pH value was adjusted to 11, filtered through a 0.22 mm syringe filter to remove the Ln hydroxide from the excess of metal ions, then the pH value was adjusted back to 7.0, the resulting solution was further purified by reverse phase semi-preparative HPLC (A: 98% 10 mM ammonium formate, B: 2% ACN). Fractions were lyophilized to give a white powder. **Gd-T**: *m/z* (ESI-MS$^-$) 734.1864 ([M]$^-$ calculated: 734.1884). **Eu-T**: m/z (ESI-MS$^-$) 729.1847 ([M]$^-$ calculated: 729.1855).

2. HPLC Traces

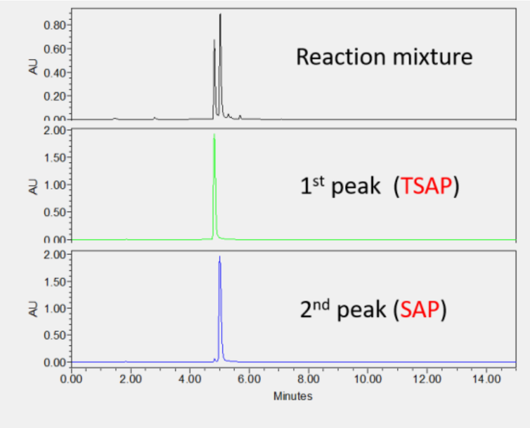

Figure S 2 HPLC traces of Eu-LS. Mixture before isolation (top); 1st peak after isolation (middle); 2nd peak after isolation (bottom) (Method A).

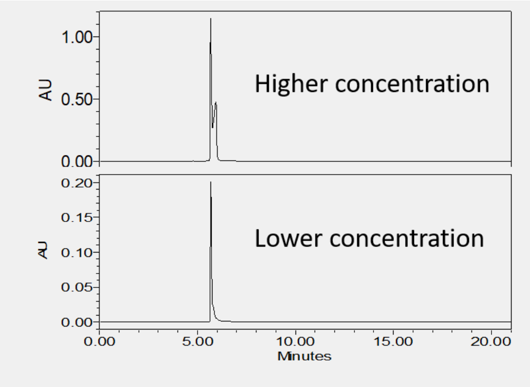

Figure S 3 HPLC traces of Gd-LS (TSAP isomer). At higher concentration (upper) and lower concentration (lower) (Method A).

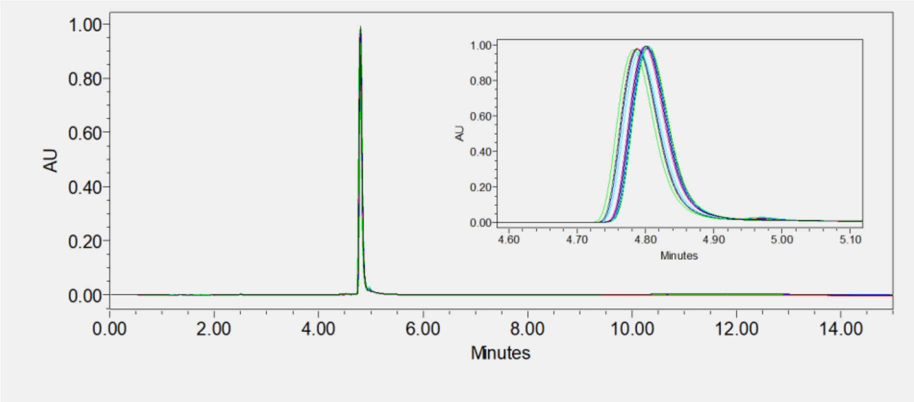

Figure S 4 HPLC traces of Gd-LS-1st in 0.1 N HCl at room temperature over time for 7 days, the zoom-in graph was inserted (Method A).

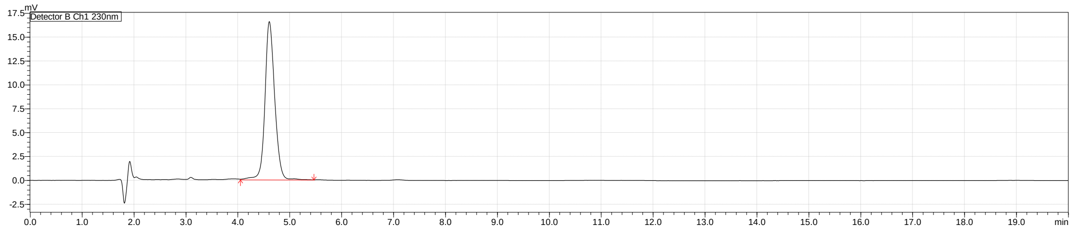

Figure S5 HPLC trace of SAP isomer of **Eu-T**, retention time at 4.61 min (Method B).

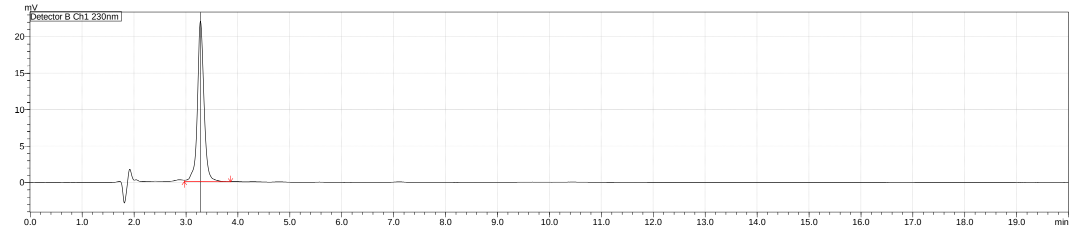

Figure S6 HPLC trace of TSAP isomer of **Eu-T**, retention time at 3.28 min (Method B).

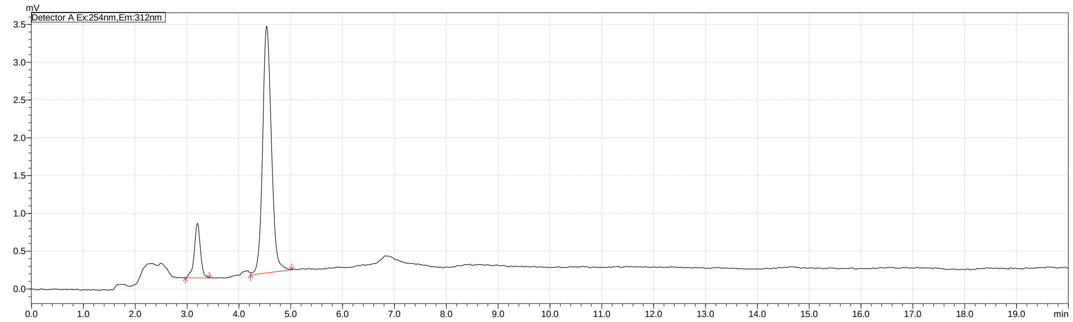

Figure S7 HPLC trace of **Gd-T** before purification. Retention time of first peak (TSAP, 13.3 %) at 3.20 min, second peak (SAP, 86.7 %) at 4.54 min (Method B).

3. Assessments of kinetic inertness in extreme conditions

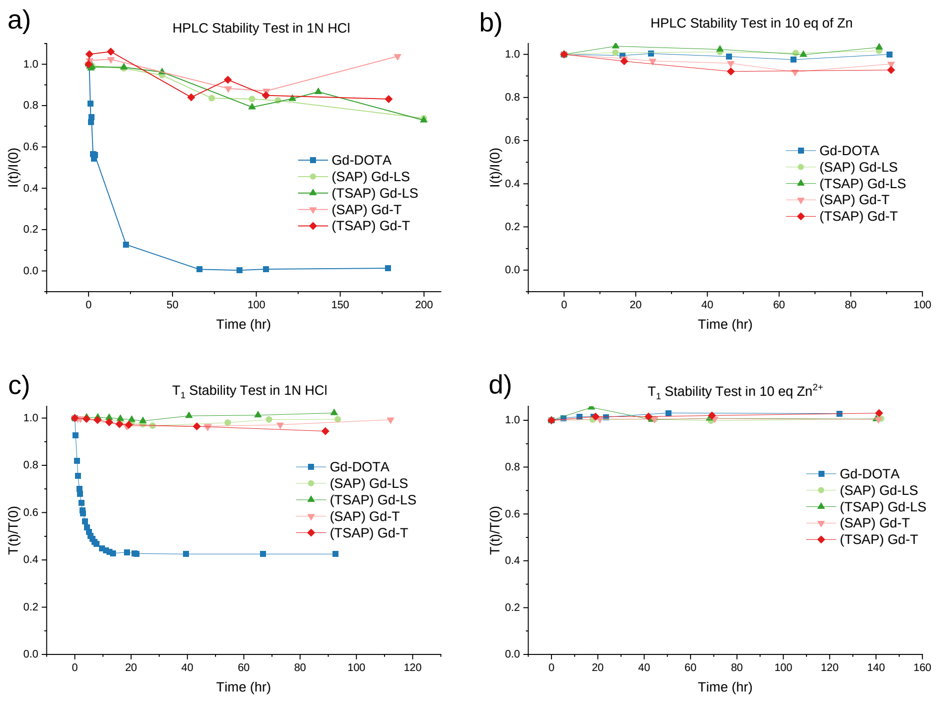

Figure S8 Assessments of kinetic inertness of **Gd-DOTA**, **(SAP)Gd-LS**, **(TSAP)Gd-LS**, **(SAP)Gd-T** and **(TSAP)Gd-T** in extreme conditions; **a)** Evolution of the integrated area of complex peak, I(t)/I(0), as a function of time under 1 N HCl at 37°C. **b)** Evolution of the integrated area of complex peak, I(t)/I(0), as a function of time under 10 eq. of Zn^{2+} at 37°C, ZnCl was used as ion source. **c)** Evolution of T_1 relaxation time, $T_1(t)/T_1(0)$, as a function of time under 1 N HCl at 37°C, 1.4T. **d)** Evolution of T_1 relaxation time, $T_1(t)/T_1(0)$, as a function of time under under 10 eq. of Zn^{2+} at 37°C, 1.4 T, ZnCl was used as ion source.

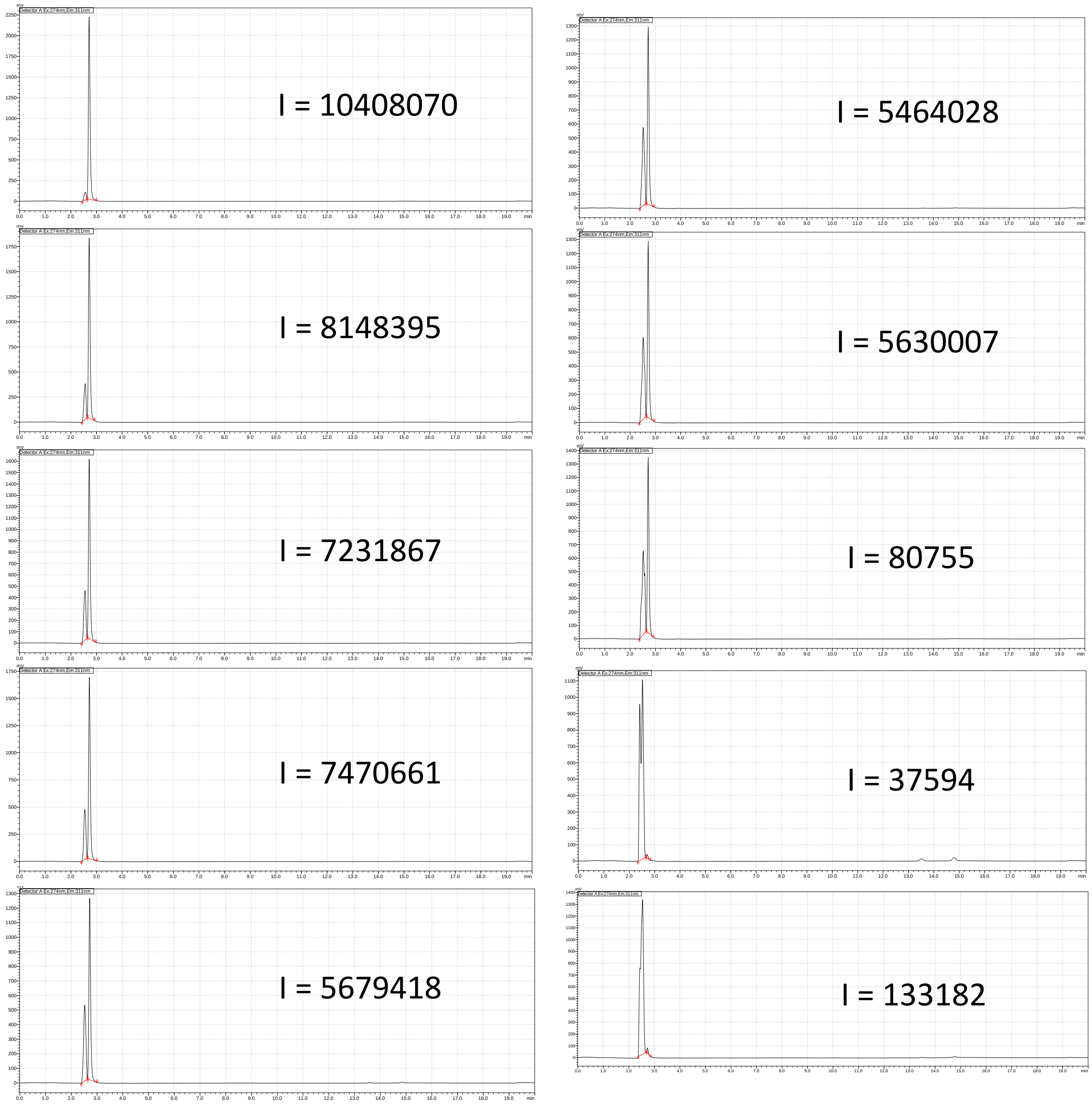

Figure S 9 HPLC traces of **Gd-DOTA** (Dotatem®) in 1 N HCl at various time points.

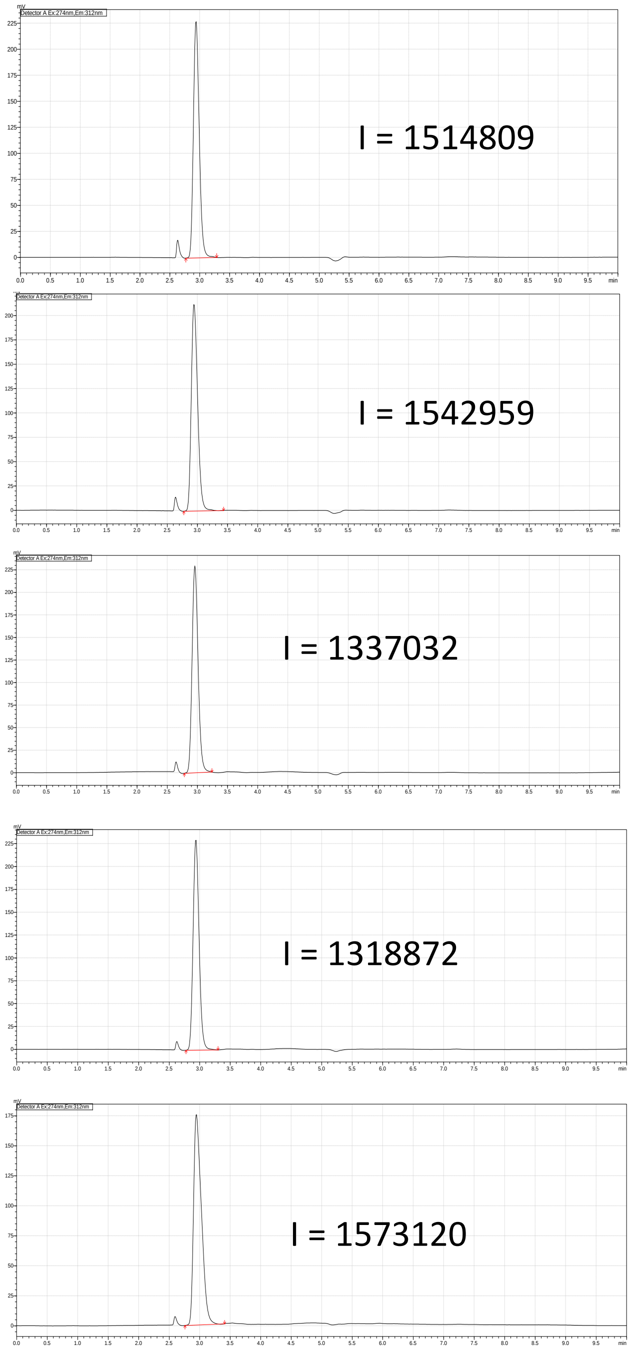

Figure S 10 HPLC traces of **(SAP)Gd-T** in 1 N HCl at various time points.

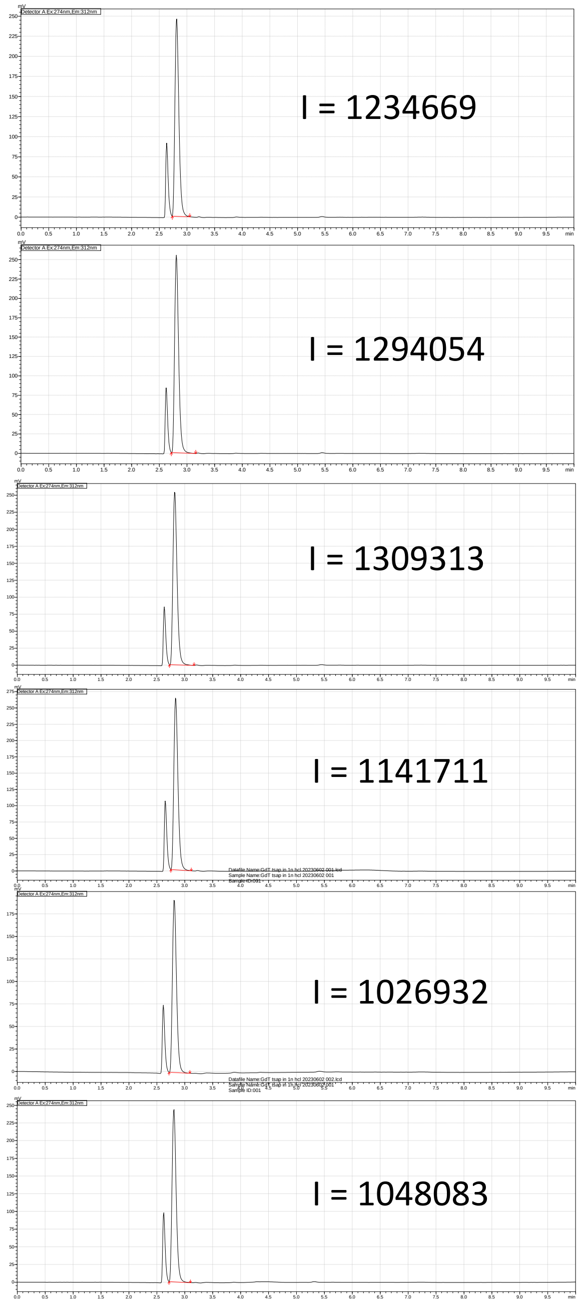

Figure S 11 HPLC traces of **(TSAP)Gd-T** in 1 N HCl at various time points.

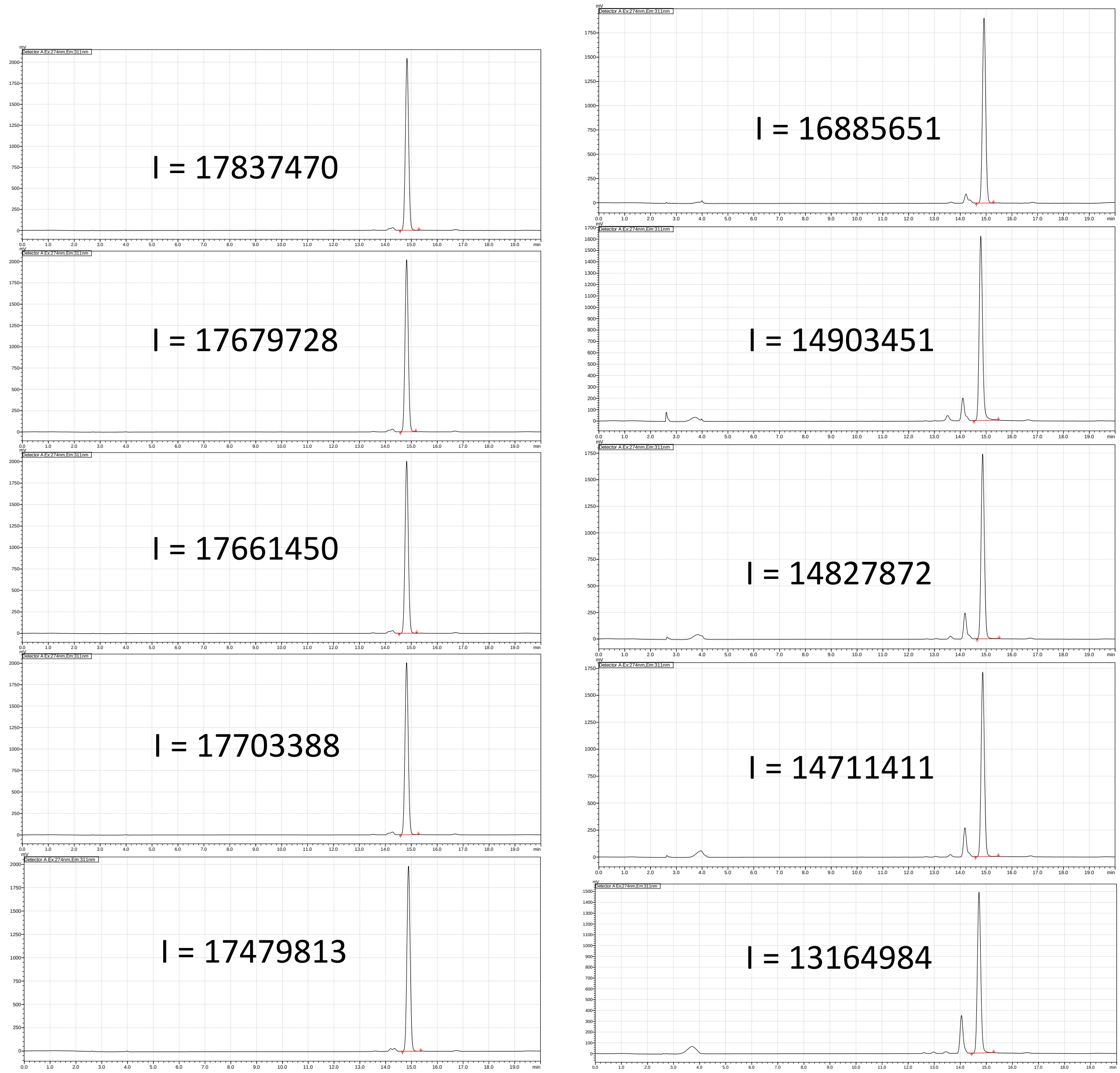

Figure S 12 HPLC traces of **(SAP)Gd-LS** in 1 N HCl at various time points.

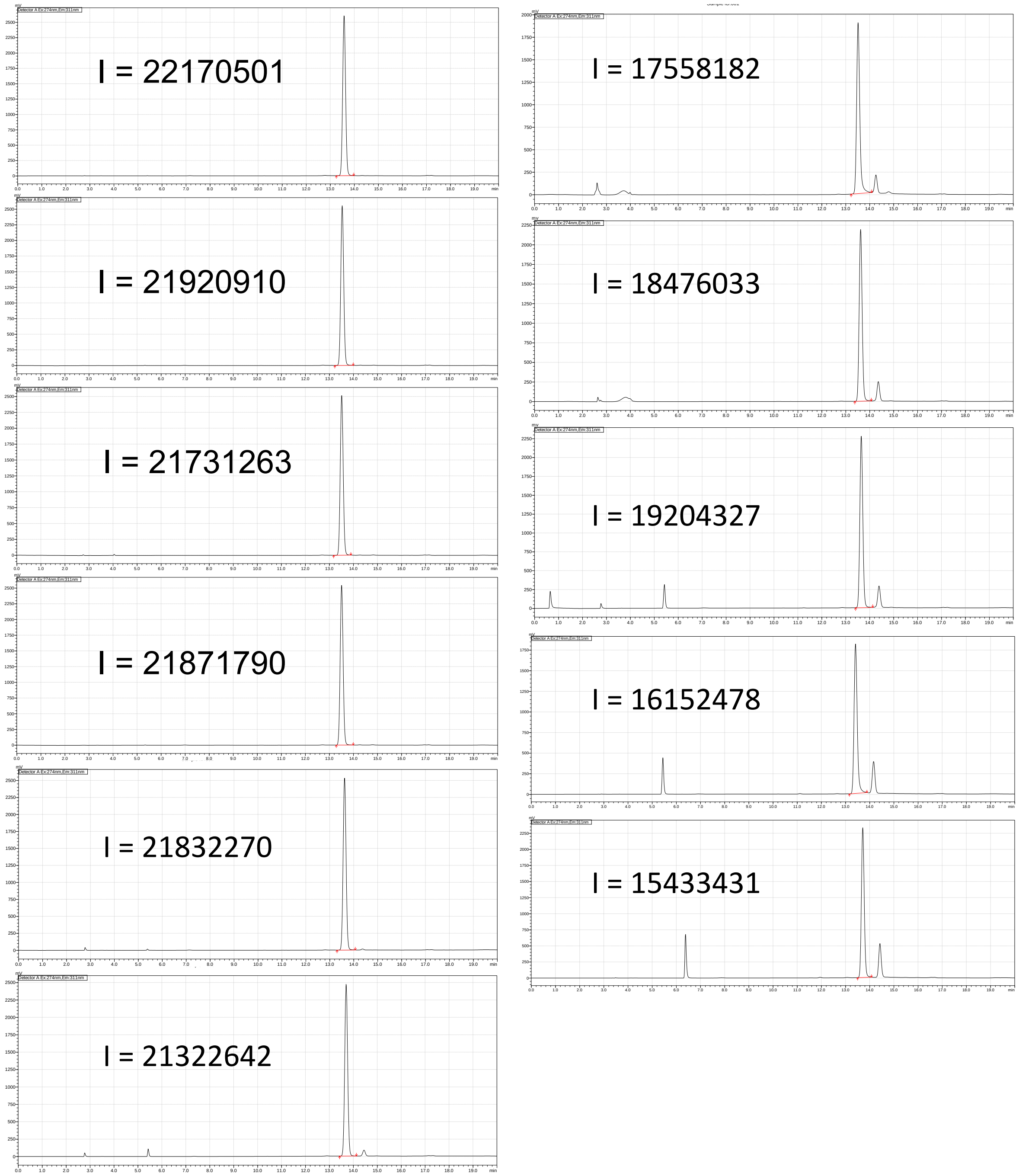

Figure S 13 HPLC traces of **(TSAP)Gd-LS** in 1 N HCl at various time points.

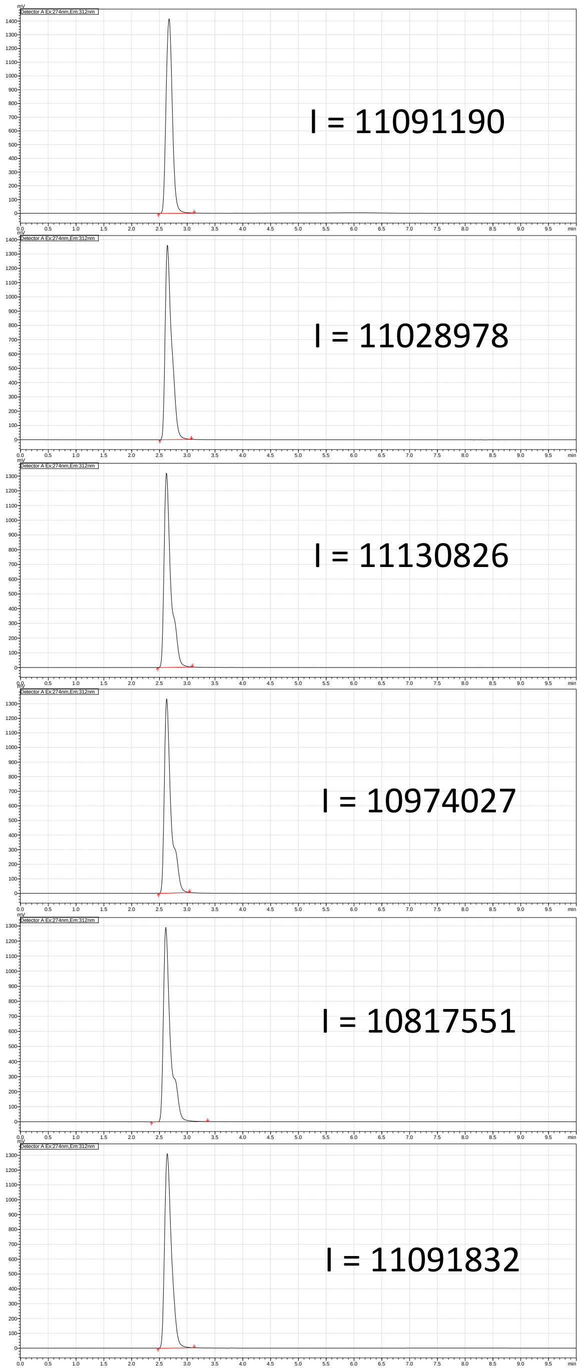

Figure S 14 HPLC traces of **Gd-DOTA** (Dotatem®) in 10 eq. of Zn at various time points.

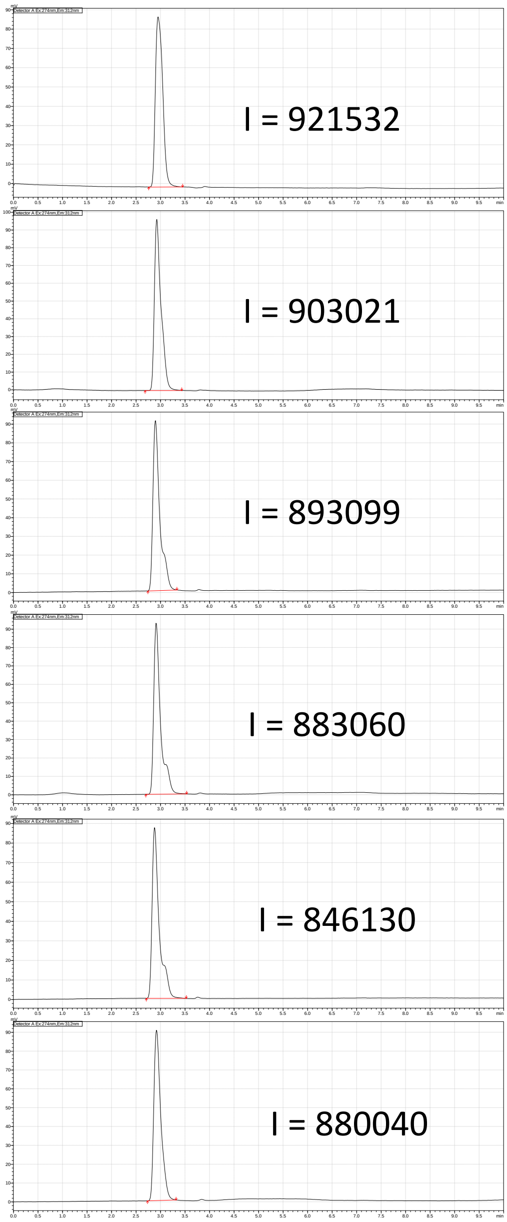

Figure S 15 HPLC traces of **(SAP)Gd-T** in 10 eq. of Zn at various time points.

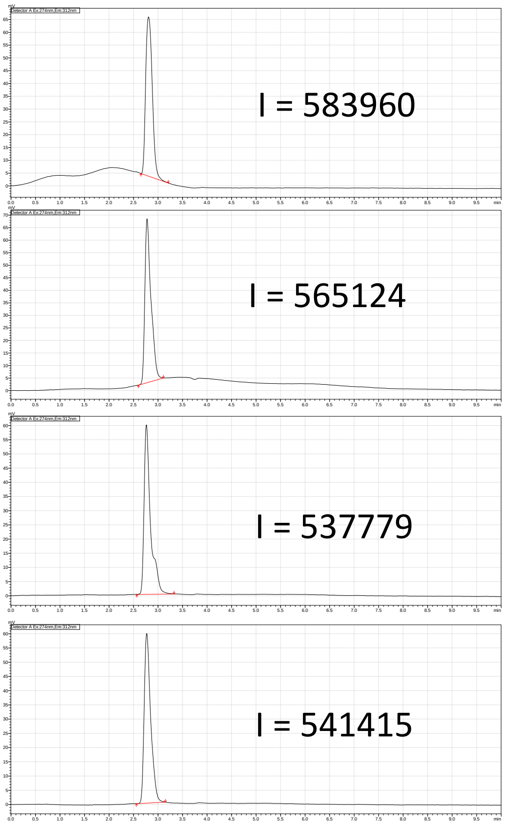

Figure S 16 HPLC traces of **(TSAP)Gd-T** in 10 eq. of Zn at various time points.

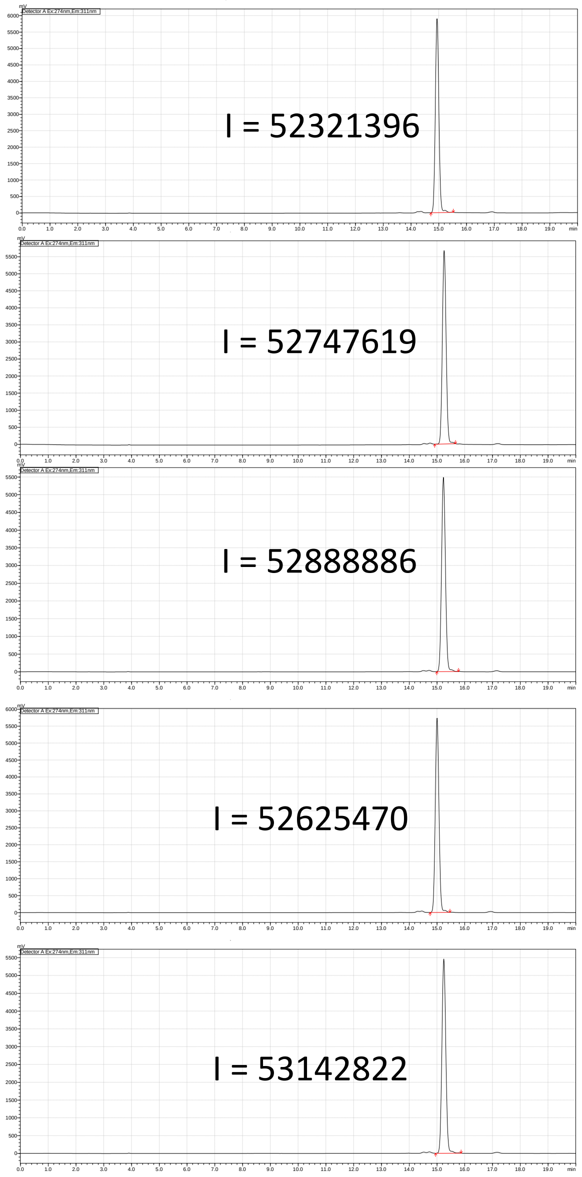

Figure S 17 HPLC traces of **(SAP)Gd-LS** in 10 eq. of Zn at various time points.

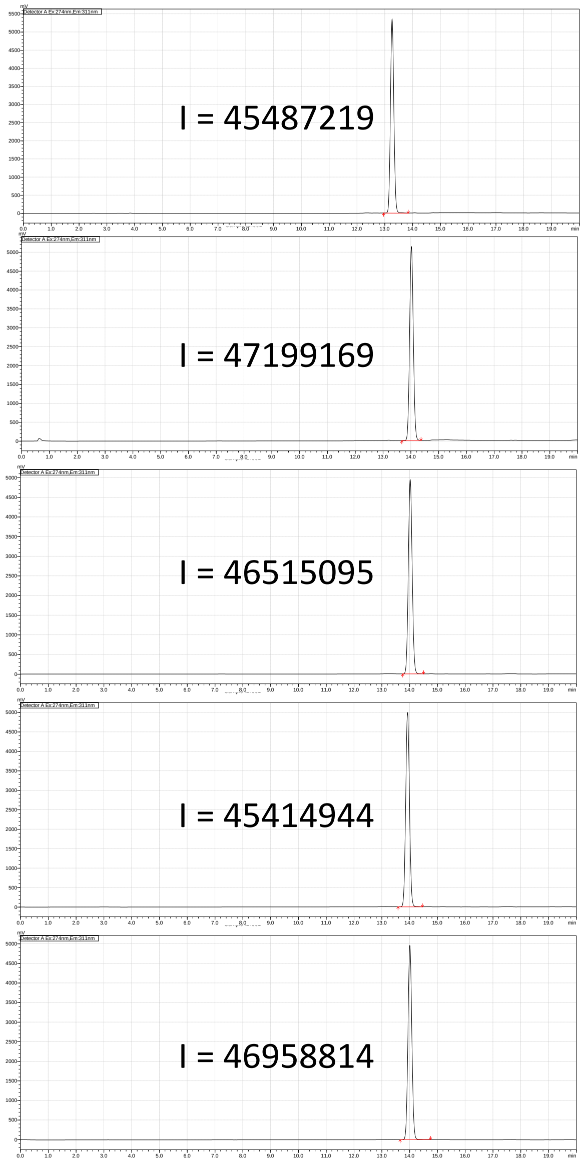

Figure S 18 HPLC traces of **(TSAP)Gd-LS** in 10 eq. of Zn at various time points.

4. Relaxivity Measurement

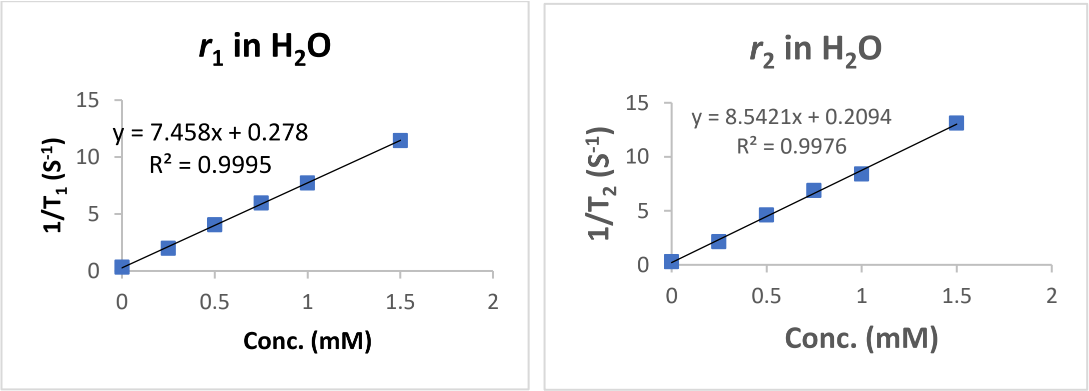

Figure S19 r_1 and r_2 of (TSAP)Gd-LS in water. Longitudinal (r_1) relaxation rate (left); Transversal (r_2) relaxation rate (right).

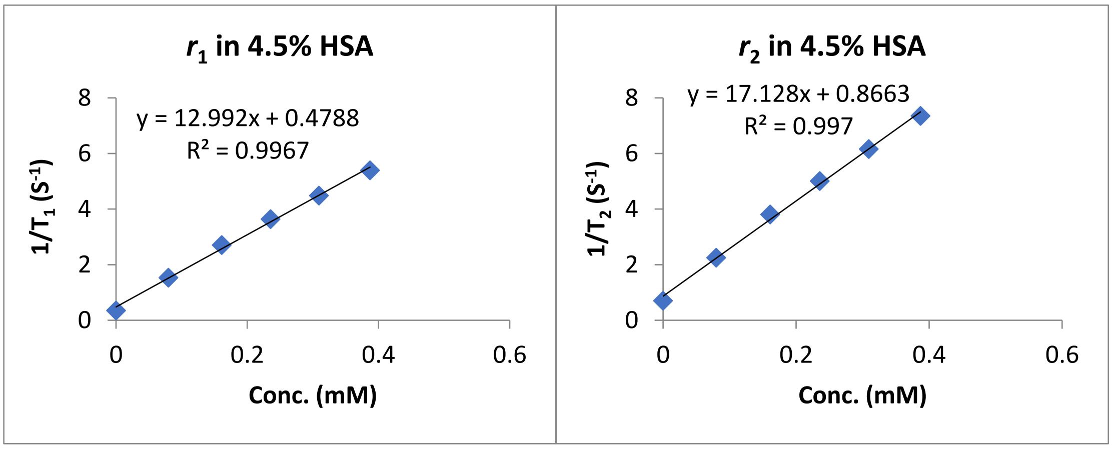

Figure S20 r_1 and r_2 of (TSAP)Gd-LS in 4.5% HSA. Longitudinal (r_1) relaxation rate (left); Transversal (r_2) relaxation rate (right).

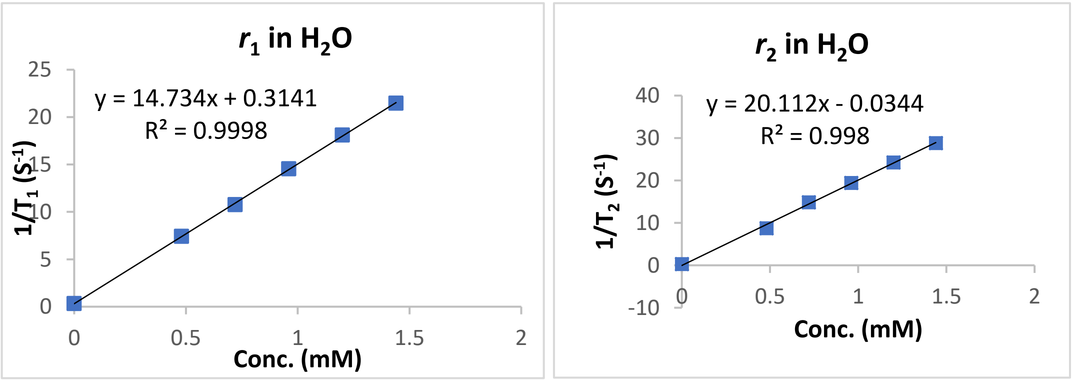

Figure S21 r_1 and r_2 of (SAP)Gd-LS in water. Longitudinal (r_1) relaxation rate (left); Transversal (r_2) relaxation rate (right).

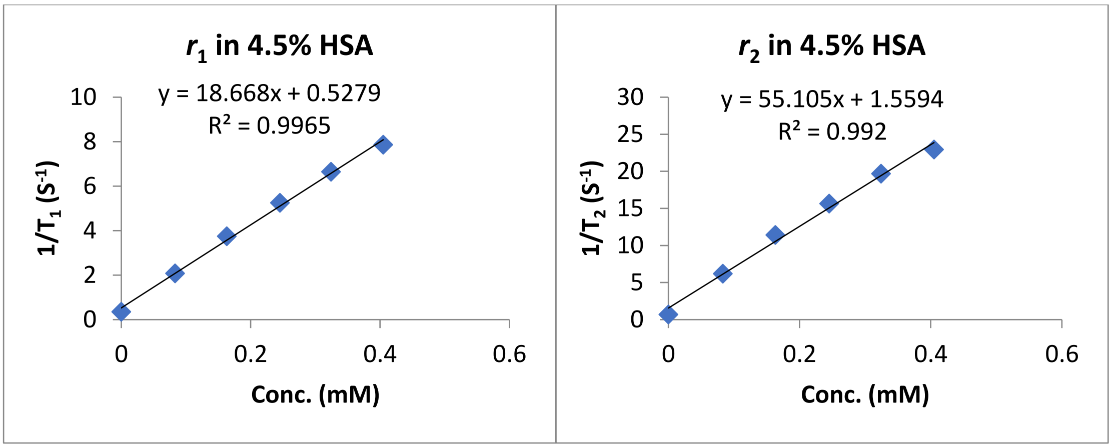

Figure S22. r_1 and r_2 of (SAP)Gd-LS in 4.5% HSA. Longitudinal (r_1) relaxation rate (left); Transversal (r_2) relaxation rate (right).

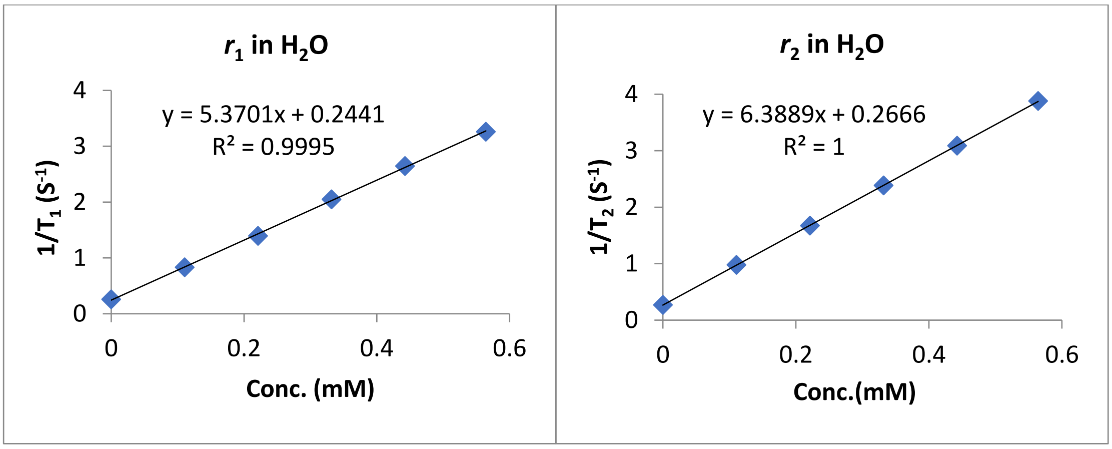

Figure S23. r_1 and r_2 of (SAP)Gd-T in water. Longitudinal (r_1) relaxation rate (left); Transversal (r_2) relaxation rate (right).

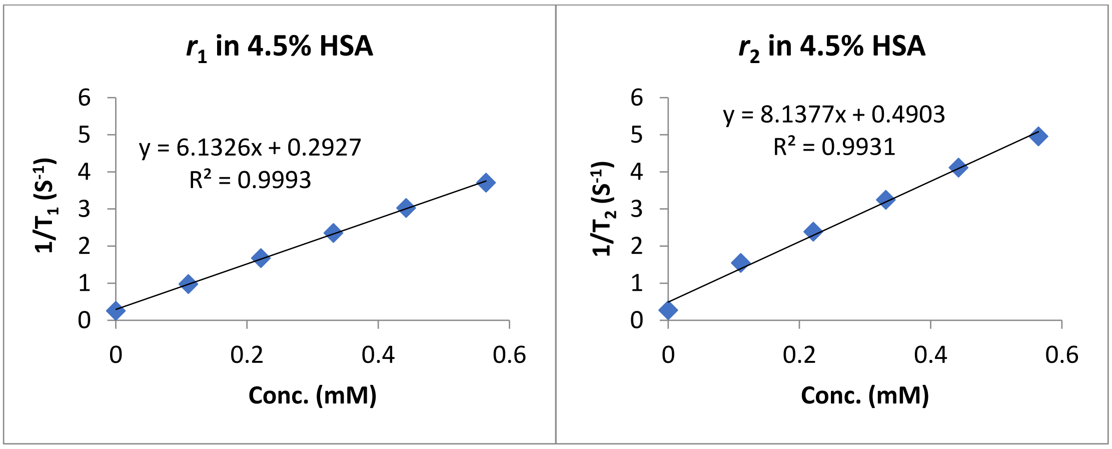

Figure S24. r_1 and r_2 of (SAP)Gd-T in 4.5% HSA. Longitudinal (r_1) relaxation rate (left); Transversal (r_2) relaxation rate (right).

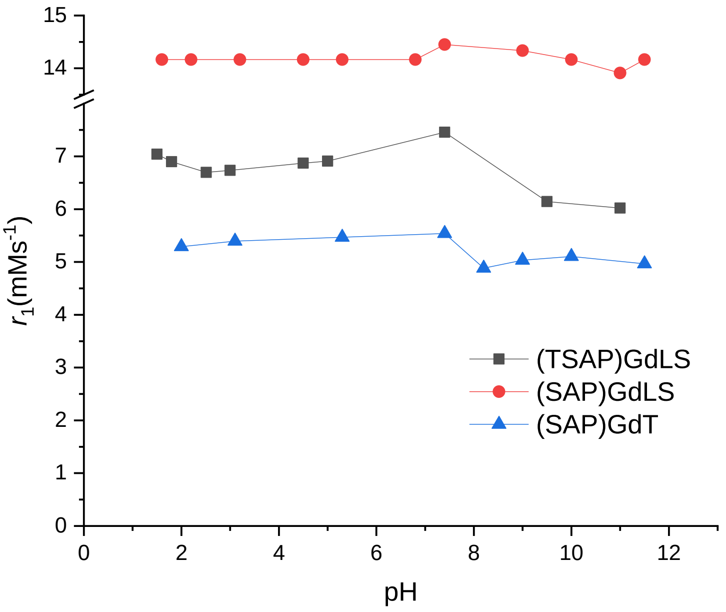

Figure S25 pH dependent r1 relaxivity measurements for (TSAP)GdLS, (SAP)GdLS and (SAP)GdT in 1X PBS buffer, pH was adjusted by 1 N NaOH and 1 N HCl.

5. Determination of thermodynamic stability constant

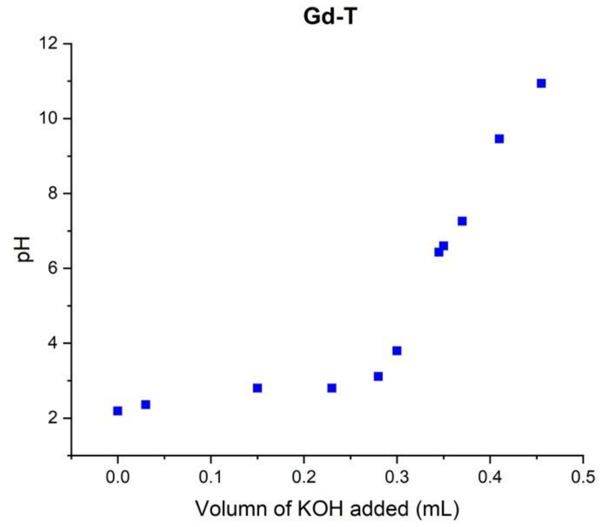

Figure S 26 Titration curve for determining Gd-T stability constant.

6. NMR Spectra

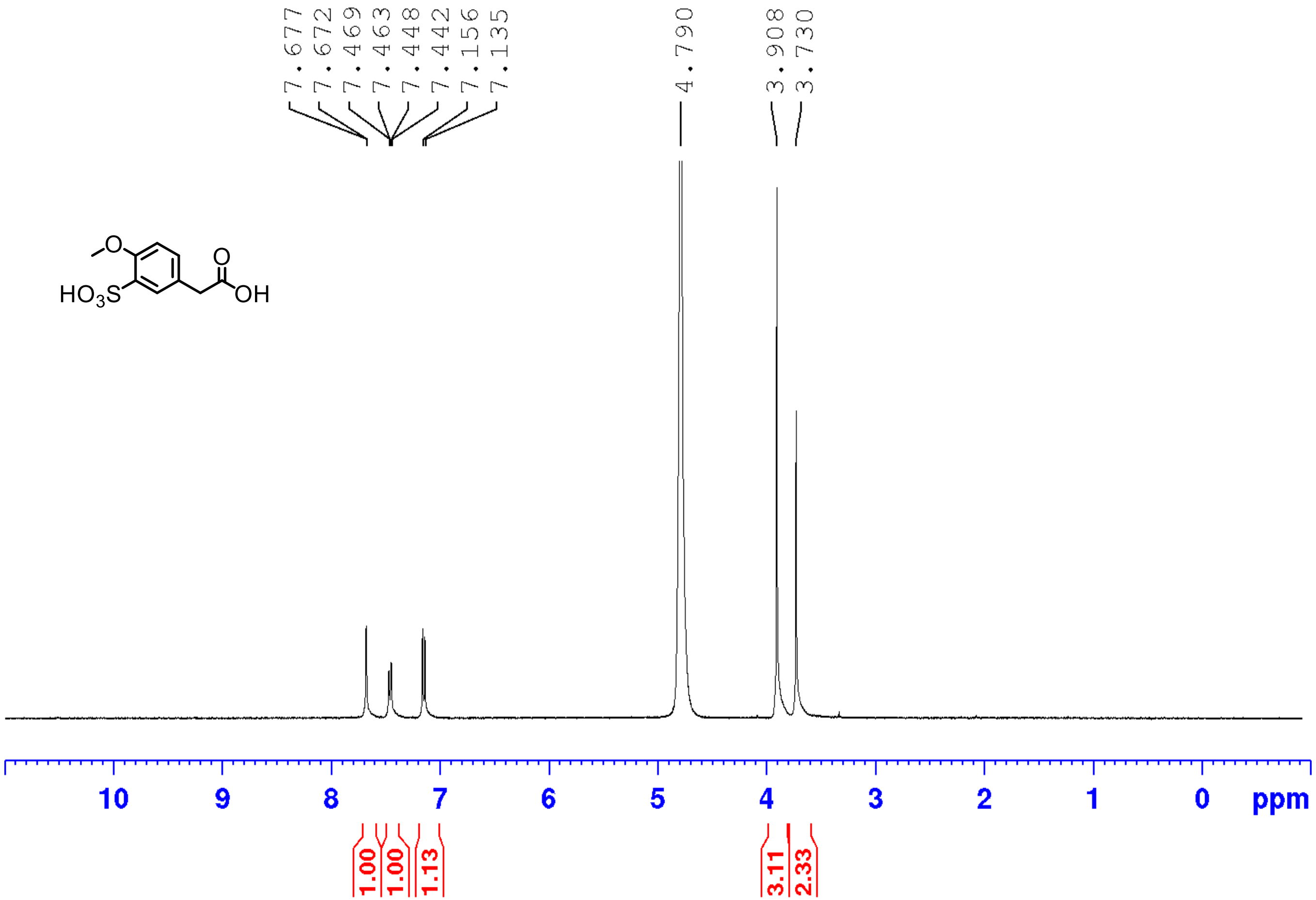

Figure S27. The ^{1}H NMR spectrum of 2-(4-methoxy-3-sulfophenyl)acetic acid in D_2O, 298 K.

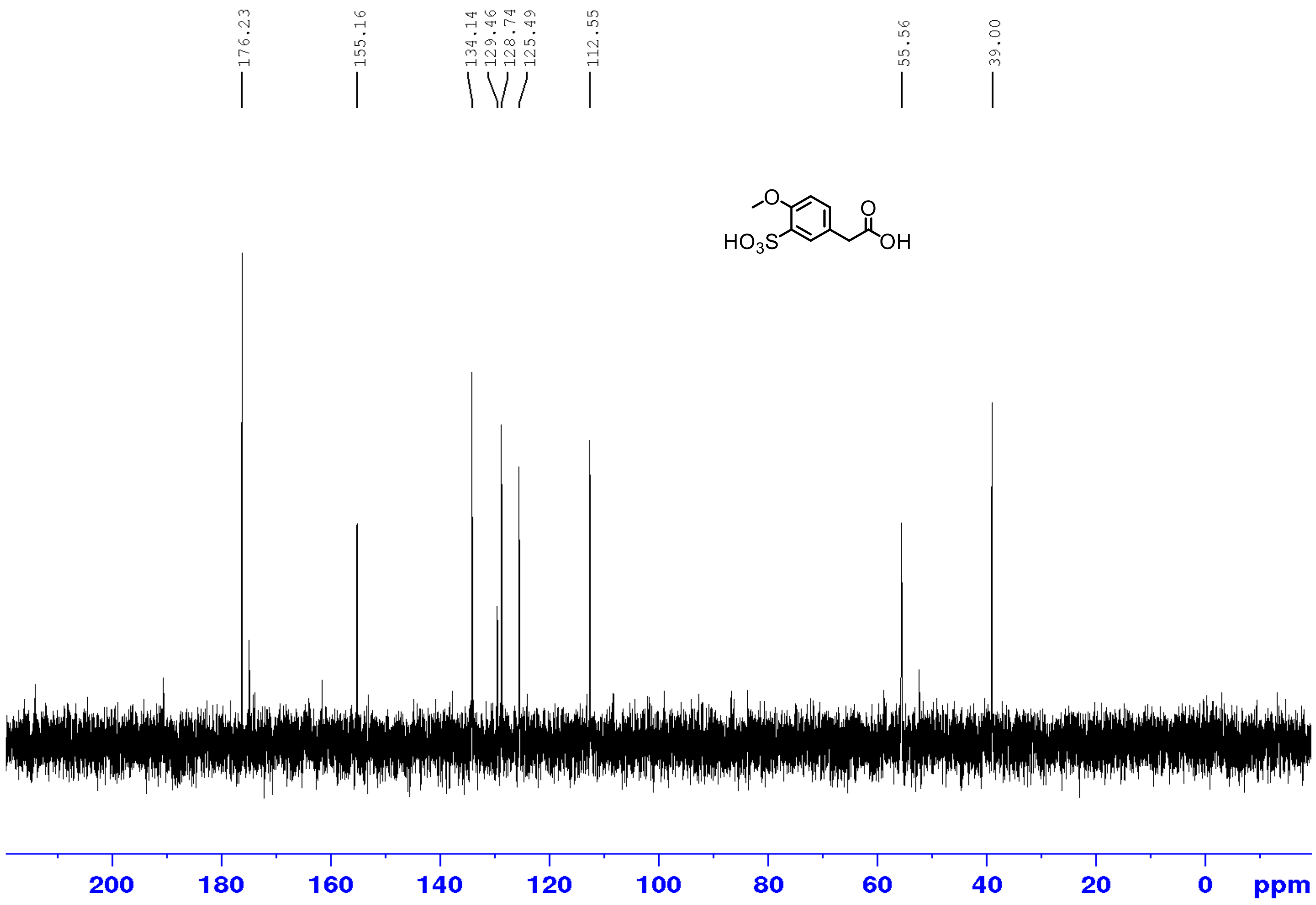

Figure S28. The ^{13}C NMR spectrum of 2-(4-methoxy-3-sulfophenyl)acetic acid in D_2O, 298 K.

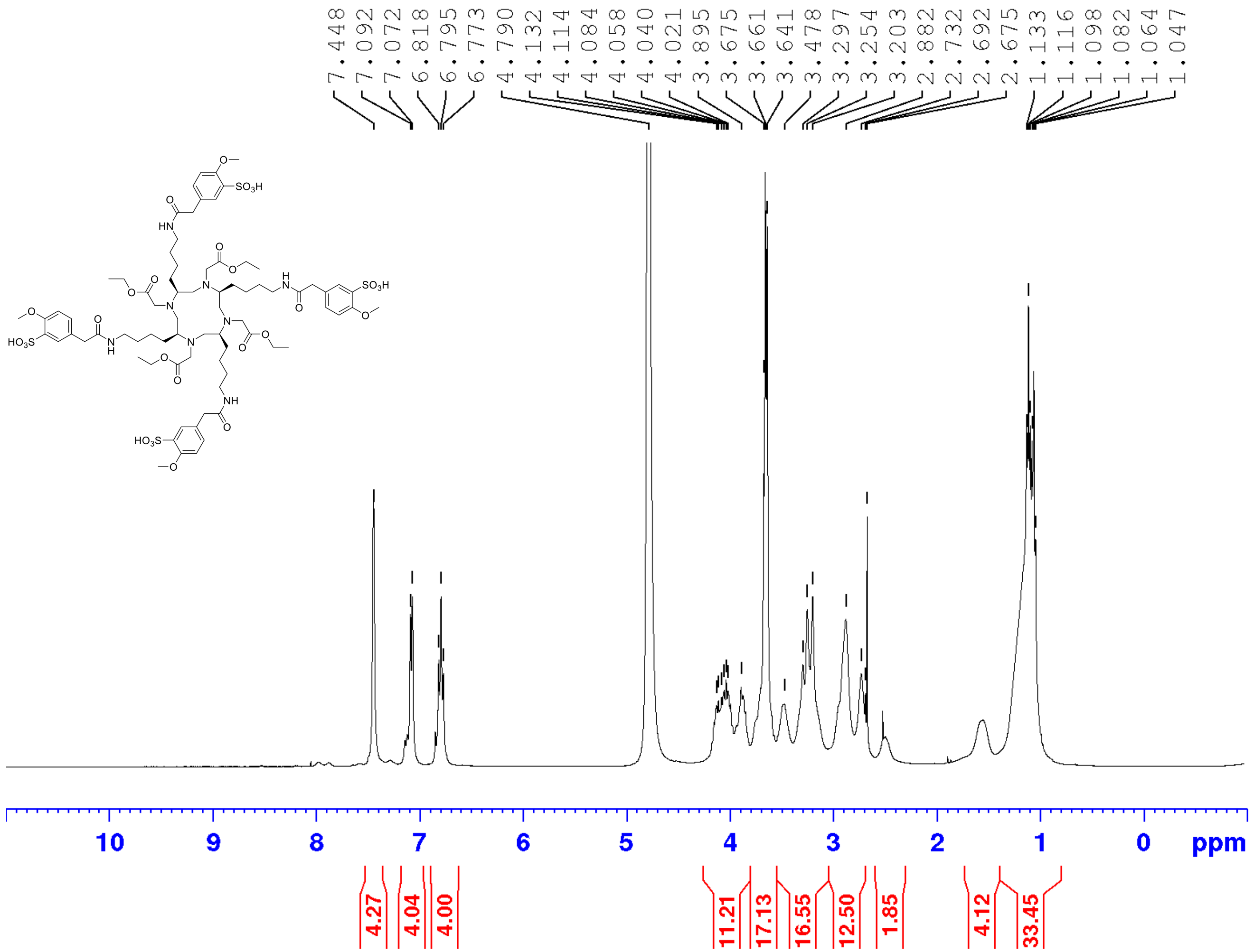

Figure S29. The ^{1}H NMR spectrum of compound **5** in D_2O, 298 K.

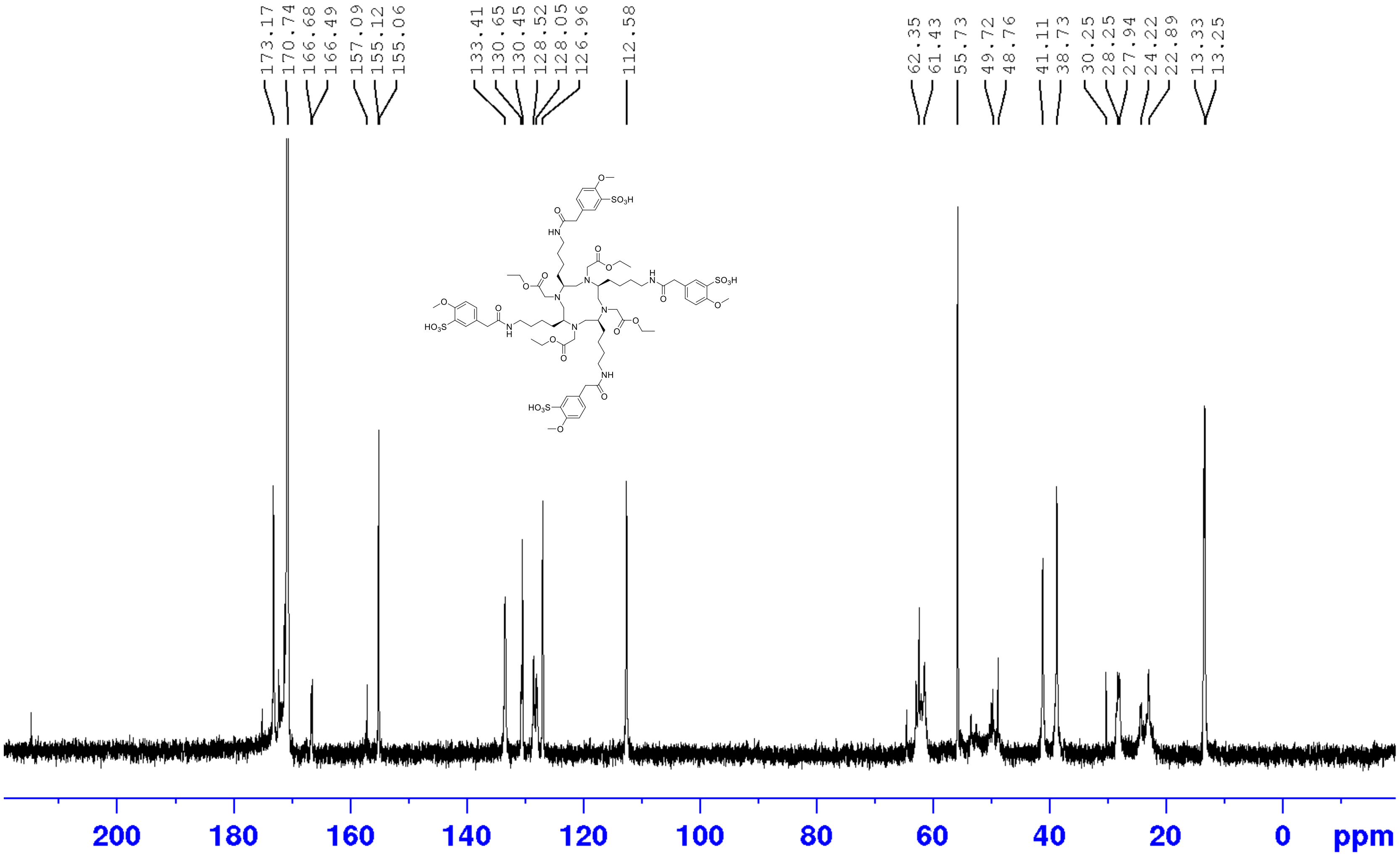

Figure S30. The ^{13}C NMR spectrum of compound **5** in D_2O, 298 K.

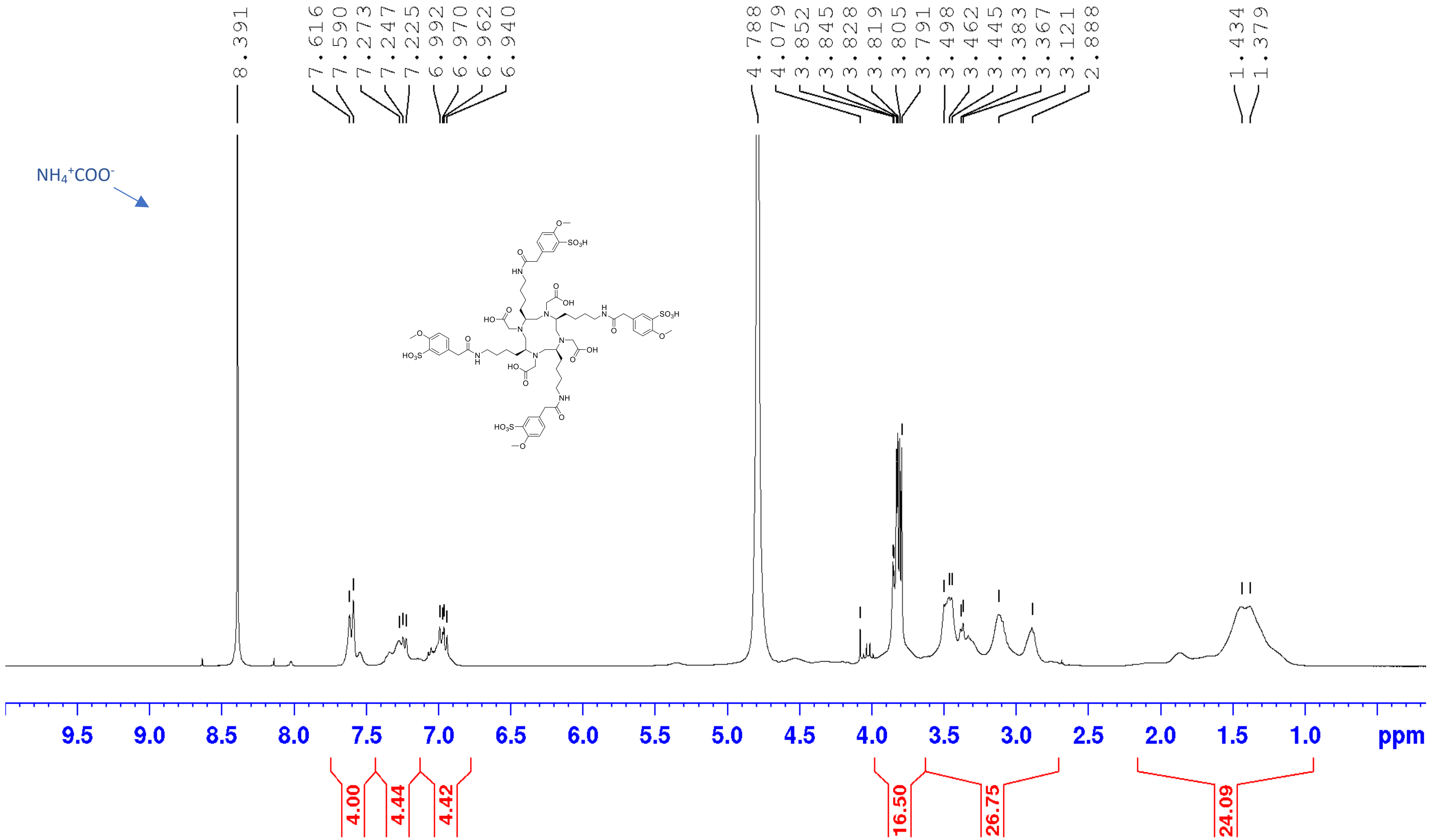

Figure S31. The ^{1}H NMR spectrum of compound **6** in D_2O, 298 K.

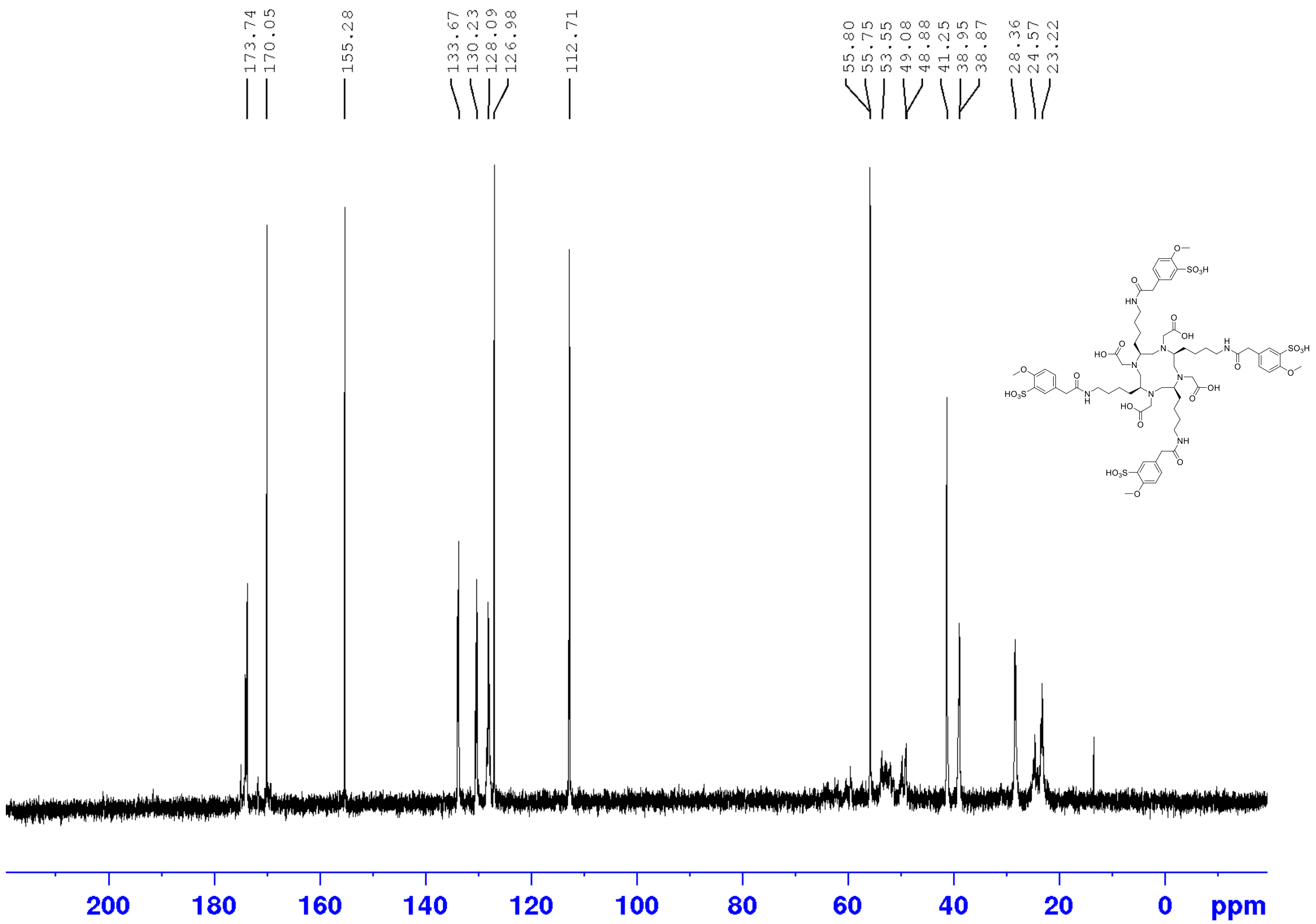

Figure S32. The ^{13}C NMR spectrum of compound **6** in D_2O, 298 K.

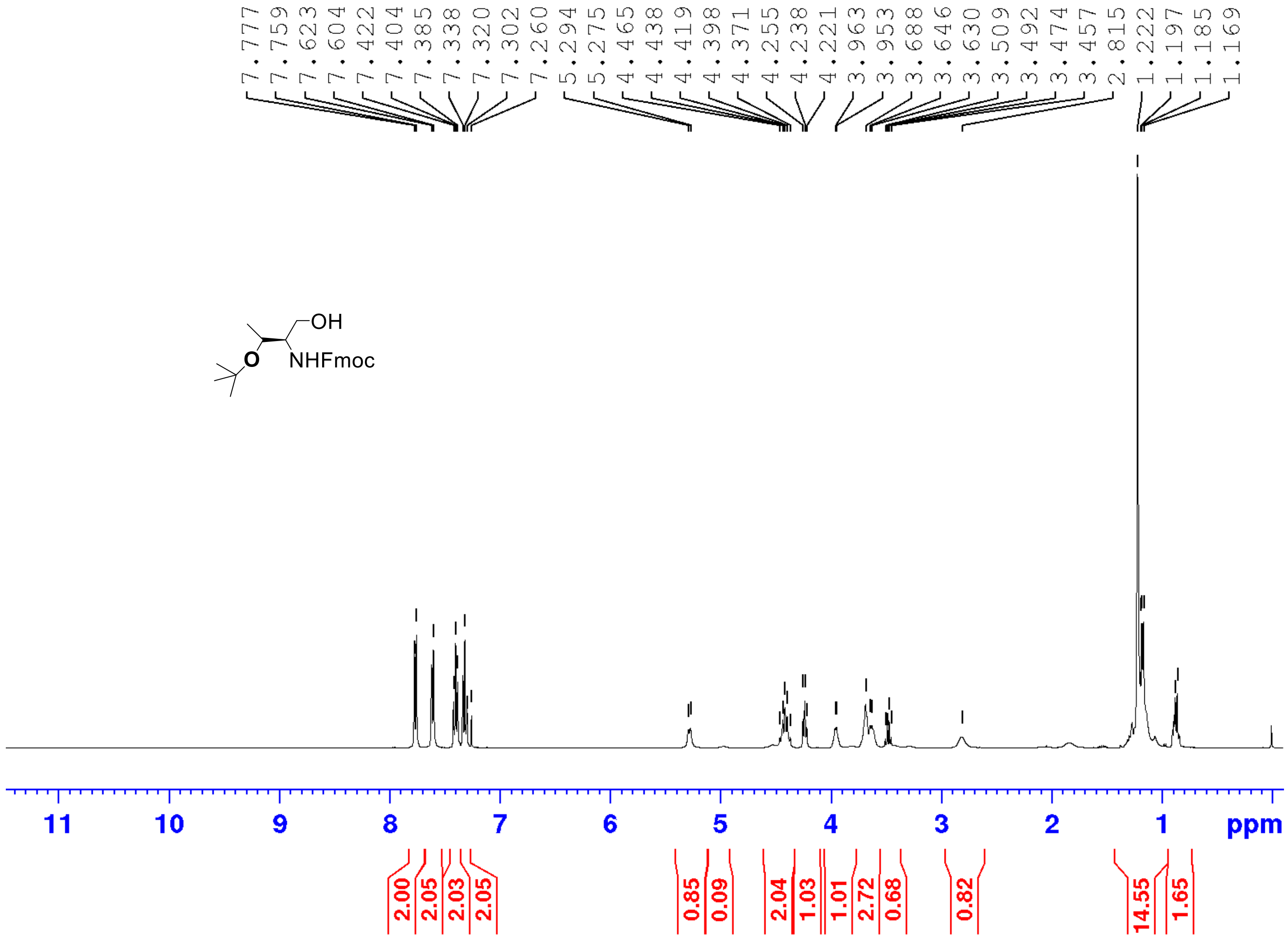

Figure S33. The ^{1}H NMR spectrum of compound **7** in $CDCl_3$, 298 K.

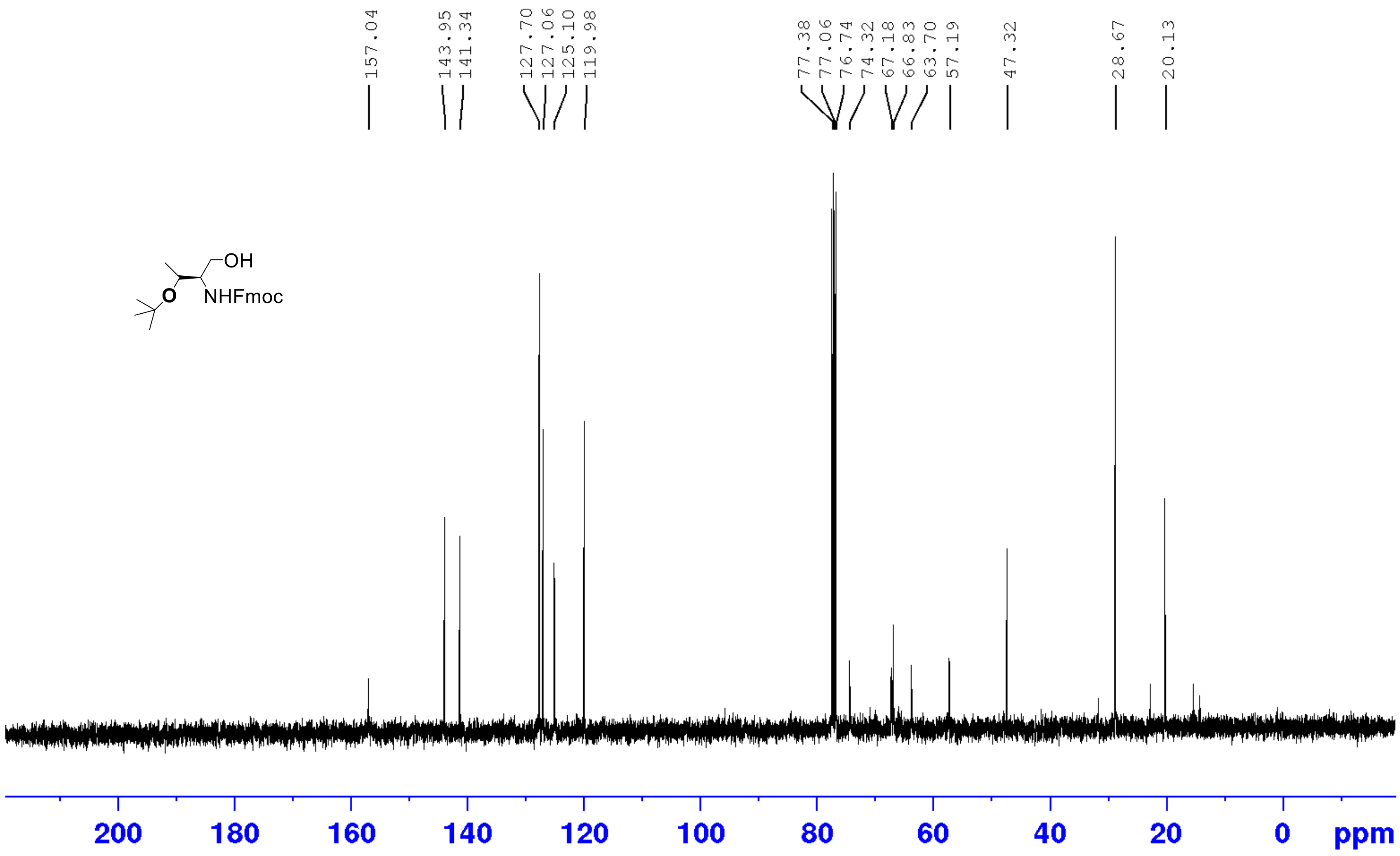

Figure S34. The ^{13}C NMR spectrum of compound **7** in $CDCl_3$, 298 K.

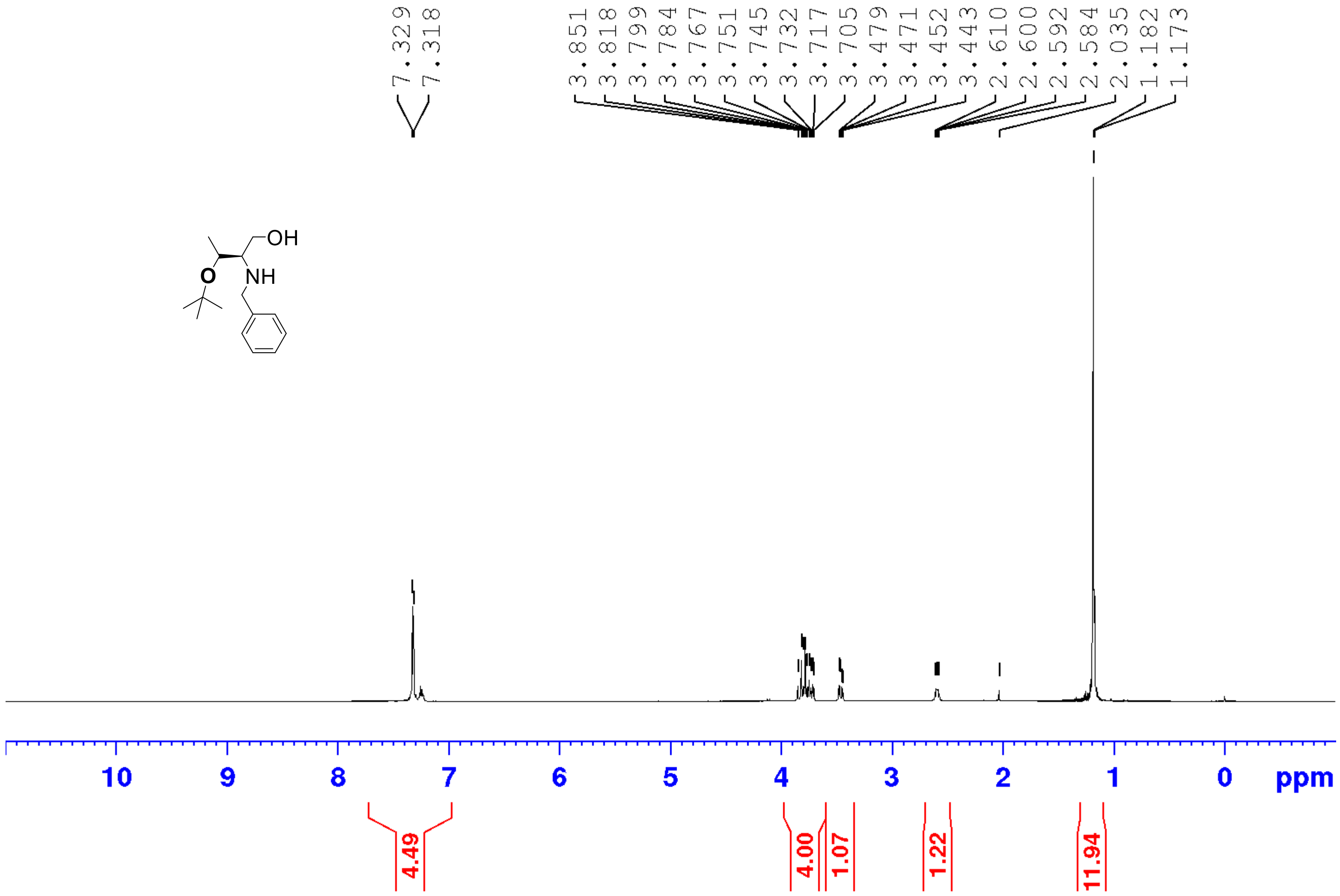

Figure S35. The ^{1}H NMR spectrum of compound **9** in $CDCl_3$, 298 K.

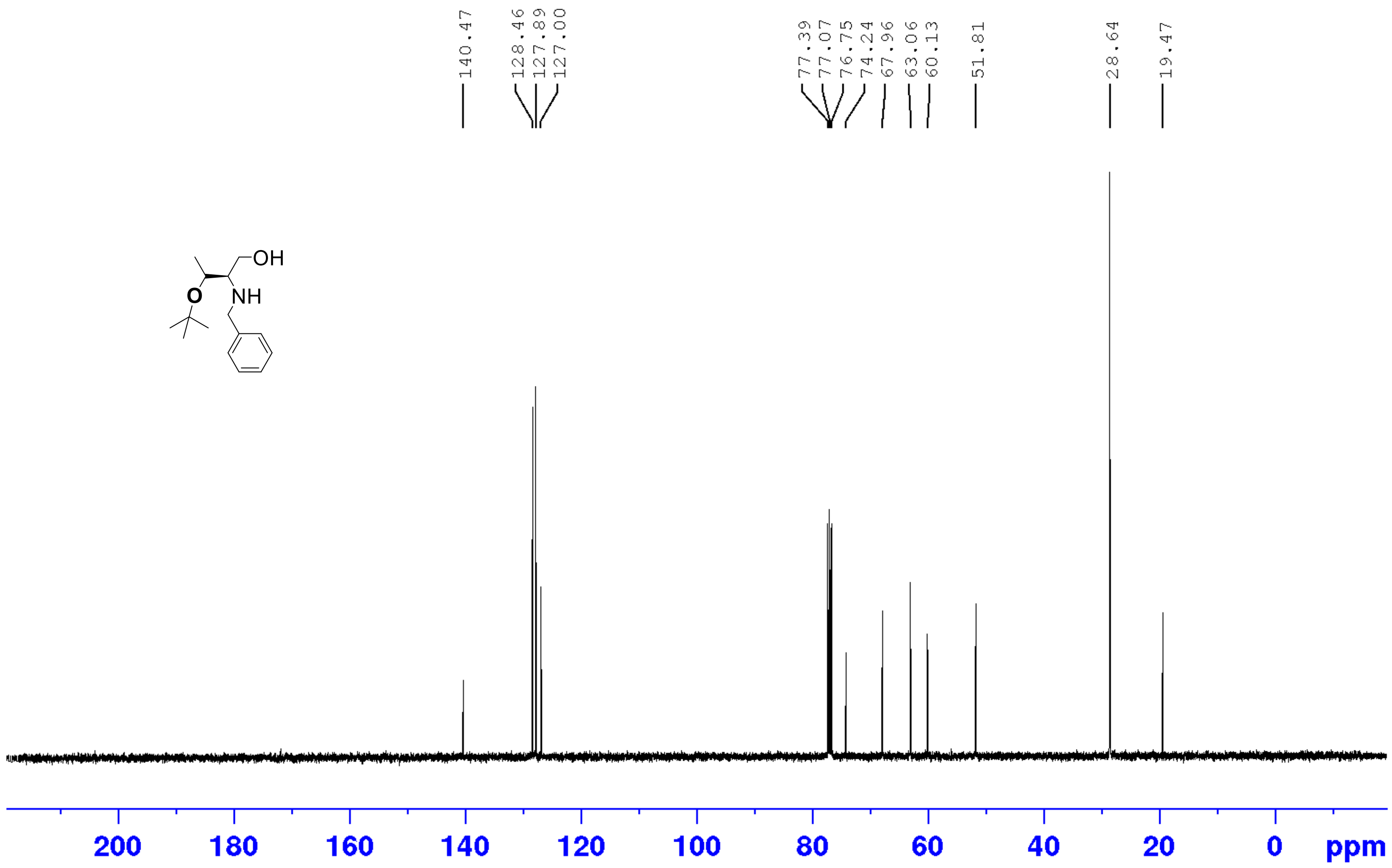

Figure S36. The ^{13}C NMR spectrum of compound **9** in $CDCl_3$, 298 K.

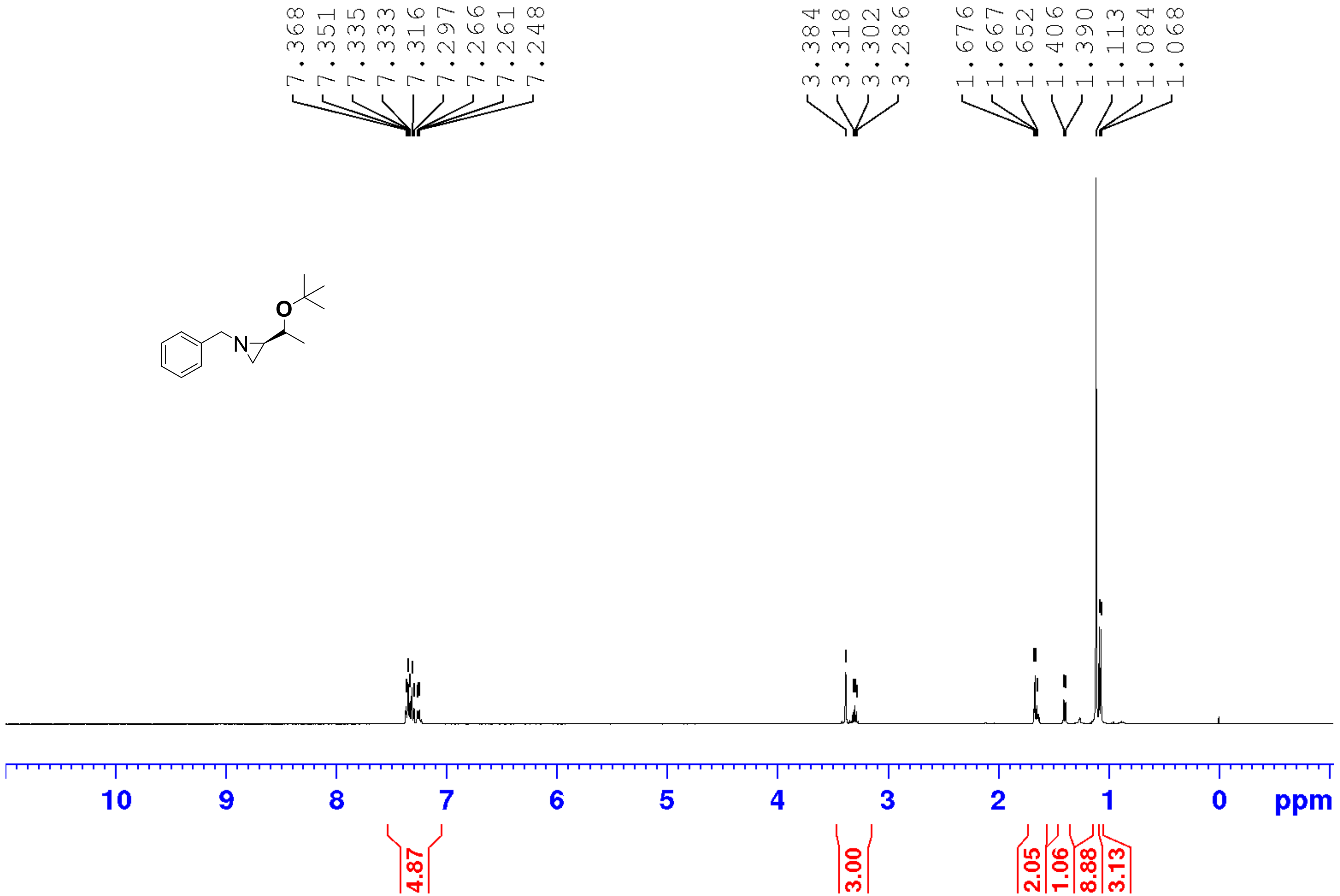

Figure S37. The ^{1}H NMR spectrum of compound **10** in $CDCl_3$, 298 K.

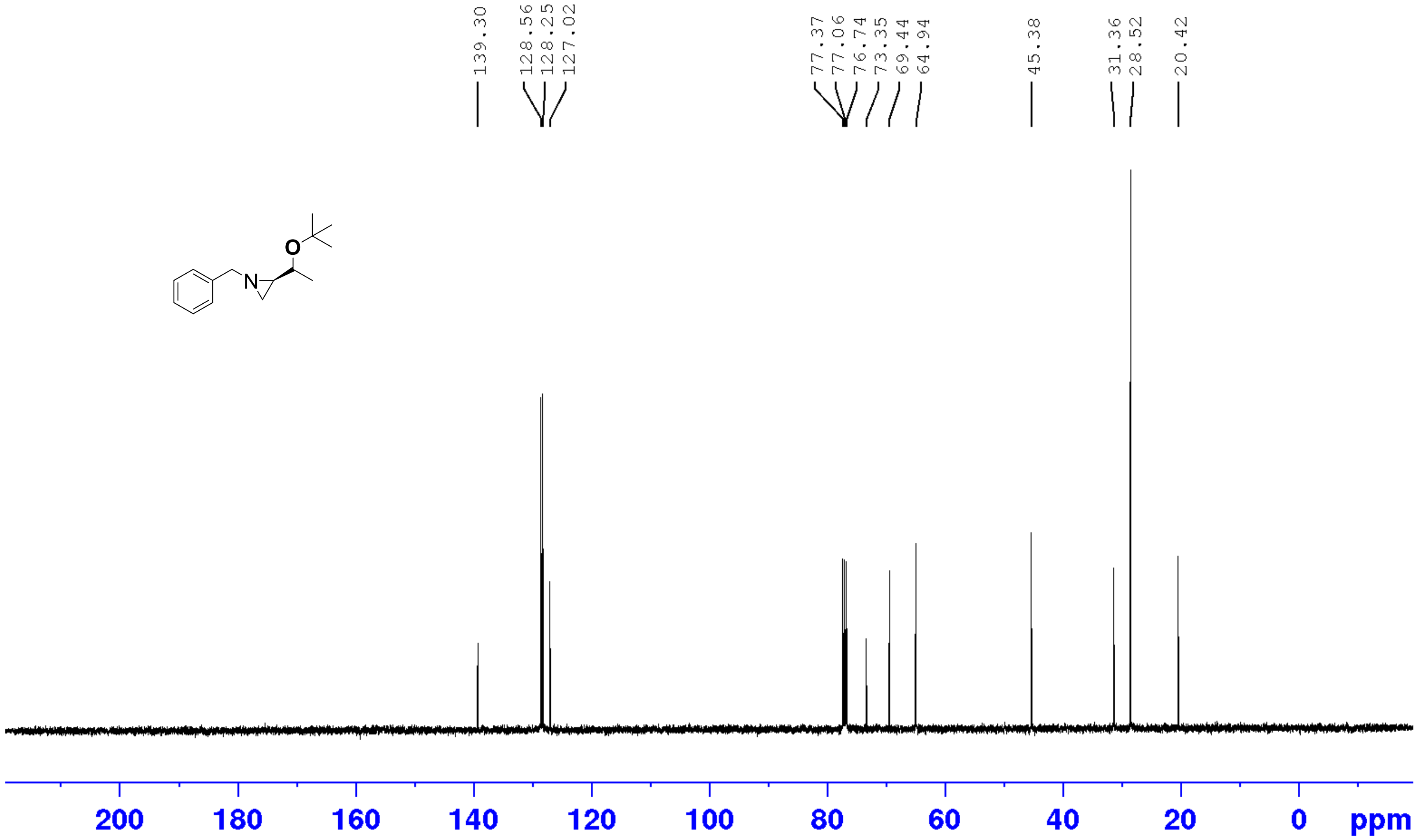

Figure S38. The ^{13}C NMR spectrum of compound **10** in $CDCl_3$, 298 K.

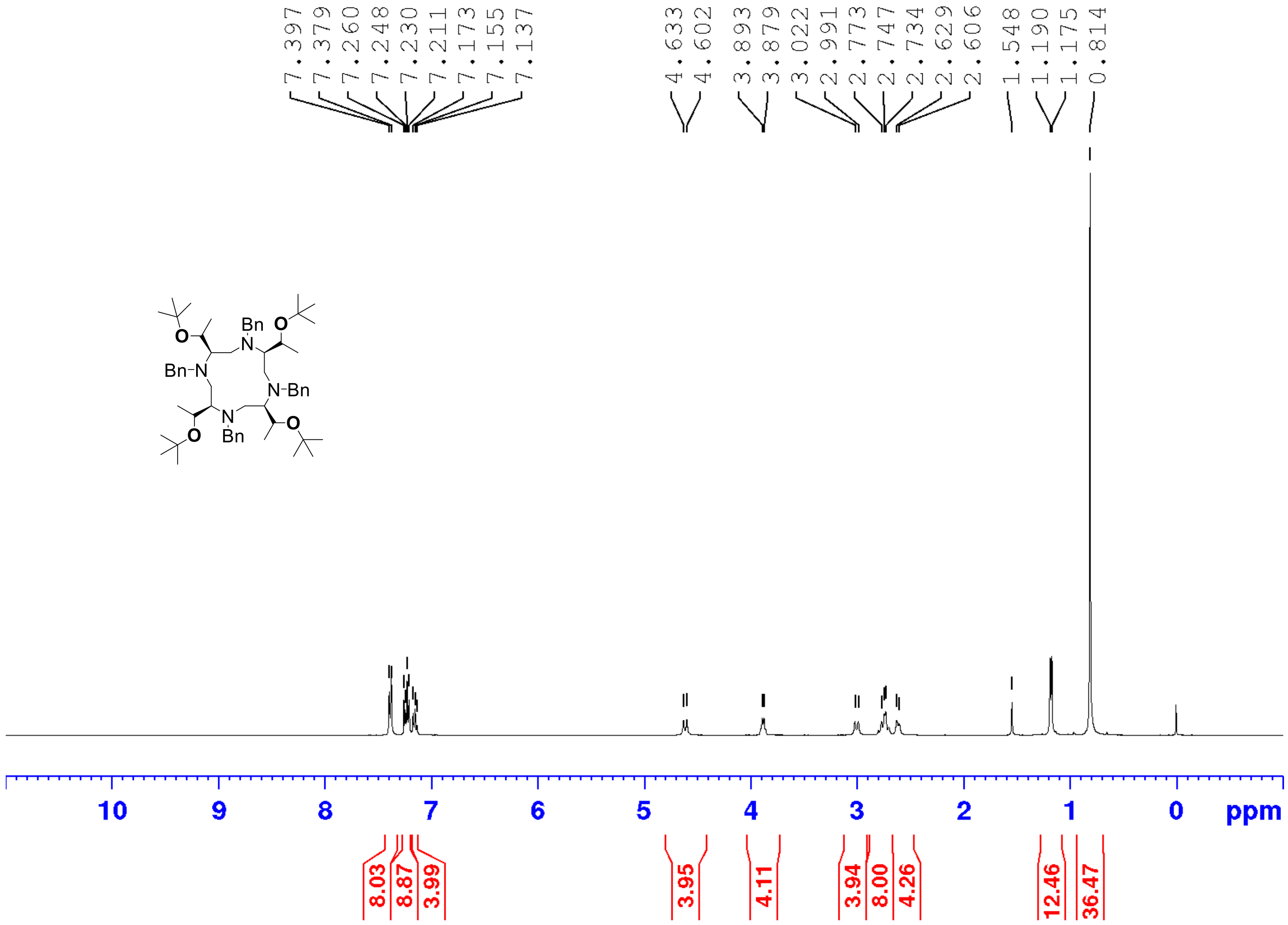

Figure S39. The ^{1}H NMR spectrum of compound **11** in $CDCl_3$, 298 K.

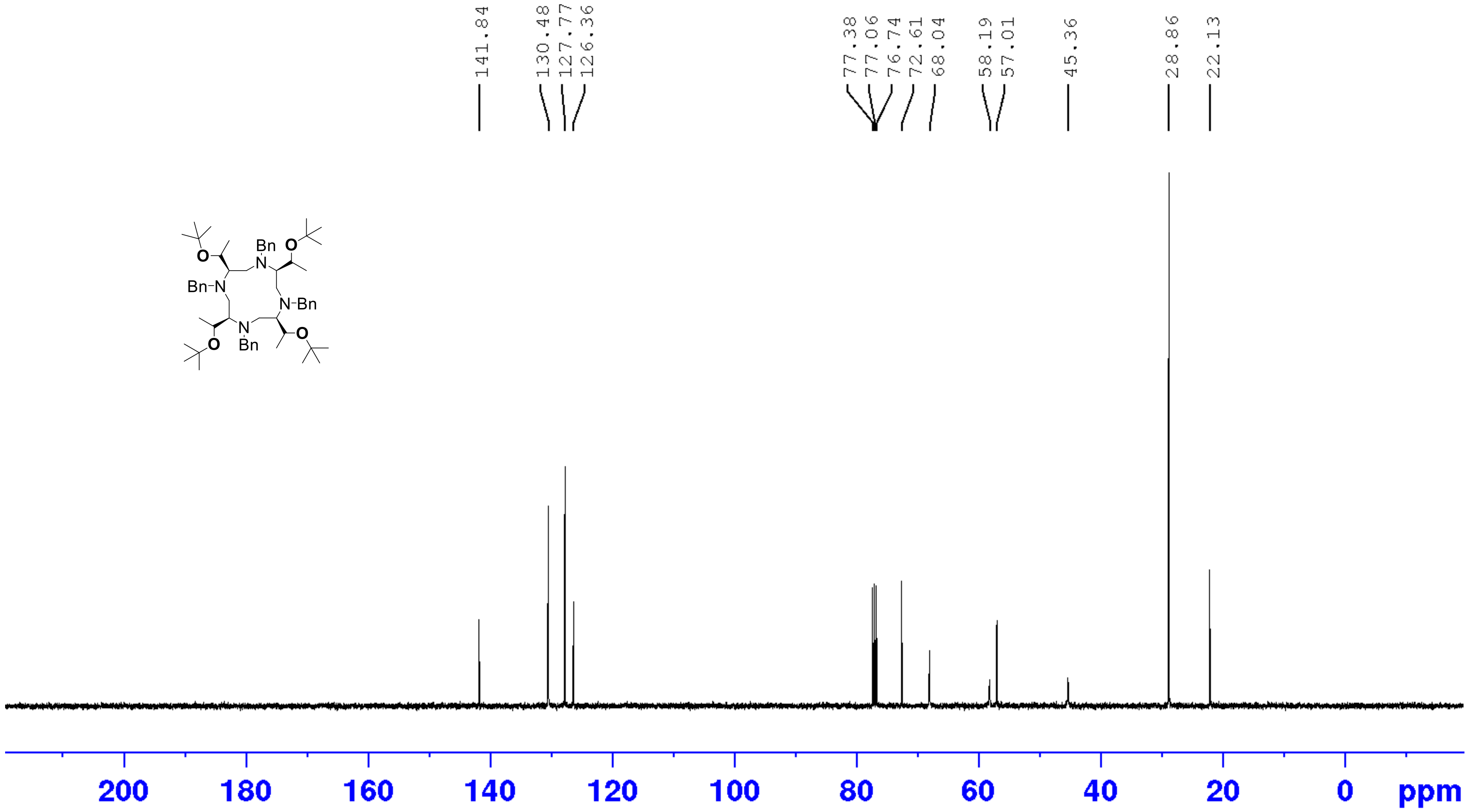

Figure S40. The ^{13}C NMR spectrum of compound **11** in $CDCl_3$, 298 K.

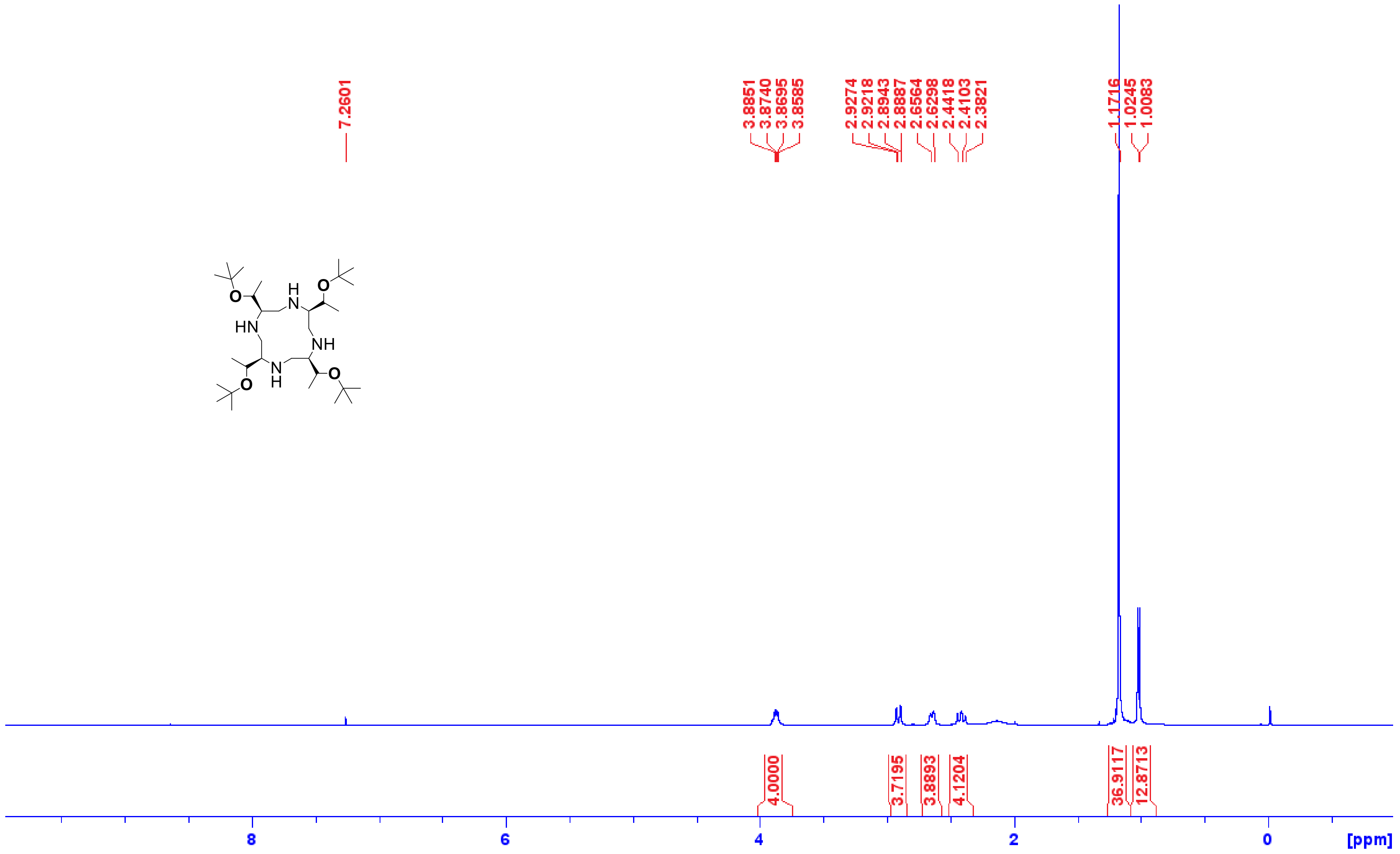

Figure S41. The ^{1}H NMR spectrum of compound **12** in $CDCl_3$, 298 K.

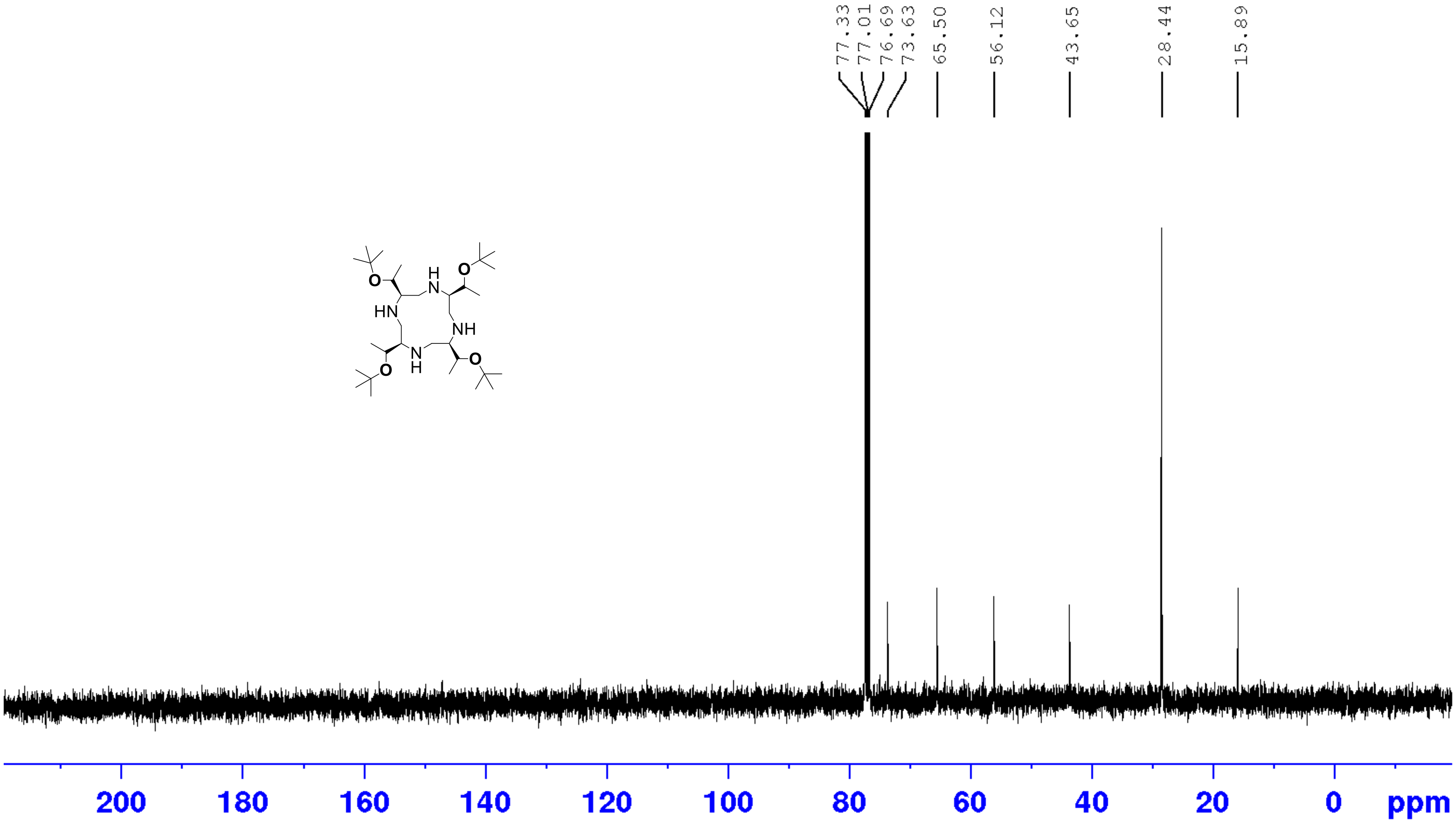

Figure S42. The ^{13}C NMR spectrum of compound **12** in $CDCl_3$, 298 K.

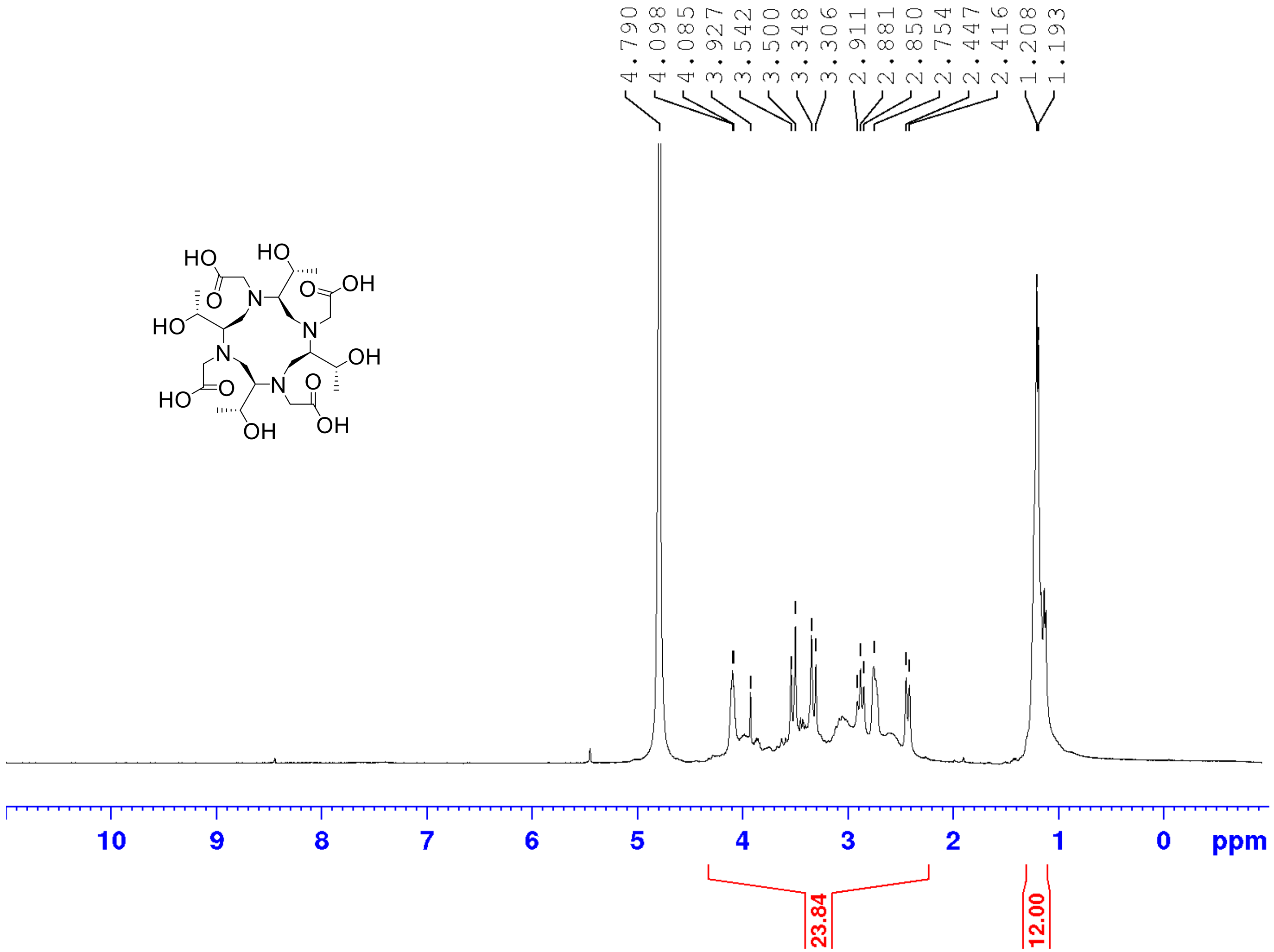

Figure S43. The ^{1}H NMR spectrum of compound **14** in D_2O, 298 K.

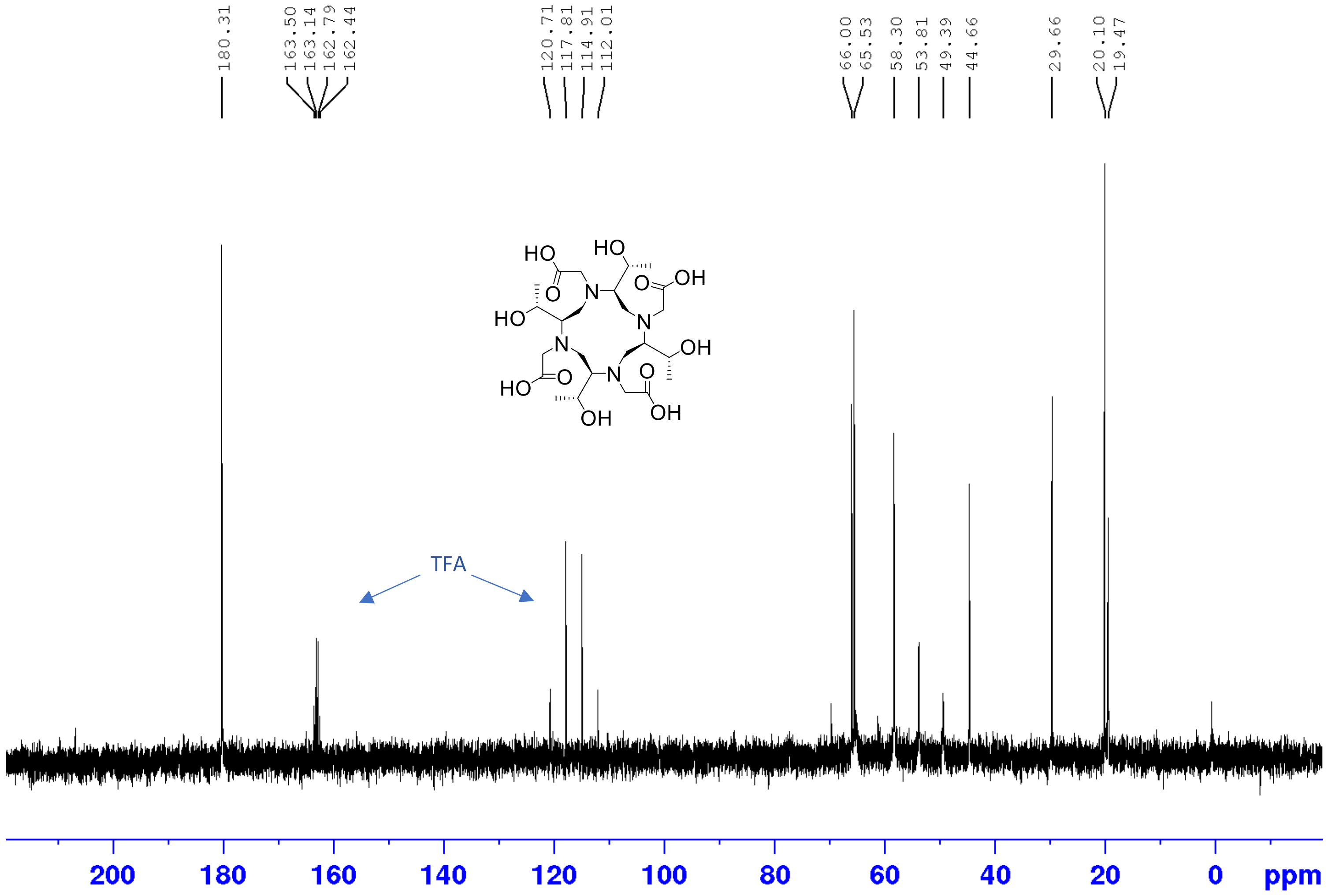

Figure S44. The ^{13}C NMR spectrum of compound **14** in D_2O, 298 K.

7. HRMS Spectra of Complexes

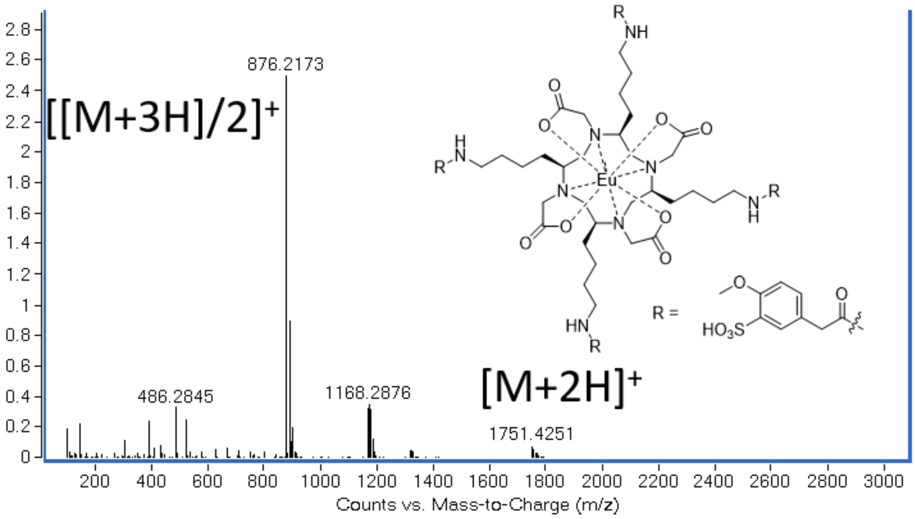

Figure S45. High-resolution mass spectrum of **Eu-LS**. *m/z* (ESI-MS$^+$) 1751.4251 ([M+2H]$^+$ calculated: 1751.4262).

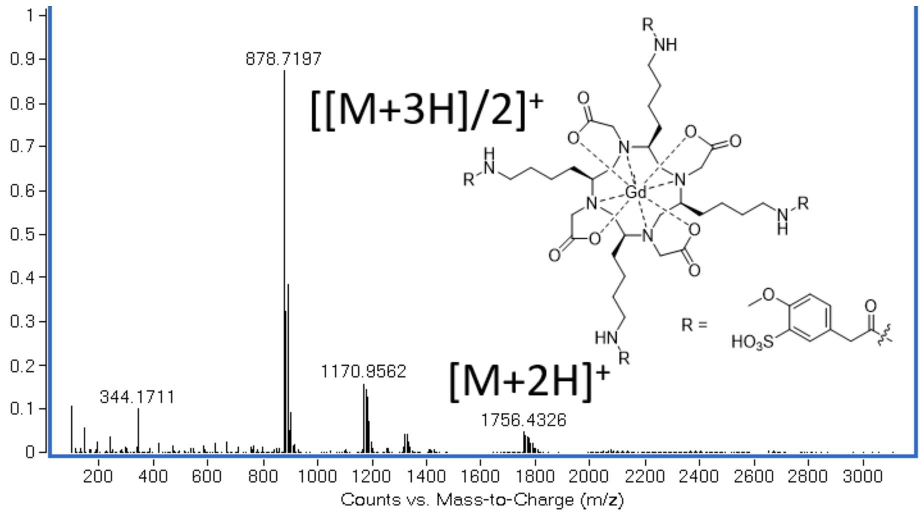

Figure S46. High-resolution mass spectrum of **Gd-LS**. *m/z* (ESI-MS$^+$) 1756.4326 ([M+2H]$^+$ calculated: 1756.4290).

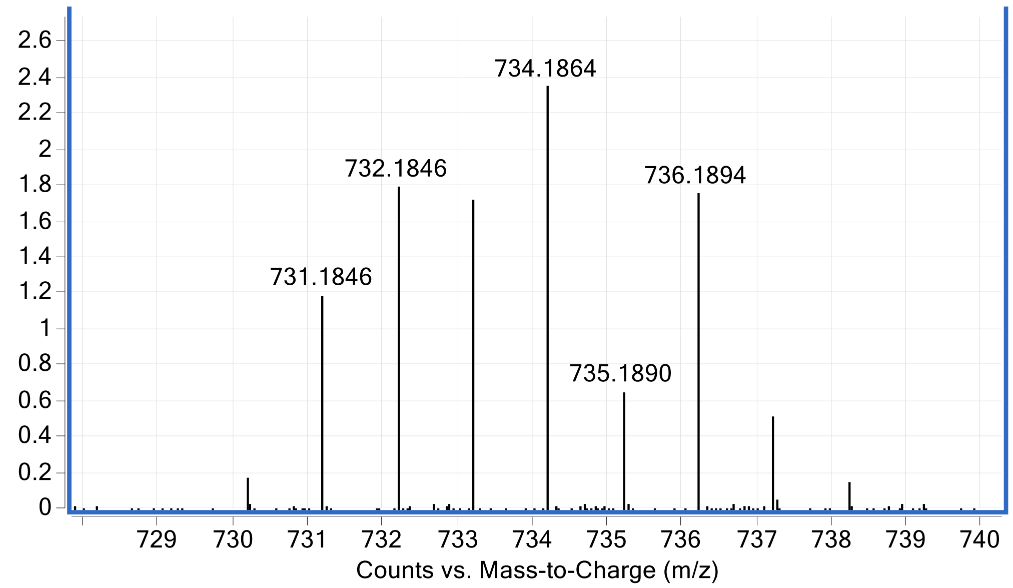

Figure S 47 High-resolution mass spectrum of **Gd-T**. *m*/*z* (ESI-MS$^-$) 734.1864 ([M]$^-$ calculated: 734.1884).

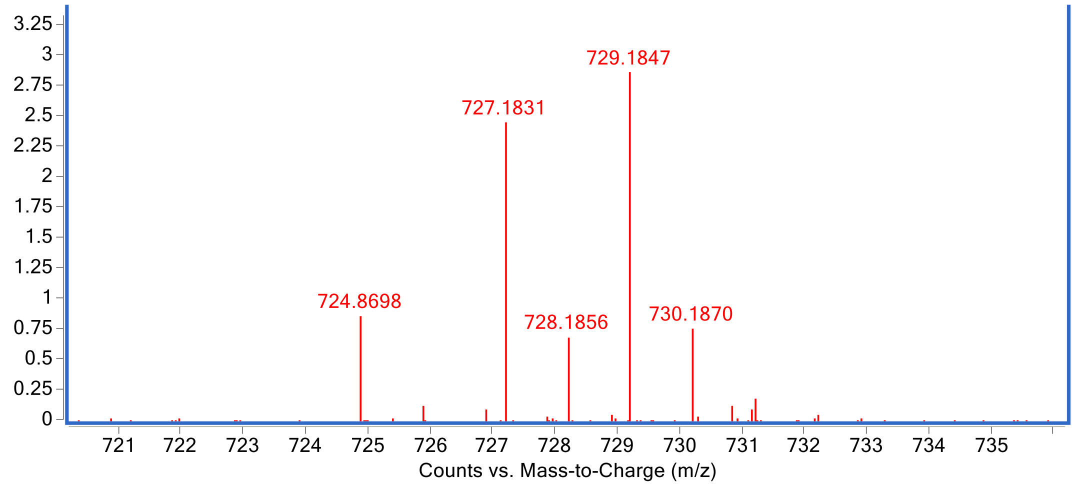

Figure S 48 High-resolution mass spectrum of **Eu-T**. *m*/*z* (ESI-MS$^-$) 729.1847 ([M]$^-$ calculated: 729.1855).

8. Lifetime Measurements

Table S 1 Q value of Eu-LS and Eu-T.

	(SAP) EuLS	(TSAP) EuLS	(SAP) EuT	(TSAP) EuT
Q value[1]	0.975	1.02	1.04	1.148
Q value[2]	0.835	0.878	0.893	0.995

[1] Q value calculated according to Parker's equations. [2] Q value calculated according to Horrocks' equations.

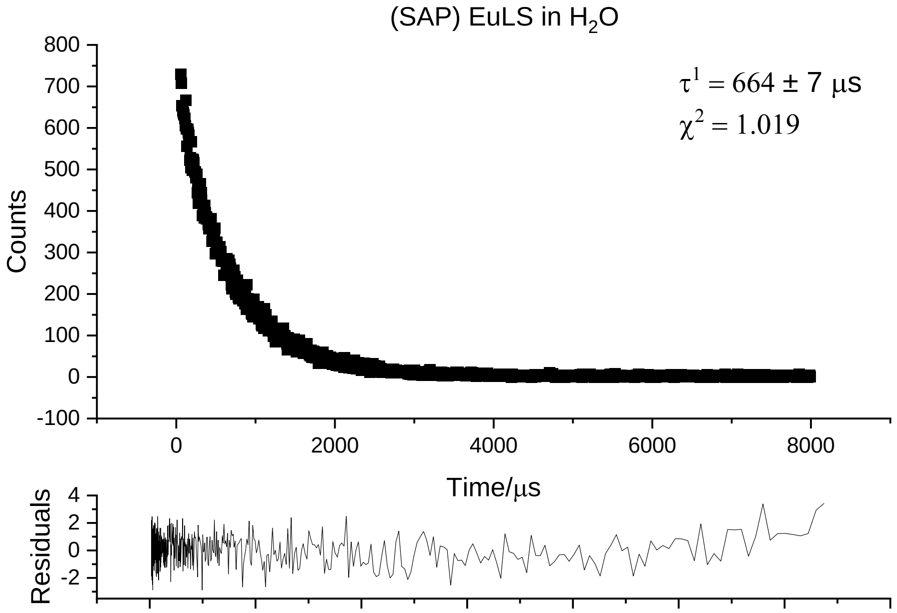

Figure S 49 Lifetime measurement for **(SAP) Eu-LS** in H_2O, ex=405.

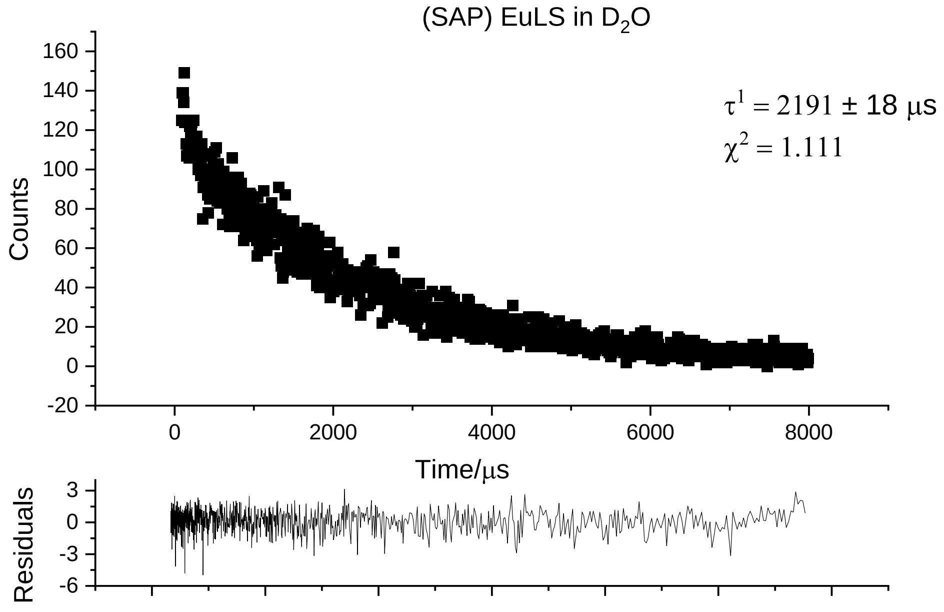

Figure S 50 Lifetime measurement for **(SAP) Eu-LS** in D_2O, ex=405.

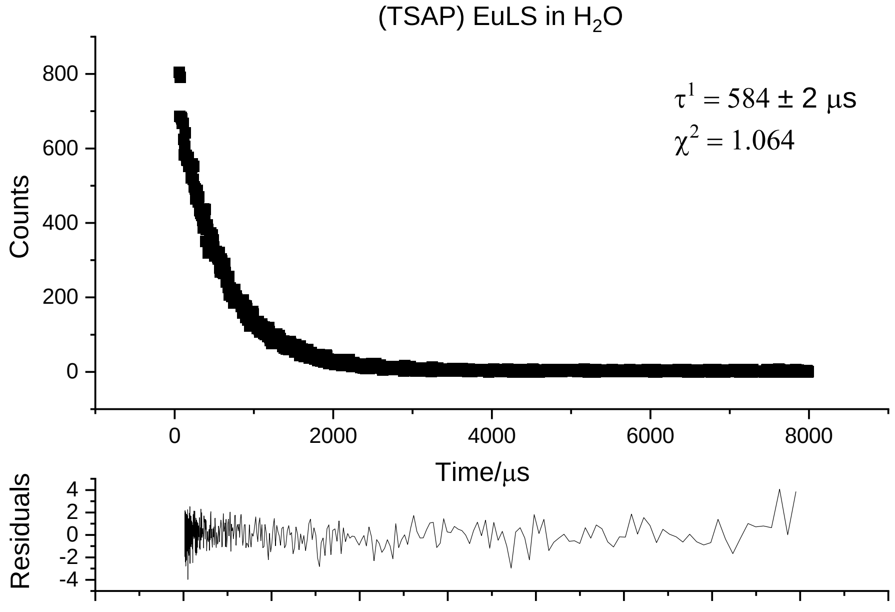

Figure S 51 Lifetime measurement for **(TSAP) Eu-LS** in H_2O, ex=405.

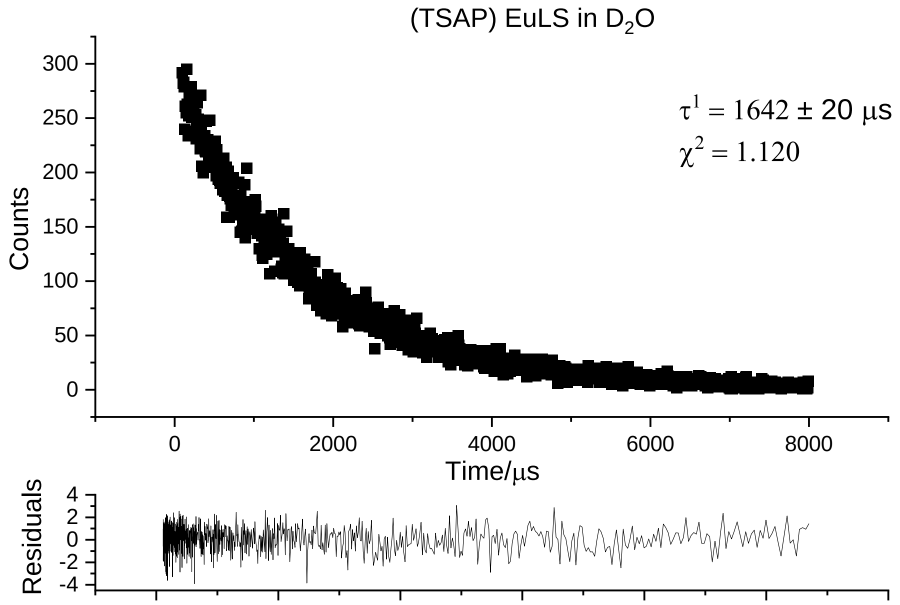

Figure S 52 Lifetime measurement for **(TSAP) Eu-LS** in D_2O, ex=405.

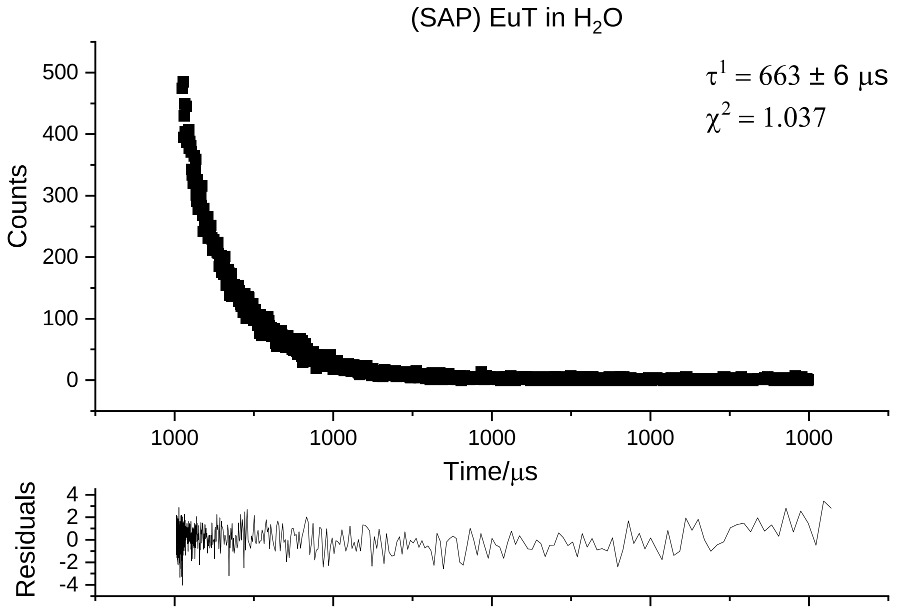

Figure S 53 Lifetime measurement for **(SAP) Eu-T** in H_2O, ex=405.

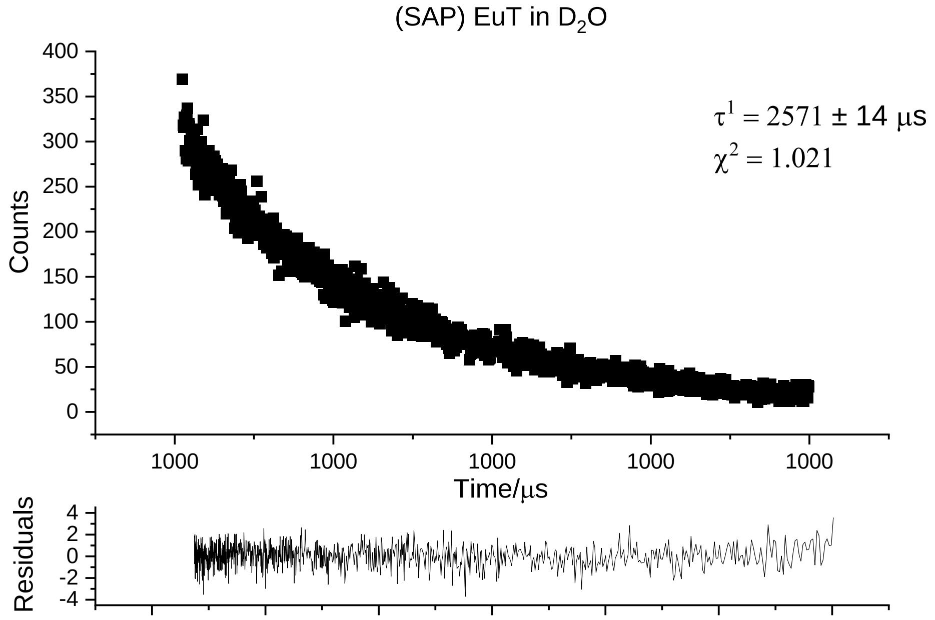

Figure S 54 Lifetime measurement for **(SAP) Eu-T** in D_2O, ex=405.

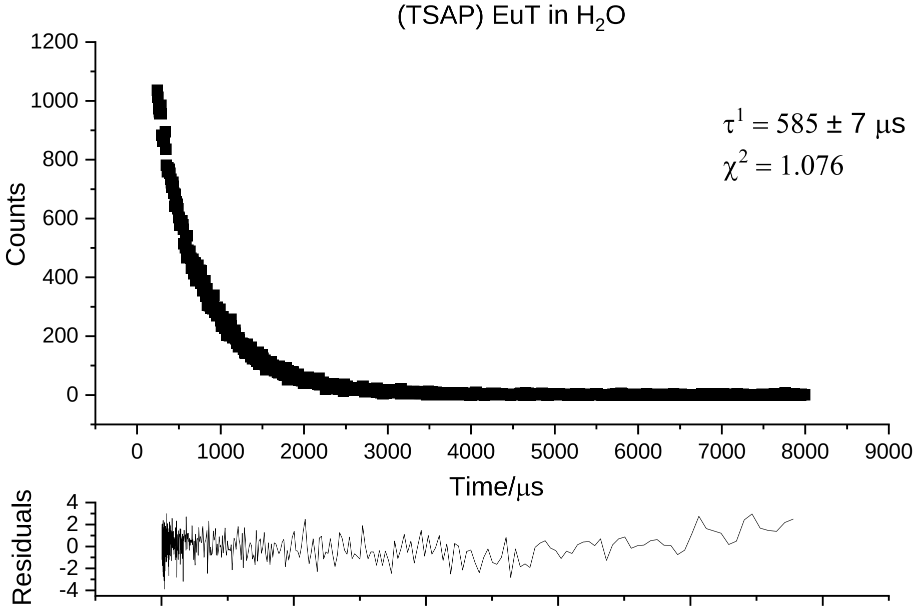

Figure S 55 Lifetime measurement for **(TSAP) Eu-T** in H_2O, ex=405.

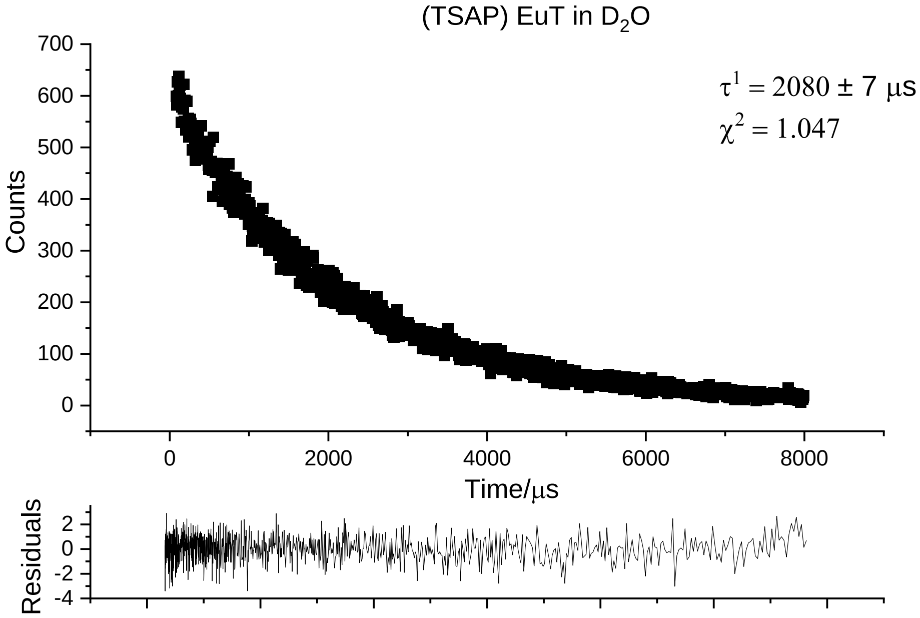

Figure S 56 Lifetime measurement for **(TSAP) Eu-T** in D_2O, ex=405.

9. Variable temperature ^{17}O NMR Measurements

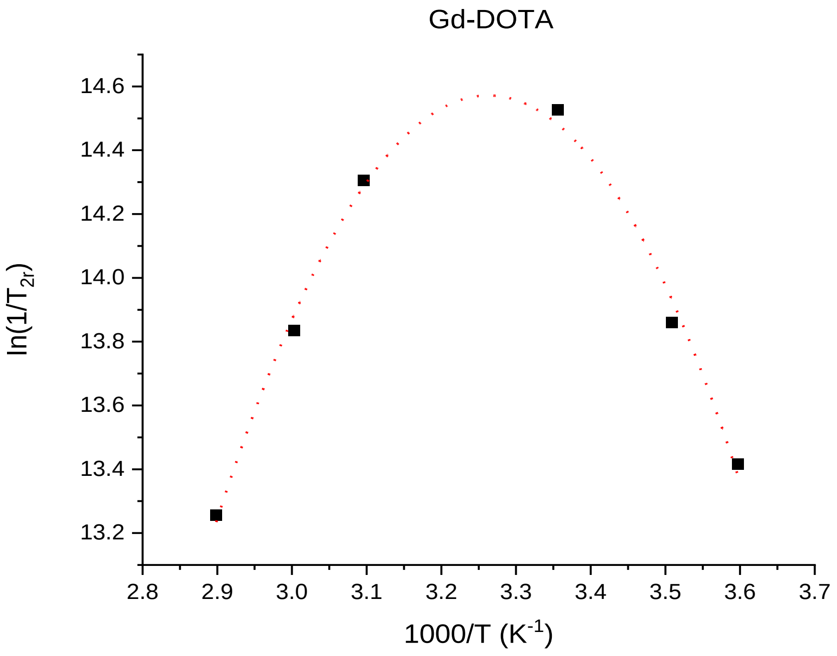

Figure S 57 Temperature dependence of the paramagnetic contribution to the transverse ^{17}O water relaxation rate of **Gd-DOTA** at 14.1 T with 5 % enriched $H_2{}^{17}O$.

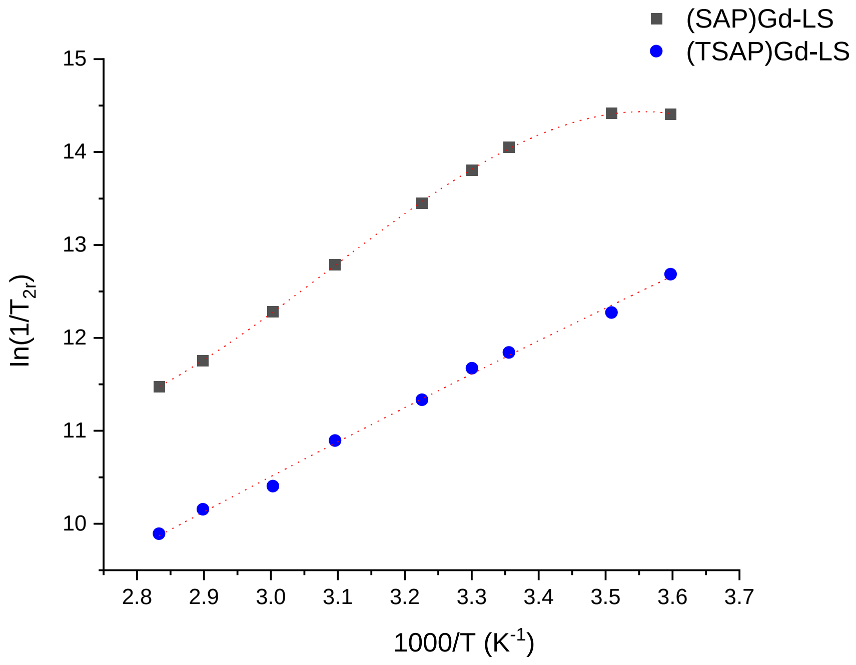

Figure S 58 Temperature dependence of the paramagnetic contribution to the transverse ^{17}O water relaxation rate of **(SAP)Gd-LS** and **(TSAP)Gd-LS** at 14.1 T with 5 % enriched $H_2{}^{17}O$.

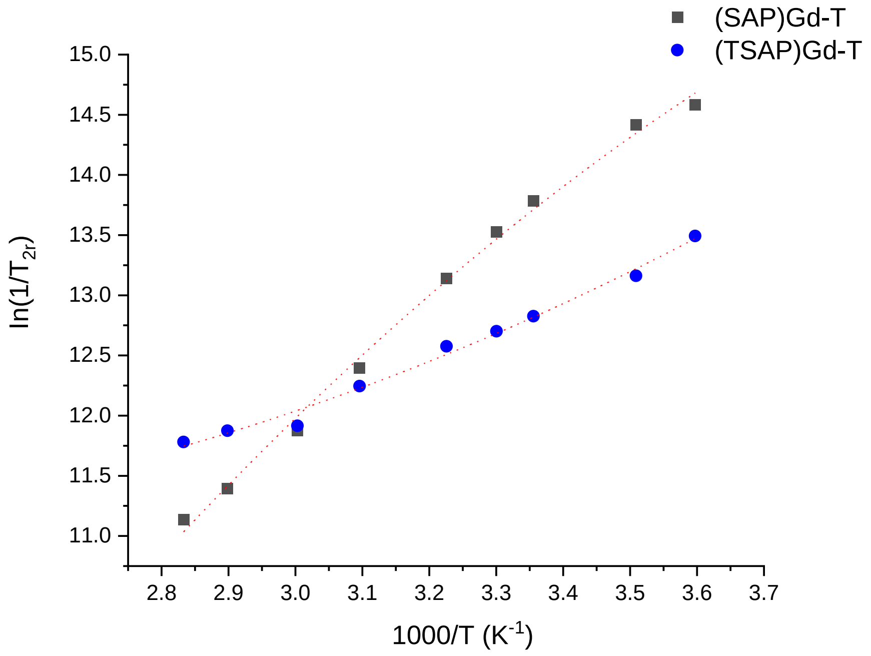

Figure S 59 Temperature dependence of the paramagnetic contribution to the transverse ^{17}O water relaxation rate of **(SAP)Gd-T** and **(TSAP)Gd-T** at 14.1 T with 5 % enriched $H_2{}^{17}O$.

10. Supplementary References

1. A. Beeby, I. M. Clarkson, R. S. Dickins, S. Faulkner, D. Parker, L. Royle, A. S. de Sousa, J. A. G. Williams, M. Woods, *J. Chem. Soc., Perkin Trans. 2.* **1999**, 493-503.
2. R. M. Supkowski, W. D. Horrocks, *Inorg. Chim. Acta.* **2002**, *340*, 44-48.
