# Supplementary material for: Design and synthesis of chiral DOTA-based MRI contrast agents with remarkable relaxivities
Source: Commun Chem. 2023 Nov 16;6:251. doi: 10.1038/s42004-023-01050-w (PMC10654417; doi:10.1038/s42004-023-01050-w)
Supplement: Supplementary file 2 — Description of Additional Supplementary Files [file 42004_2023_1050_MOESM2_ESM.pdf]

Description of Additional Supplementary Files

File name: Supplementary Data 1

Description: The cif file of crystal structure of Eu-T
